# Diterpenoids from the Aerial Parts of *Isodon serra* with Selective Cytotoxic Activity

**DOI:** 10.3390/molecules29122733

**Published:** 2024-06-08

**Authors:** Siqin Li, Fang Liang, Dongdong Huang, Huanling Wu, Xiaohua Tan, Jiang Ma, Caihong Wei, Shixiong Wang, Ziying Huang, Guang Yang, Xin He, Ji Yang

**Affiliations:** 1 School of Traditional Chinese Materia Medica, Guangdong Pharmaceutical University, Guangzhou 510006, China; lisiqin_email@163.com (S.L.); liangfang_email@163.com (F.L.); hdongdong@163.com (D.H.); wuhuanling980@163.com (H.W.); ycyjzhongxin@163.com (X.T.); majiang@gdpu.edu.cn (J.M.); weicaihong1212@163.com (C.W.); hnwangshixiong@163.com (S.W.); ziying_huang2024@163.com (Z.H.); 2 China Academy of Chinese·Medical Sciences, Beijing 100700, China; hbykdxyg2008@163.com

**Keywords:** *Isodon serra*, diterpenoids, cytotoxic activity, structure elucidation

## Abstract

Four new diterpenoids, isodosins A–D (**1**–**4**), together with nine known compounds (**5**–**13**) were isolated and identified from the aerial parts of *Isodon serra* (Maxim.) Hara. The structures of the new diterpenoids were elucidated based on the analysis of HR-ESI-MS data, 1D/2D-NMR-spectroscopic data, and electronic circular dichroism (ECD) calculations. Cytotoxicities of compounds **2**, **3**, **5**, **6**, and **9** against the HepG2 and H1975 cell lines were evaluated with the MTT assay. As a result, compounds **2**, **3**, and **6** revealed higher levels of cytotoxicity against HepG2 cells than against H1975 cells. Moreover, compund **6** demonstrated the most efficacy in inhibiting the proliferation of HepG2 cells, with an IC_50_ value of 41.13 ± 3.49 μM. This effect was achieved by inducing apoptosis in a dose-dependent manner. Furthermore, the relationships between the structures and activities of these compounds are briefly discussed.

## 1. Introduction

Hepatocellular carcinoma (HCC), which is the most common primary tumor of the liver, is ranked as the third most common cause of cancer death in 46 countries, thus remaining a global health challenge [1,2]. The number of new cases and deaths from HCC is predicted to rise by more than 55% from 2020 to 2040, resulting in 1.3 million fatalities in 2040 [1]. The risk of developing HCC has been associated with multiple factors, including chronic viral infections [3], alcohol consumption [4], and exposure to various chemicals [5]. The development of chemotherapy has positively influenced the treatment of HCC and offers a substantial improvement in patient survival [6] from drugs such as Sorafenib, 5-Fluorouracil, and Cisplatin [7]. However, the main limitation of the chemotherapeutic modality is their non-specificity and their inability to identify and target cancerous cells directly [8,9]. The administration of anti-HCC chemotherapeutics often necessitates the utilization of delivery systems to ensure targeted drug delivery, thereby minimizing systemic side effects [7,10]. Consequently, medicinal chemists have been making efforts to find potential chemotherapeutic agents with high selectivity and low toxicity effects over the past several decades [11].

Natural products (NPs) from medicinal plants play a crucial role in the exploration of new drugs for cancer therapy [12]. Among the different types of NPs, diterpenoids have attracted considerable interest on account of their promising anti-cancer effects and modifiable skeleton, including NPs such as Taxol and Ingenol 3-angelate [13,14,15,16,17]. Recently, researchers have discovered a series of diterpenoids from medicinal plants with selective anti-cancer activities and have proved that those diterpenoids could induce apoptosis, autophagy, and metastasis suppression in cancer cells via the inhibition of Akt [18,19,20]. These findings suggest that the isolation of more diterpenoids with selective cytotoxic activities from medicinal plants could make a substantial contribution to the progress of anti-cancer drugs [21].

*Isodon serra* (Maxim.) Hara, a perennial plant belonging to the family of Labiatae, is commonly used as a folk medicine for the treatment of acute jaundice, hepatitis, and acute cholecystitis [22,23,24]. Previous phytochemical studies on *I. serra* have led to the isolation and identification of over 90 *ent*-kaurane diterpenoids, which are characterized by a perhydrophenanthrene subunit (A, B, and C rings) and a cyclopentane ring (D ring) [25]. Pharmacological investigations have been conducted to highlight the therapeutic potential of *ent*-kaurane compounds isolated from *I. serra* against various cancers such as liver [22,26], colon [27,28], lung [29], muscle-invasive bladder [30], and human nasopharyngeal carcinoma [31] cancer. In an effort to search for diterpenoids with anti-liver cancer bioactivity from *I. serra*, the petroleum ether fraction of this plant was chemically investigated.

**Figure 1:**
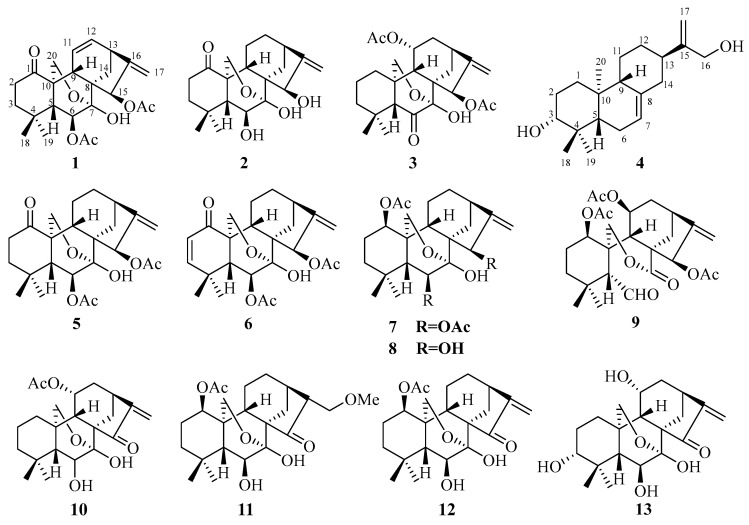
Chemical structures of compounds **1**–**13**.

In this study, four new diterpenoids (**1**–**4**) and nine known ones (**5**–**13**) were isolated and characterized (Figure 1). Compounds **2**, **3**, and **6** demonstrate high selectivity towards HepG2 cells over H1975 cells, which could be attributed to the presence of a double bond at C-2 and C-3 or the hydroxy groups at C-6 and C-15. Furthermore, compound **6** exhibited a dose-dependent induction of apoptosis in HepG2 cells.

## 2. Results and Discussion

### 2.1. Isolation and Identification of Diterpenoids from I. serra

**Figure 2:**
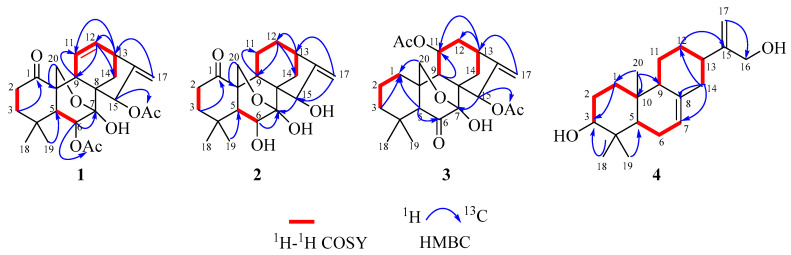
^1^H-^1^H COSY and key HMBC correlations of compound **1**–**4**.

**Figure 3:**
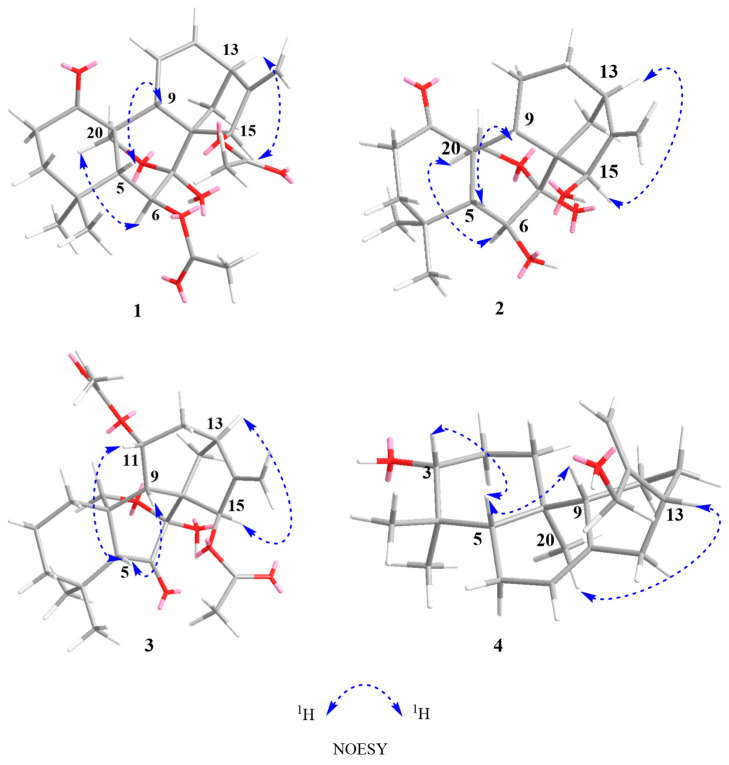
Key NOESY correlations of compounds **1**–**4**.

**Figure 4:**
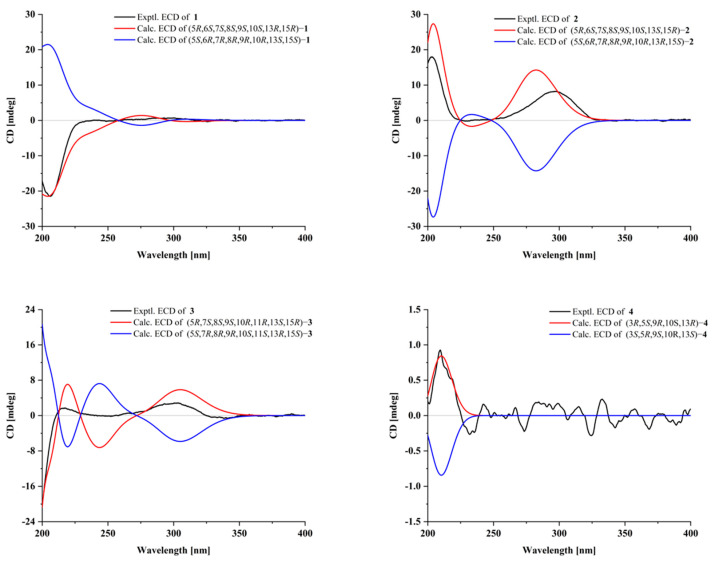
Experimental and calculated ECD spectra of compounds **1**–**4**.

Compound **1** was acquired as a white powder with a molecular formula of C_24_H_30_O_7_, as determined by the HR-ESI-MS ion at *m*/*z* 453.1888 [M + Na]^+^ (calculated for C_24_H_30_O_7_Na, 453.1884), corresponding to ten degrees of unsaturation. The IR absorptions at 2927, 1736, and 1579 cm^−1^ suggested the presence of hydroxy, carbonyl, and double bond functionalities, respectively (Figure S8). Its ^1^H NMR spectrum (Table 1) showed four methyl singlets at *δ*_H_ 0.92 (s), 1.10 (s), 2.21 (s), and 2.08 (s); two protons of oxygenated methylene at *δ*_H_ 4.10 (d, *J* = 9.9 Hz) and 3.98 (dd, *J* = 9.9, 3.0 Hz); two protons of oxygenated methines at *δ*_H_ 5.14 (d, *J* = 7.1 Hz) and 5.90 (t, *J* = 2.1 Hz); and four olefinic protons at *δ*_H_ 4.86 (brs) and 5.00 (d, *J* = 2.0 Hz), 5.58 (dd, *J* = 2.4, 10.0 Hz), and 6.37 (m). The ^13^C, DEPT, and HSQC NMR spectra of compound 1 gave 24 carbon signals comprising four methyls, five methylenes, seven methines, and eight quaternary carbons (Table 1), which was consistent with a skeleton of a 7, 20-epoxy-ent-kaur-16-en-1-one [24,25,26,32,33]. The signals at *δ*_C_ 170.3, 21.9, 173.8, and 21.0, along with methyl singlets (*δ*_H_ 2.20 and 2.08), indicated the presence of two acetoxy groups. The two acetyl groups were assigned to C-6 and C-15 by the HMBC correlations from H-6 (*δ*_H_ 5.14) and *δ*_H_ 2.08 to *δ*_C_ 170.3 and H-15 (*δ*_H_ 5.90) and *δ*_H_ 2.20 to *δ*_C_ 173.8, respectively. The carbonyl (*δ*_C_ 211.0) was placed at C-1 by the HMBC correlations of H-2/H-3/H-20 with carbonyl (Figure 2). The NMR information from compound **1** suggests that it is very similar to maoecrystal D (**5**) [34], except that the two methines of C-11/C-12 (*δ*_C_ 17.2 and 31.8) in maoecrystal D (**5**) were replaced by a double bond (*δ*_C_ 124.3 and 138.0) in compound **1**. This hypothesis was confirmed by the ^1^H-^1^H COSY correlations of H-10/H-11/H-12/H-13 and the HMBC correlations of H-13/H-14 with C-12 and H-9/H-12 with C-11(Figure 2). Furthermore, the hydroxyl group at C-6 was assigned a *β*-orientation based on the NOESY correlations of H-6 with C-19 and C-18. The NOESY cross-peak of H-15/H-13, as well as the small coupling constant of H-15 (*δ*_H_ 5.90, t, *J* = 2.1 Hz), suggested a *β*-orientation of the acetoxy group at C-15 (Figure 3). The absolute configuration of (5*R*,6*S*,7*S*,8*S*,9*S*,10*S*,13*S*,15*R*)−**1** was determined by performing ECD calculations. The calculated ECD curve exhibited identical Cotton effects (CEs) to those observed in the experimental curve (Figure 4). Consequently, the structure of **1** was characterized as 6*β*,15*β*-diacetoxy-7*α*,20-epoxy-ent-kaur-16-en-1-one, which has been named isodosin A.

Compound **2**, a white powder, gave a molecular formula of C_20_H_28_O_5_ based on its HR-ESI-MS ion at *m*/*z* 349.2011 [M + H]^+^ (calculated for C_20_H_29_O_5_, 349.2010), corresponding to seven degrees of unsaturation. The ^1^H and ^13^C NMR data of compound 2 (Table 1) closely resembled those of maoecrystal D (**5**) [34], and the major difference was that the acetyl groups (*δ*_C_ 173.6, 170.1, 21.0, and 22.0) at C-6/C-15 in maoecrystal D (**5**) had disappeared in compound **2**. This hypothesis was confirmed by HMBC correlations from H-6 to C-4/C-5/C-7/C-8, and from H-15 to C-9/C-7/C-17/C-16 (Figure 2). The crucial NOESY cross-peak of H-6/H-20 and H-15/H-13 suggested *β*-orientations of the hydroxyl groups at C-6 and C-15 (Figure 3). The ECD curve of compound **2** showed a positive Cotton effect at 203 nm and 297 nm, which was consistent with the calculated ECD spectrum of (5*R*,6*S*,7*S*,8*S*,9*S*,10*S*,13*R*,15*R*)−2 (Figure 4). Accordingly, the absolute configuration of compound **2** was determined as 5*R*,6*S*,7*S*,8*S*,9*S*,10*S*,13*R*,15*R*. Thus, the structure of compound **2** was characterized as 6*β*,15*β*-diahydroxyl-7*α*,20-epoxy-ent-kaur-16-en-1-one, which has been named isodosin B.

Compound **3** had a molecular formula of C_24_H_32_O_7_ as deduced based on HR-ESIMS data (*m*/*z* 433.2224 [M + H]^+^, calculated for C_24_H_33_O_7_, 433.2221), with nine degrees of unsaturation. The ^1^H and ^13^C NMR data of 3 (Table 1) were superimposable on those of Parvifolin G [26]. Carefully comparing their 1D NMR spectrum indicated that the oxygenated methine (C-1) in Parvifolin G was replaced as methylene (*δ*_C_ 30.3) in compound **3**, and the acetyl group (*δ*_C_ 169.2 and 21.6) at C-1 of Parvifolin G was absent in compound **3** [26]. Then, the 2D structure of compound **3** was determined by ^1^H-^1^H COSY and HMBC NMR data analysis (Figure 2). In the NOESY spectrum, correlations of H-11/H-5 and H-15/H-13 indicated the α-orientation of OAc-11 and *β*-orientation of OAc-15. The absolute configuration of compound **3** was determined as 5*R*,7*S*,8*S*,9*S*,10*R*,11*R*,13*S*,15*R* by employing ECD calculations (Figure 4). Thus, the structure of compound **3** was formulated as 11*α*,15*β*-diacetoxy-7*α*,20-epoxy-ent-kaur-16-en-6-one, and it has been named isodosin C.

Compound **4** was obtained as a white amorphous powder. The HR-ESI-MS of 4 exhibited a [M + H]^+^ peak at 305.2471 (calculated 305.2475), which suggested a molecular formula of C_20_H_32_O_2_, indicating five degrees of unsaturation. Its IR spectrum had absorption bands at 3392, and 1594 cm^−1^, accounting for the presence of hydroxyl and double bond functionalities (Figure S38). The ^1^H NMR data of **4** (Table 1) showed three singlet methyl signals, at *δ*_H_ 0.81 (3H, s), 0.89 (3H, s), and 1.00 (3H, s) and four olefinic protons at *δ*_H_ 4.12 (2H, s), 4.88 (1H, s) and 5.03 (dd, *J* =1.5, 2.4 Hz). The ^13^C NMR and DEPT 135 spectra of **4** (Table 1) revealed 20 carbons, including three methyls (*δ*_C_ 28.4, 15.6, 15.0), eight methylenes (*δ*_C_ 37.9, 27.4, 23.0, 25.9, 31.6, 40.8, 65.2, and 107.8), five methines (δ_C_ 120.4, 79.3, 49.9, 52.3, and 41.2) and four quaternary carbons (*δ*_C_ 153.6, 38.6, 35.3, and 136.8). The 1H and ^13^C NMR data of compound 4 were superimposable on those of ent-abieta-7,15(17)-diene-3*β*,16,18-triol [35,36,37], except that the oxygenated methylene methyl at *δ*_C_ 68.0 in ent-abieta-7,15(17)-diene-3*β*,16,18-triol was reduced to a methyl (*δ*_C_ 28.4) in compound **4**, which was confirmed by HMBC correlations from H-18 to C-3/C-4/C-5. The hydroxyl group at C-3 was assigned an α-orientation from the NOESY interactions of H-3/H-5 (Figure 3). Furthermore, the absolute configuration of (3*R*,5*S*,9*R*,10*S*,13*R*)−**4** was determined using ECD calculations (Figure 4). Thus, the structure of compound 4 was determined as ent-abieta-7,15(17)-diene-3*α*,16-triol, which has been named isodosin D.

The known compounds were identified by comparison of their spectroscopic data with those reported in the literature as maoecrystal D (**5**) [34], odonicin (**6**) [38], enmenin monoacetate (**7**) [39], maoecrystal E (**8**) [40], shikokianal acetate (**9**) [41], longikaurin E (**10**) [42], nervosanin A 1-acetate (**11**) [43], effusanin B (**12**) [44], and taibaihenryiin B (**13**) [45].

### 2.2. Compounds Isolated from I. serra Were Selectively Toxic toward HepG2 over H1975 Cells

The global prevalence of HCC is increasing, posing a significant challenge to public health [46]. Chemotherapy has emerged as a well-established therapeutic approach in cancer treatment, offering potential relief from the disease burden. The superior efficacy and safety profile of targeted therapeutic drugs, in comparison to traditional chemotherapy, has established them as the prevailing modality for cancer treatment [8]. *I. serra* is a proven source of diterpenoid-containing natural products that often have anti-cancer activity [28].

**Figure 5:**
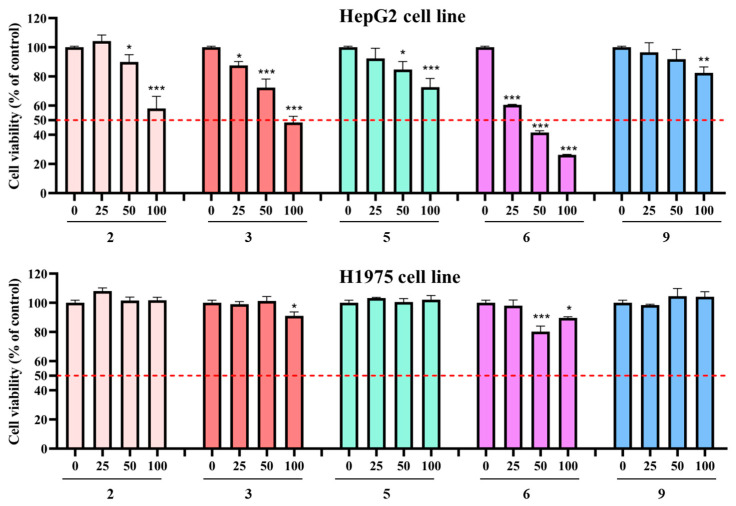
Cytotoxic activities of compounds **2**, **3**, **5**, **6**, and **9** against HepG2 cells and H1975 cells cell lines. Data were collected in three replicates. Error bars represent standard deviation. *—*p* < 0.05 versus the control group; **—*p* < 0.01 versus the control group; ***—*p* < 0.001 versus the control group.

In consideration of the anti-cancer activity of *ent*-kaurane diterpenoids, the main compounds **2**, **3**, **5**, **6**, and **9** were evaluated for their cytotoxic activities against two human cancer cell lines (HepG2 and H1975) by an MTT method. As shown in Figure 4, it is noteworthy that compounds **2**, **3**, and **6** exhibited significant inhibitory effects against HepG2 cells while displaying limited cytotoxic activity against H1975 cells. Compound **6** exhibited strong inhibition effects (IC_50_ = 41.13 ± 3.49 μM) on HepG2 cells, whereas compounds **2** and **3** demonstrated potent cytotoxic activity, displaying IC_50_ values of 121.33 ± 17.54 μM and 96.44 ± 9.52 μM, respectively. In contrast, compounds **5** and **9** exhibited relatively weak cytotoxic activity against HepG2 cells. Importantly, the tested compounds exhibited limited cytotoxic activity against H1975 cells (Figure 5). These results suggest a compelling structure–activity relationship (SAR) involving the cyclopentanone core. In the present study, the weak inhibition effect of compound **9** suggested that the cleavage of the C-6-C-7 could decrease its cytotoxicity. Antitumor activity is enhanced by a hydroxy group at C-6 and C-15, which was supported by the fact that compound **2** was more effective than compound **5** [32]. Moreover, the antitumor activity is enhanced by the presence of a double bond at C-2 and C-3, as was demonstrated by the superior efficacy of compound **6** compared to compound **5**. The presence of substantial evidence suggests that modifying or altering its conformation, such as introducing a fluorine atom, can significantly enhance anti-HCC activity [47]. The compounds with the highest potency, or those exhibiting moderate efficacy possess multiple sites available for modification, and the development of their derivatives could potentially enhance anti-HCC activity.

### 2.3. Compound ***6*** Selectively Induced Apoptosis in Two Human Cancer Cell Lines

**Figure 6:**
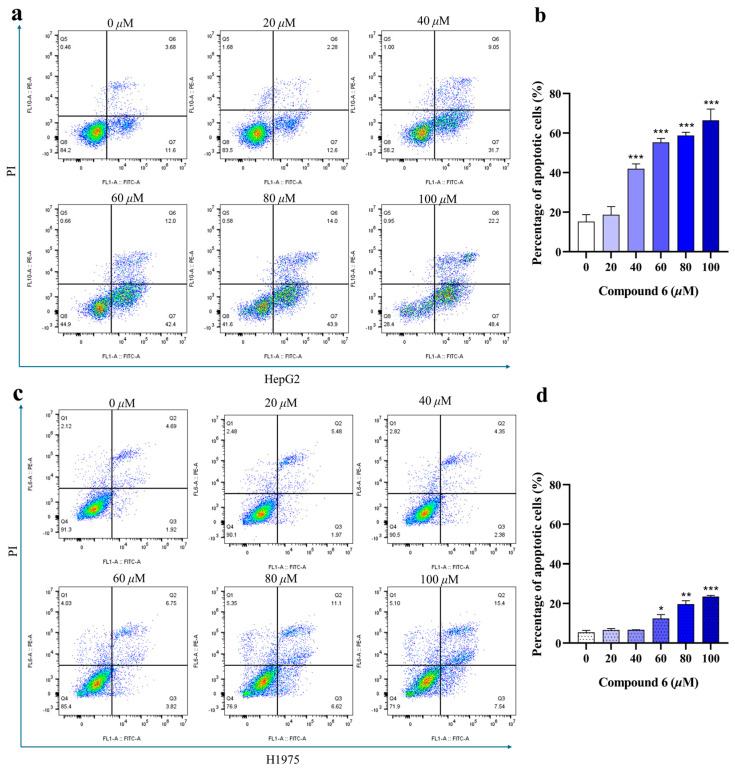
Flow cytometric analysis of apoptosis in HepG2 and H1975 cells (**a**,**c**). The apoptosis of HepG2 or H1975 cell lines was detected by flow cytometry based on PI staining after treatment with various concentrations of compound **6** (**b**,**d**). Histogram showing the proportions of apoptotic cells after 24 h of treatment with compound **6**. The results are expressed as mean ± SD. (* *p* < 0.05, ** *p* < 0.01, *** *p* < 0.001 versus the control group).

Apoptosis is essential for maintaining a delicate equilibrium between cell death and proliferation [48]. Evasion of apoptosis results in uncontrolled cell multiplication, thereby contributing to various diseases such as cancer [49]. The cytotoxic results have demonstrated that compound **6**, the primary compound in the PE part, exhibited the highest efficacy in suppressing the proliferation of HepG2 cells. PI staining was conducted to assess whether its cytotoxicity was caused by apoptosis. As shown in Figure 6a,b, HepG2 cells treated with compound **6** displayed a significant dose-dependent increase in the percentage of apoptotic cells compared to that in the control group. Meanwhile, compound **6** induced a higher level of apoptosis in HepG2 cells than in H1975 cells, indicating that the effect was not cell-type dependent (Figure 6c,d).

Targeting the apoptotic pathway presents a compelling strategy for the discovery of novel anti-HCC therapies, as evading apoptosis is a hallmark of cancer [50]. The present study conducted an unprecedented investigation into the cytotoxicity of compound 6 in inducing apoptotic cell death in HepG2 and H1975 cells. Moreover, this data indicated the potential of compound 6 as a promising targeted-anti-liver-cancer candidate by selectively inducing cell death in liver cancer cells.

## 3. Materials and Methods

### 3.1. General Experimental Procedures

Optical rotations were measured with a Rudolph Research Analytical Autopol I automatic polarimeter. Ultraviolet (UV) was recorded on a Yokechina double-beam ultraviolet-visible spectrophotometer. IR spectra analyses were performed on a Bruke TENSOR37 infrared spectrometer (KBr). HR-ESI-MS spectra analyses were performed on an Agilent 1290 Infinity LC and Agilent 6530 Q-TOF mass spectrometer (Agilent, Palo Alto, CA, USA). 1D and 2D NMR spectra were taken on a Bruker Ascend 600 NMR spectrometer with TMS as an internal reference. Column chromatography (CC) was performed on a silica gel (80–100 mesh, Huanghai, China) column. Ultra Performance Liquid Chromatography (UPLC) was performed on the Waters XSelect Premier HSS T3 column (100 mm × 2.0 mm, 2.2 μm). Medium-pressure liquid chromatography (MPLC) was performed on a RUIHE LC-2100 liquid chromatography instrument with an RP-18 column (YMC C_18_, 46 × 600 mm, 40–60 μm particle size). Semi-preparative HPLC was conducted on a RUIHE LC-2010 liquid chromatography instrument with a Waters Xbridge Prep C_18_ column (10 × 250 mm, 5 μm) or XSelect Prep C18 column (10 × 250 mm, 5 μm).

Ethanol, petroleum ether, and ethyl acetate were acquired from Ghtech (Guangzhou, China). The HPLC-grade solvents (methanol and acetonitrile) were obtained from Thermo Fisher Scientific (Suwanee, GA, USA). Deuterated chloroform (CDCl_3_) was purchased from Macklin (Shanghai, China). 3-(4,5-dimethylthiazol-2-yl)-2,5-diphenyltetrazolium bromide (MTT) was purchased from Solarbio (Beijing, China). Phosphate buffer saline (PBS), Dulbecco’s modified Eagle medium (DMEM), RPMI-1640, 0.25% Trypsin-Ethylene diamine tetraacetic acid (EDTA) solution, and penicillin-streptomycin solution were purchased from Servicebio (Wuhan, China). Fetal bovine serum (FBS) was obtained from Gibco (Waltham, MA, USA).

### 3.2. Plant Materials

The aerial parts of *I. serra* were collected from the Qingyuan City of Guangdong Province, People’s Republic of China, in October 2021 and identified by one of the authors, Dr. Ji Yang. The voucher specimen (IS-2021-10) was stored at the School of Traditional Chinese Materia Medica Guangdong Pharmaceutical University.

### 3.3. Extraction and Isolation

The air-dried aerial part of *I. serra* (80 kg) was extracted by reflux with 80% EtOH three times (320 L). After evaporation, the ethanol extract (EE) was partitioned sequentially with petroleum ether (PE), ethyl acetate (EA), and aqueous solution (AS). The partial PE fraction (300 g) was subjected to silica gel CC with PE:EtOAc:MeOH (100:0:0→0:100:0→0:0:100) to provide four selected fractions (Fr. A–D). Subsequently, Fr. A (7.29 g) was further separated by MPLC and eluted with a stepwise gradient elution of MeOH−H_2_O (15:85→100:0, *v*:*v*) to yield four subfractions, Fr.A1–A4. Then, Fr.A1 was isolated by MPLC and eluted with MeOH–H_2_O (45:55→100:0, *v*:*v*) to obtain two subfractions (Fr.A1-a and A1-b). Fr.A1-a was purified using semi-preparative HPLC to afford compounds **1** (1.26 mg) and **5** (103.2 mg) using a mobile phase of MeOH−H_2_O (40:60). Similarly, compounds **3** (6.39 mg) and **7** (1.33 mg) were isolated from Fr.A1-b via semi-preparative HPLC (MeOH−H_2_O, 65:35). Fr.A2 was separated with semi-preparative HPLC (MeOH−H_2_O, 55:45) to obtain compound **2** (5.91 mg). Following, Fr.A3 was subjected to an MPLC protocol using the eluent of MeOH−H_2_O (40:60→100:0, *v*:*v*) to provide Fr.A3-a and Fr.A3-b. Compound **8** (2.46 mg) was purified from Fr.A3-a by sim-PHPLC with a mobile phase of MeOH−H_2_O (58:42). Fr.A3-b was further fractionated using semi-preparative HPLC with a mobile phase of (MeOH−H_2_O, 75:25) to give compound **4** (1.35 mg). Compounds **6** (765.21 mg) and **9** (42.50 mg) were acquired using recrystallization from Fr. B and Fr. C, respectively. The residue of Fr. C (3.67 g) was fractionated using MPLC with a gradient of MeOH−H_2_O (15:85→100:0, *v*:*v*) to give two subfractions (Fr.C1 and Fr.C2). Then Fr.C1 was separated by MPLC eluted with MeOH−H_2_O (65:35, *v*:*v*), which was repurified by semi-preparative HPLC with a mobile phase of MeOH−H_2_O (54:46) to yield compounds **10** (3.76 mg) and **13** (2.67 mg). Fr.C2 was further purified by semi-preparative HPLC with a mobile phase of MeOH−H_2_O (60:40) to give compounds **11** (3.11 mg) and **12** (2.89 mg).

*Isodosin A* (**1**): White amorphous powder; [α${]}_{D}^{20.7}$ − 49.10 (c = 0.5, MeOH); UV (MeOH) *λ*_max_ (log ε) 219 (6.39), 274 (2.66); IR (KBr) *ν*_max_: 3435, 2927, 1736, 1579, 1373, 1245, 1054 cm^−1^; ^1^H and ^13^C NMR, see Table 1; HR-ESI-MS *m*/*z* 453.1888 [M + Na]^+^ (calculated for C_24_H_30_O_7_Na, 453.1884).

*Isodosin B* (**2**): White amorphous powder; [α${]}_{D}^{23.3}$ − 168.48 (c = 0.5, MeOH); UV (MeOH) *λ*_max_ (log ε) 212 (3.29), 296(1.95); IR (KBr) *ν*_max_: 3421, 2929, 1698, 1372, 1246, 1081cm^−1^; ^1^H and ^13^C NMR, see Table 1; HR-ESI-MS *m*/*z* 349.2011 [M + H]^+^ (calculated for C_20_H_29_O_5_, 349.2010).

*Isodosin C* (**3**): White amorphous powder; [α${]}_{D}^{21.5}$ + 75.53 (c = 0.5, MeOH); UV (MeOH) *λ*_max_ (log ε) 208 (3.03), 219 (2.58), 310 (2.04); IR (KBr) *ν*_max_: 2918, 1734, 1373, 1228, 1055 cm^−1^; ^1^H and ^13^C NMR, see Table 1; HR-ESI-MS *m*/*z* 433.2224 [M + H]^+^ (calculated for C_24_H_33_O_7_, 433.2221).

*Isodosin D* (**4**): White amorphous powder; [α${]}_{D}^{21.3}$ − 19.41 (c = 0.3, MeOH); UV (MeOH) *λ*_max_ (log ε) 210 (3.02), 235 (2.52), 300 (1.96); IR (KBr) *ν*_max_: 3392, 2929, 2360, 1594, 1456, 1260, 1029, 800 cm^−1^; ^1^H and ^13^C NMR, see Table 1; HR-ESI-MS *m*/*z* 305.2471 [M + H]^+^ (calculated for C_20_H_33_O_2_, 305.2475).

### 3.4. ECD Calculations

The conformational analyses were performed using random searching in Spartan 16 (Wavenfunction, Irvine, CA, USA, 2016) employing the MMFF94 force field. Subsequently, the conformers were re-optimized using DFT at the B3LYP/6-31G(d,p) level and utilizing the polarizable conductor calculation model (SMD) provided by the Gaussian 09 program. The energies, oscillator strengths, and rotational strengths (velocity) of conformers with a Boltzmann distribution greater than 1% were calculated using TDDFT methodology at the B3LYP/6-311+g(d,p) level in MeOH. The ECD spectra were simulated by convolving Gaussian functions (half bandwidth at 1/e peak height, sigma ¼ 0.30 for all). The final spectra were obtained by averaging the simulated spectra of each conformer based on Boltzmann distribution theory and their relative Gibbs free energy (Δ_G_) values.

### 3.5. Cell Lines and Cell Culture

Human hepatocellular carcinoma HepG2 and H1975 cell lines were obtained from the American Type Culture Collection (ATCC, Manassas, VA, USA). HepG2 cells were cultured in DMEM medium supplemented with 10% FBS and 1% antibiotics (penicillin and streptomycin) in an incubator containing 5% CO_2_ at 37 °C. H1975 cells were grown in RPMI 1640 medium containing 10% (*v*/*v*) FBS and 1% antibiotics in a cell incubator with 5% CO_2_ at 37 °C.

### 3.6. Cytotoxicity Assay

Cytotoxicities of the main compounds (**2**, **3**, **5**, **6**, and **9**) against HepG2 and H1975 cell lines were evaluated with the MTT assay as described in the literature [32]. Briefly, HepG2 or H1975 cells (5 × 10^3^ cells/well) were cultured in 96-well plates. After seeding and incubating for 24 h, the cells were treated with the compounds at various concentrations (0, 25, 50, and 100 μM) for 24 h. After this treatment, MTT solution was added and the cells were incubated for 4 h at 37 °C in an atmosphere of 5% CO_2_. DMSO was added to dissolve the formazan after the supernatant medium was removed. Finally, absorbance was detected at 570 nm using a Multiskan SkyHigh Spectrum (Thermo Scientific™, Waltham, MA, USA).

### 3.7. Analysis of Apoptosis by PI Staining

HepG2 and H1975 cells (1.5 × 10^5^ cells/well) were plated in 6-well plates and treated with compound **6** at 0, 20, 40, 60, 80, or 100 μM for 24 h. The cells were subsequently collected, re-suspended in 70% ethanol, and fixed at 4 °C for 30 min. Cells were centrifuged at 1000 rpm for 5 min to separate the supernatant. Each cell pellet was stained in 300 μL propidium iodide (PI) (50 μg/mL) staining solution at 37 °C for 30 min in the dark. Finally, the cells were washed twice with PBS and measured using flow cytometry (Beckman Coulter, CytoFLEX, Indianapolis, IN, USA).

### 3.8. Statistical Analysis

All values presented in the figures, tables, and text are presented as means ± SD. Statistical analysis was performed with a one-way analysis of variance (ANOVA) test using GraphPad Prism, Version 8.2.1 (GraphPad Software, Inc., San Diego, CA, USA). *p* < 0.05 was considered statistically significant.

## 4. Conclusions

In conclusion, a total of 13 diterpenoids (**1**–**13**) were isolated from the aerial parts of *I*. *serra*. Among them, compounds **1**–**3** were identified as new *ent*-kaurane diterpenoids, and compound **4** was identified as a new *ent*-abietane diterpenoid. Compounds **2**, **3**, and **6** demonstrate high selectivity towards HepG2 cells over H1975 cells. Moreover, compound **6** demonstrated a significant induction of apoptotic cell death specifically in HepG2 cells compared to H1975 cells. Our results suggest that compound **6** might be a highly promising lead candidate for targeted anti-liver cancer therapy by inducing apoptotic cell death. Overall, the findings of our study have significantly expanded the structural diversity of secondary metabolites derived from *I*. *serra* and provided evidence for the potential use of these diterpenoids in HCC treatment.

## Figures and Tables

**Table 1:** ^1^H NMR (600 MHz) and ^13^C NMR (150 MHz) spectroscopic data of compounds **1**–**4** in CDCl_3_ (*δ* in ppm, *J* in Hz).

Position	1		2		3		4	
	*δ*_C_ (Type)	*δ*_H_ (mult., *J* in Hz)	*δ*_C_ (Type)	*δ*_H_ (mult., *J* in Hz)	*δ*_C_ (Type)	*δ*_H_ (mult., *J* in Hz)	*δ*_C_ (Type)	*δ*_H_ (mult., *J* in Hz)
1a	211.0 (C)		213.4 (C)		30.3 (CH_2_)	1.49 (m)	37.9 (CH_2_)	1.17 (m)
1b						1.35 (m)		1.86 (m)
2a	36.3 (CH_2_)	2.47 (m)	35.5 (CH_2_)	2.68 (m)	18.1 (CH_2_)	1.53 (m)	27.4 (CH_2_)	1.62 (m)
2b		2.58 (m)		2.28 (m)		1.44 (m)		1.58 (overlap)
3a	38.4(CH_2_)	1.90 (m)	38.1 (CH_2_)	1.94 (m)	41.4 (CH_2_)	1.52 (m)	79.3 (CH)	3.26 (dd, 11.3, 4.0)
3b		1.78 (m)		1.72 (m)		1.24 (m)		
4	33.0 (C)		32.6 (C)	2.02 (m)	34.4 (C)		38.6 (C)	
5	57.0 (CH)	2.32 (d, 7.1)	56.1 (CH)	2.34 (d, 7.8)	61.9 (CH)	2.18 (s)	49.9 (CH)	1.13 (m)
6a	74.6 (CH)	5.14 (d, 7.1)	73.7 (CH)	3.84 (d, 7.8)	206.7 (C)		23.0 (CH_2_)	1.96 (m)
6b								1.94 (m)
7	97.0 (C)		96.8 (C)		92.6 (C)		120.4 (CH)	5.41 (s)
8	51.7 (C)		51.9 (C)		48.2 (C)		136.8 (C)	
9	44.1 (CH)	3.05 (m)	41.0 (CH)	2.40 (m)	47.0 (CH)	2.00 (d, 3.4)	52.3 (CH)	1.68 (m)
10	50.1 (C)		48.7 (C)		38.7 (C)		35.3 (C)	
11a	124.3 (CH)	5.58(dd, 9.4, 2.4)	17.2 (CH_2_)	1.68 (m)	68.0 (CH)	5.30 (t, 5.1)	25.9 (CH_2_)	1.78 (m)
11b				1.04 (m)				1.15 (m)
12a	138.0 (CH)	6.37(m)	32.2 (CH_2_)	2.17 (m)	42.0 (CH_2_)	2.21 (m)	31.6 (CH_2_)	1.87 (m)
12b				1.44 (m)		1.85 (m)		1.22 (m)
13	38.4 (CH)	3.02 (m)	35.4 (CH)	2.68 (m)	35.6 (CH)	2.81 (m)	41.2 (CH)	1.89 (m)
14a	33.2 (CH_2_)	2.14 (dd, 11.7, 4.6)	25.2 (CH_2_)	1.80 (d, 11.9)	25.0 (CH_2_)	2.40 (d, 12.3)	40.8 (CH_2_)	2.33 (m)
14b		1.64 (overlap)		1.50 (m)		1.85 (m)		2.33 (m)
15	74.3 (CH)	5.90 (t, 2.1)	74.2 (CH)	4.42 (m)	76.6 (CH)	5.50 (m)	153.6 (C)	
16a	153.0 (C)		159.0 (C)		154.1 (C)		65.2 (CH_2_)	4.12 (s)
16b								4.12 (s)
17a	109.2 (CH_2_)	5.00 (d, 2.0)	108.9 (CH_2_)	5.18 (d, 2.1)	110.0 (CH_2_)	5.07 (m)	107.8 (CH_2_)	5.03 (m)
17b		4.86 (brs)				4.86 (brs)		4.88 (s)
18	29.7 (CH_3_)	0.92 (s)	30.3 (CH_3_)	1.12 (s)	35.0 (CH_3_)	1.37 (s)	28.4 (CH_3_)	1.00 (s)
19	22.7 (CH_3_)	1.10 (s)	23.9 (CH_3_)	0.98 (s)	22.0 (CH_3_)	1.05 (s)	15.6 (CH_3_)	0.89(s)
20a	66.0 (CH_2_)	4.10 (d, 9.9)	64.9 (CH_2_)	4.37 (d, 10.2)	68.0 (CH_2_)	4.50 (d, 9.4)	15.0 (CH_3_)	0.81 (s)
20b		3.98 (dd, 9.9, 3.0)		3.96 (dd, 10.2, 1.6)		4.15 (d, 9.4)		
OAc-6	170.3 (C)							
	21.9 (CH_3_)	2.08 (s)						
OAc-15	173.8 (C)				169.6 (C)			
	21.0 (CH_3_)	2.20 (s)			20.6 (CH_3_)	2.02 (s)		
OAc-11					169.7 (C)			
					21.9 (CH_3_)	2.11 (s)		

## Data Availability

Data are contained within the article and Supplementary Materials.

## Supplementary Materials

The following supporting information can be downloaded at https://www.mdpi.com/article/10.3390/molecules29122733/s1. Physical and spectral data of **1**–**13** with citation.
