# Supplementary material for: Diterpenoids from the Aerial Parts of Isodon serra with Selective Cytotoxic Activity
Source: Molecules. 2024 Jun 8;29(12):2733. doi: 10.3390/molecules29122733 (PMC11207078; doi:10.3390/molecules29122733)
Supplement: Supplementary file 1 [file molecules-29-02733-s001.zip › molecules-3030361-supplementary.pdf]

Supplementary Materials

Diterpenoids from the aerial parts of *Isodon serra* with selective cytotoxic Activity

Siqin Li [1], Fang Liang [1], Dongdong Huang [1], Huanling Wu [1], Xiaohua Tan [1], Jiang Ma [1], CaiHong Wei [1], ShiXiong Wang [1], Ziying Huang [1], Guang Yang [2], Xin He[1,*], and Ji Yang [1,*]

[1] School of Traditional Chinese Materia Medica, Guangdong Pharmaceutical University, Guangzhou 510006, China; lisiqin_email@163.com (S.L.); liangfang_email@163.com (F.L.); hdongdong@163.com (D.H.); wuhuanling980@163.com (H.W.); ycyjzhongxin@163.com (X.T.); majiang@gdpu.edu.cn (J.M.); weicaihong1212@163.com (C.W.); HNWangshixiong@163.com (S.W.); ziying_huang2024@163.com (Z.H);

[2] China Academy of Chinese·Medical Sciences, Beijing 100700, China; hbykdxyg2008@163.com (G.Y.)

* Correspondence: hexintn@163.com (X.H.); yangji@gdpu.edu.cn (J.Y.); Tel.: +86-02039352179 (J.Y.)

Fig. S1. HR-ESI-MS spectrum of compound 1 5
Fig. S2. ^{1}H NMR spectrum for compound 1 5
Fig. S3. ^{13}C and DEPT135 NMR spectra for compound 1 6
Fig. S4. HSQC NMR spectrum for compound 1 6
Fig. S5. ^{1}H–^{1}H COSY NMR spectrum for compound 1 7
Fig. S6. HMBC NMR spectrum for compound 1 7
Fig. S7. NOESY NMR spectrum for compound 1 8
Fig. S8. IR spectrum of compound 1 (KBr) 8
Fig. S9. UV spectrum of compound 1 in MeOH 9
Fig. S10. CD spectrum of compound 1 in MeOH 9
Fig. S11. HR-ESI-MS spectrum of compound 2 10
Fig. S12. ^{1}H NMR spectrum for compound 2 10
Fig. S13. ^{13}C and DEPT135 NMR spectra for compound 2 11
Fig. S14. HSQC NMR spectrum for compound 2 11
Fig. S15. ^{1}H–^{1}H COSY NMR spectrum for compound 2 12
Fig. S16. HMBC NMR spectrum for compound 2 12
Fig. S17. NOESY NMR spectrum for compound 2 13
Fig. S18. IR spectrum of compound 2 (KBr) 13
Fig. S19. UV spectrum of compound 2 in MeOH 14
Fig. S20. CD spectrum of compound 2 in MeOH 14
Fig. S21. HR-ESI-MS spectrum of compound 3 15
Fig. S22. ^{1}H NMR spectrum for compound 3 15
Fig. S23. ^{13}C and DEPT135 NMR spectra for compound 3 16
Fig. S24. HSQC NMR spectrum for compound 3 16
Fig. S25. ^{1}H–^{1}H COSY NMR spectrum for compound 3 17
Fig. S26. HMBC NMR spectrum for compound 3 17
Fig. S27. NOESY NMR spectrum for compound 3 18
Fig. S28. IR spectrum of compound 3 (KBr) 18
Fig. S29. UV spectrum of compound 3 in MeOH 19
Fig. S30. CD spectrum of compound 3 in MeOH 19
Fig. S31. HR-ESI-MS spectrum of compound 4 20

Fig. S32. ^{1}H NMR spectrum for compound 4 20
Fig. S33. ^{13}C and DEPT135 NMR spectra for compound 4 21
Fig. S34. HSQC NMR spectrum for compound 4 21
Fig. S35. ^{1}H–^{1}H COSY NMR spectrum for compound 4 22
Fig. S36. HMBC NMR spectrum for compound 4 22
Fig. S37. NOESY NMR spectrum for compound 4 23
Fig. S38. IR spectrum of compound 4 (KBr) 23
Fig. S39. UV spectrum of compound 4 in MeOH 24
Fig. S40. CD spectrum of compound 4 in MeOH 24
Fig. S41. ^{1}H NMR spectrum for compound 5 25
Fig. S42. ^{13}C and DEPT135 NMR spectra for compound 5 25
Fig. S43. ^{1}H NMR spectrum for compound 6 26
Fig. S44. ^{13}C and DEPT135 NMR spectra for compound 6 26
Fig. S45. ^{1}H NMR spectrum for compound 7 27
Fig. S46. ^{13}C and DEPT135 NMR spectra for compound 7 27
Fig. S47. ^{1}H NMR spectrum for compound 8 28
Fig. S48. ^{13}C and DEPT135 NMR spectra for compound 8 28
Fig. S49. ^{1}H NMR spectrum for compound 9 29
Fig. S50. ^{13}C and DEPT135 NMR spectra for compound 9 29
Fig. S51. ^{1}H NMR spectrum for compound 10 30
Fig. S52. ^{13}C and DEPT135 NMR spectra for compound 10 30
Fig. S53. ^{1}H NMR spectrum for compound 11 31
Fig. S54. ^{13}C and DEPT135 NMR spectra for compound 11 31
Fig. S55. ^{1}H NMR spectrum for compound 12 32
Fig. S56. ^{13}C and DEPT135 NMR spectra for compound 12 32
Fig. S57. ^{1}H NMR spectrum for compound 13 33
Fig. S58. ^{13}C and DEPT135 NMR spectra for compound 13 33
Table S1. Key conformers of compound 1........ 34
Table S2. Conformers and Bolzmann distributions of the optimized 1. 34
Table S3. Optimized Z-matrixes of isomer 1 in the gas phase (Å) at B3LYP/6-31G(d,p) level. 34
Table S4. Key conformers of compound 2........ 37

Table S5. Conformers and Bolzmann distributions of the optimized 2 37

Table S6. Optimized Z-matrixes of isomer 2 in the gas phase (Å) at B3LYP/6-31G(d,p) level. 37

Table S7. Key conformers of compound 3. 39

Table S8. Conformers and Bolzmann distributions of the optimized 3. 39

Table S9. Optimized Z-matrixes of isomer 3 in the gas phase (Å) at B3LYP/6-31G(d,p) level. 39

Table S10. Key conformers of compound 4. 41

Table S11. Conformers and Bolzmann distributions of the optimized 4................................. 41

Table S12. Optimized Z-matrixes of isomer 4 in the gas phase (Å) at B3LYP/6-31G(d,p) level. 41

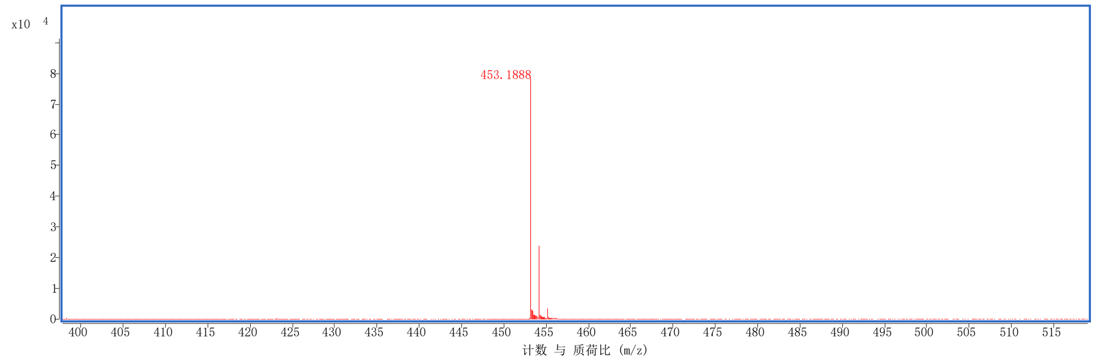

Formula (M)	Score (MFG)	Mass	Mass (MFG)	*m/z* (Calc)	Diff (ppm)	*m/z*
$C_{24}H_{30}O_7$	100	430.1996	430.1992	453.1884	-0.06	453.1888

Fig. S1. HR-ESI-MS spectrum of compound **1**

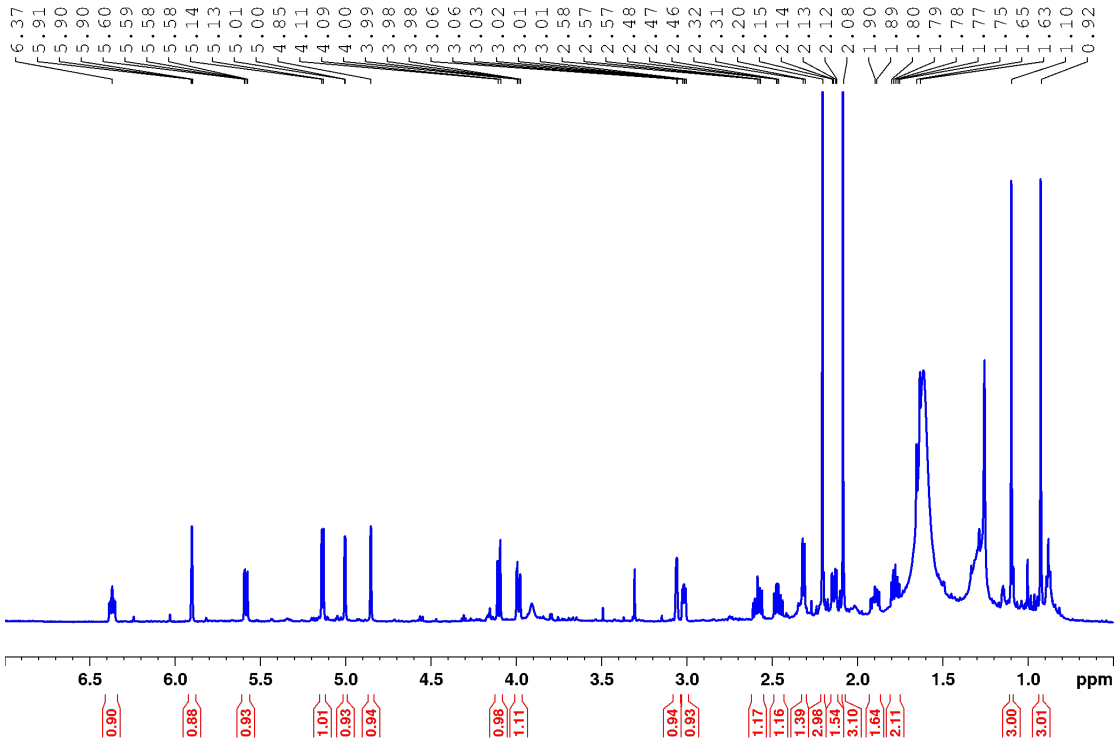

Fig. S2. ^{1}H NMR spectrum for compound **1**

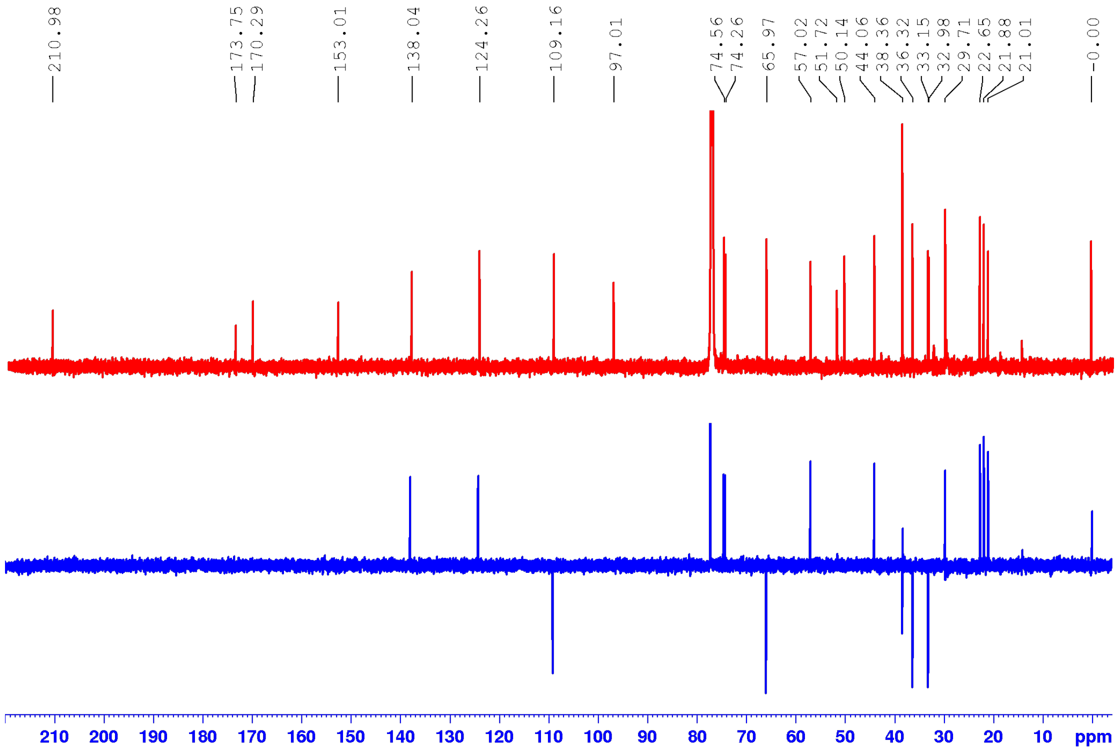

Fig. S3. ^{13}C and DEPT135 NMR spectra for compound **1**

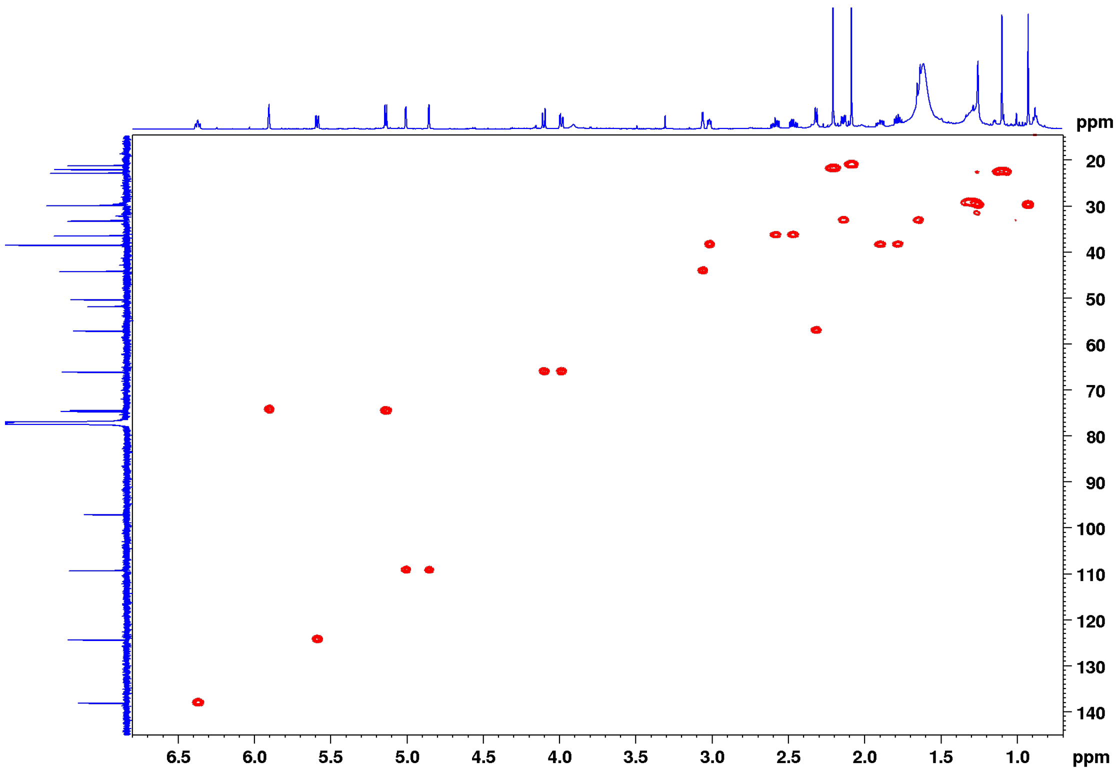

Fig. S4. HSQC NMR spectrum for compound **1**

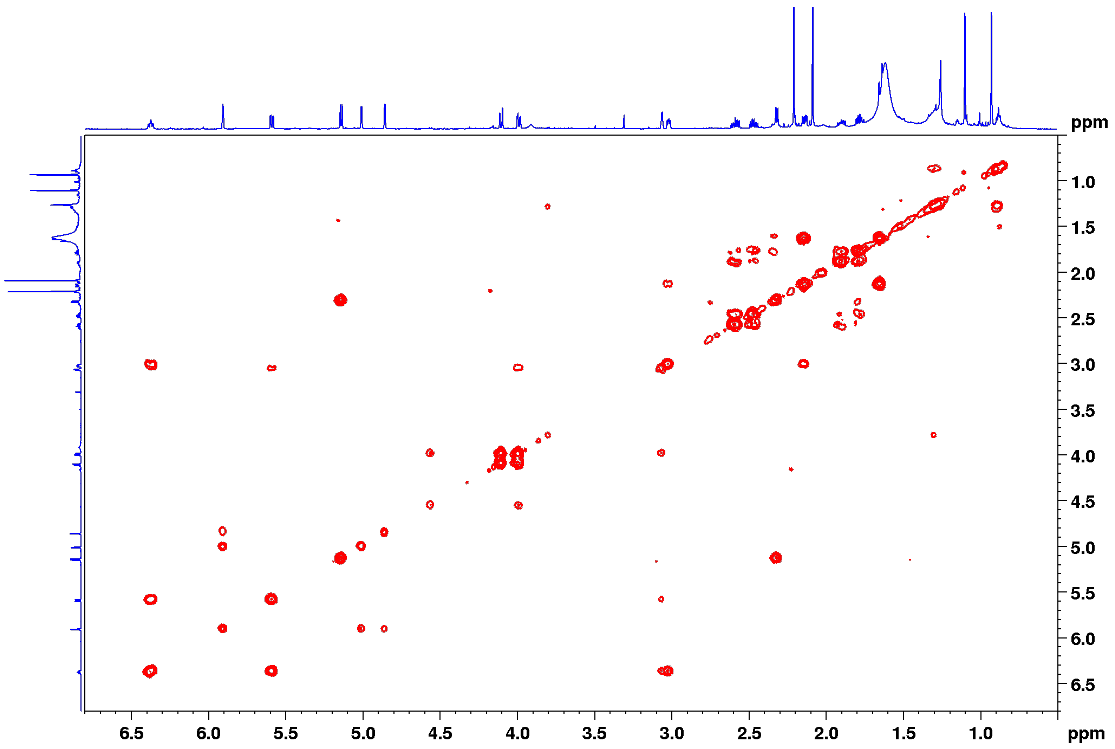

Fig. S5. ¹H–¹H COSY NMR spectrum for compound **1**

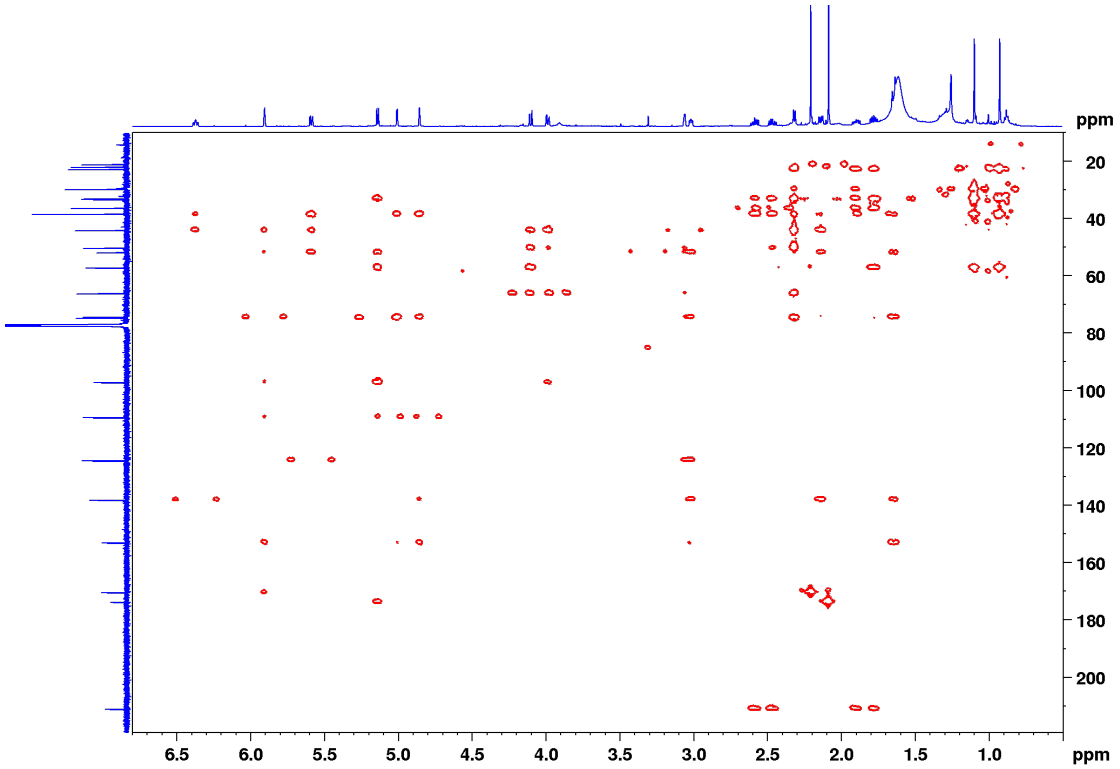

Fig. S6. HMBC NMR spectrum for compound **1**

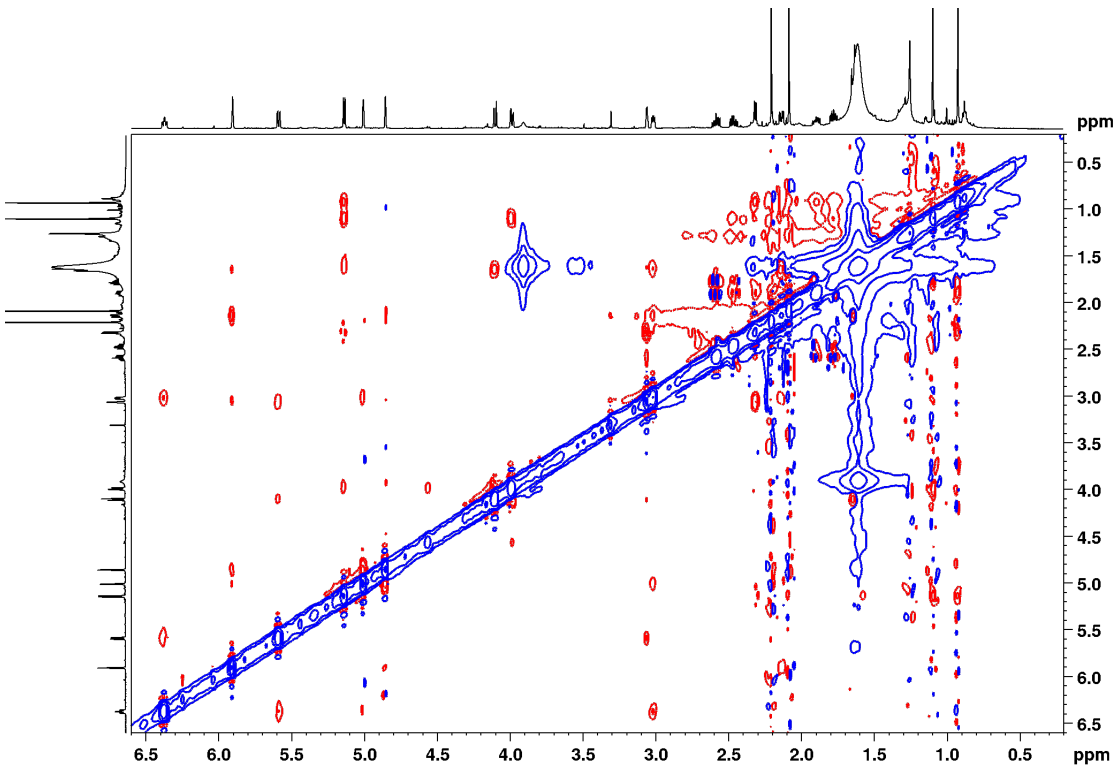

Fig. S7. NOESY NMR spectrum for compound **1**

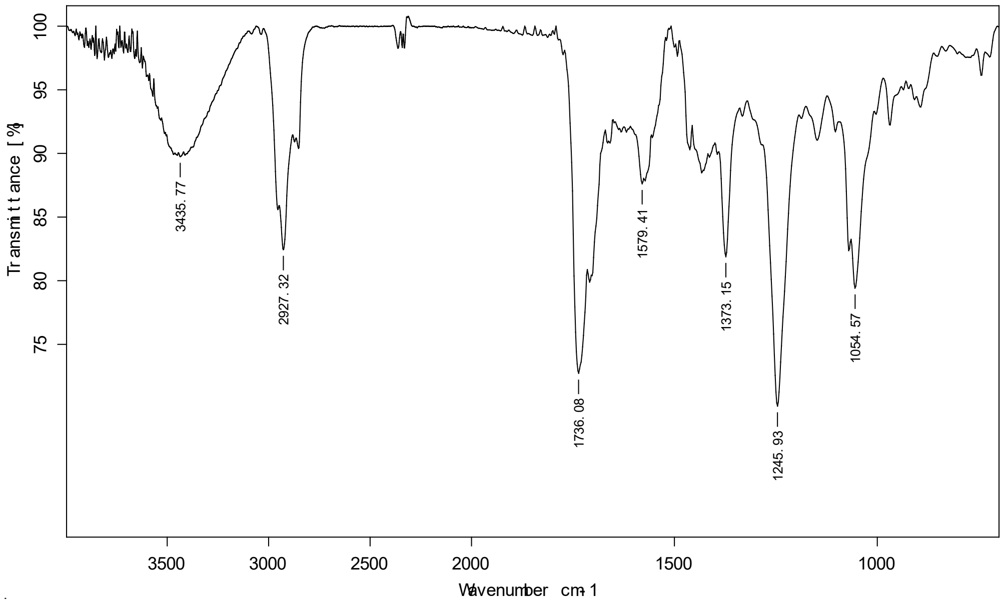

Fig. S8. IR spectrum of compound **1** (KBr)

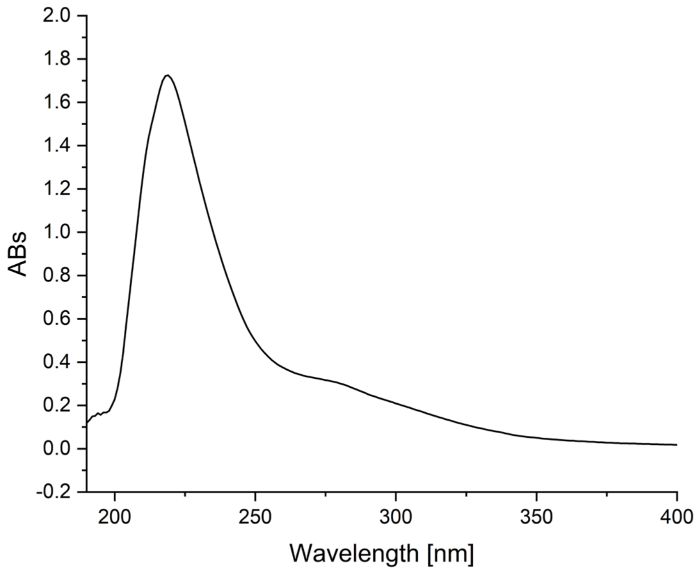

Fig. S9. UV spectrum of compound **1** in MeOH

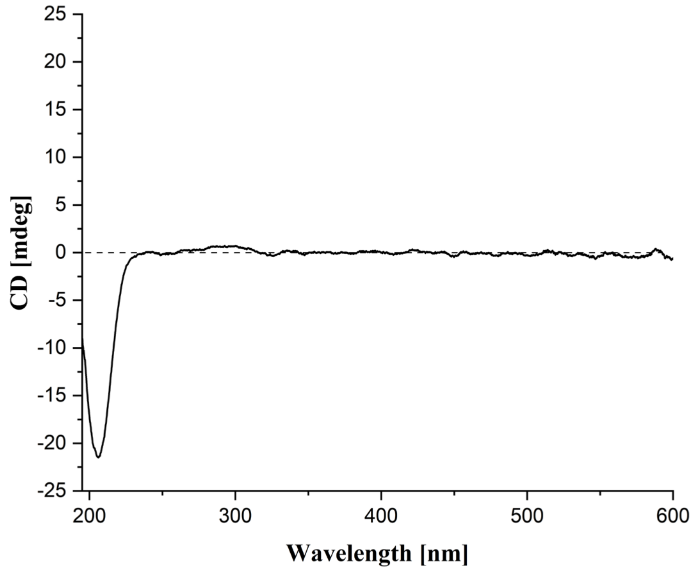

Fig. S10. CD spectrum of compound **1** in MeOH

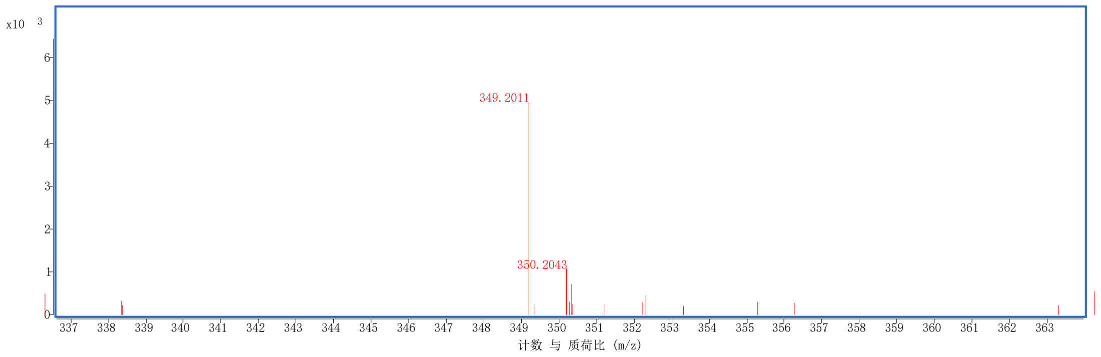

Formula (M)	Score (MFG)	Mass	Mass (MFG)	*m/z* (Calc)	Diff (ppm)	*m/z*
$C_{20}H_{28}O_5$	99.93	348.1938	348.1937	349.2010	0.43	349.2011

Fig. S11. HR-ESI-MS spectrum of compound **2**

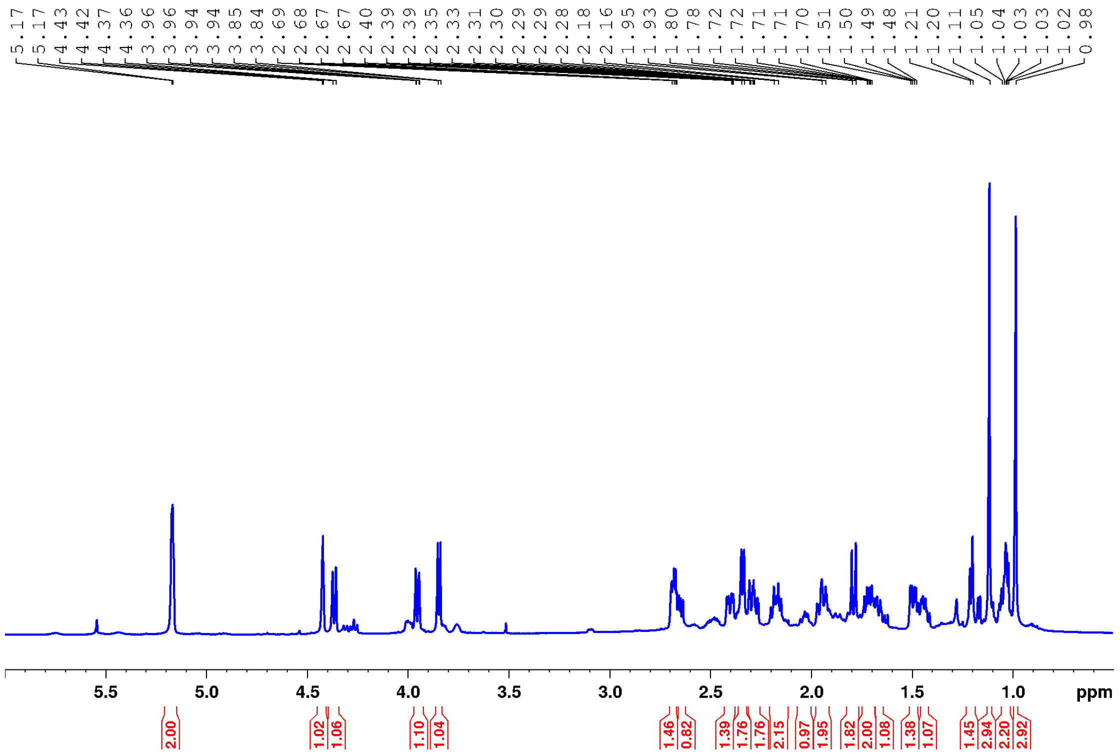

Fig. S12. ^{1}H NMR spectrum for compound **2**

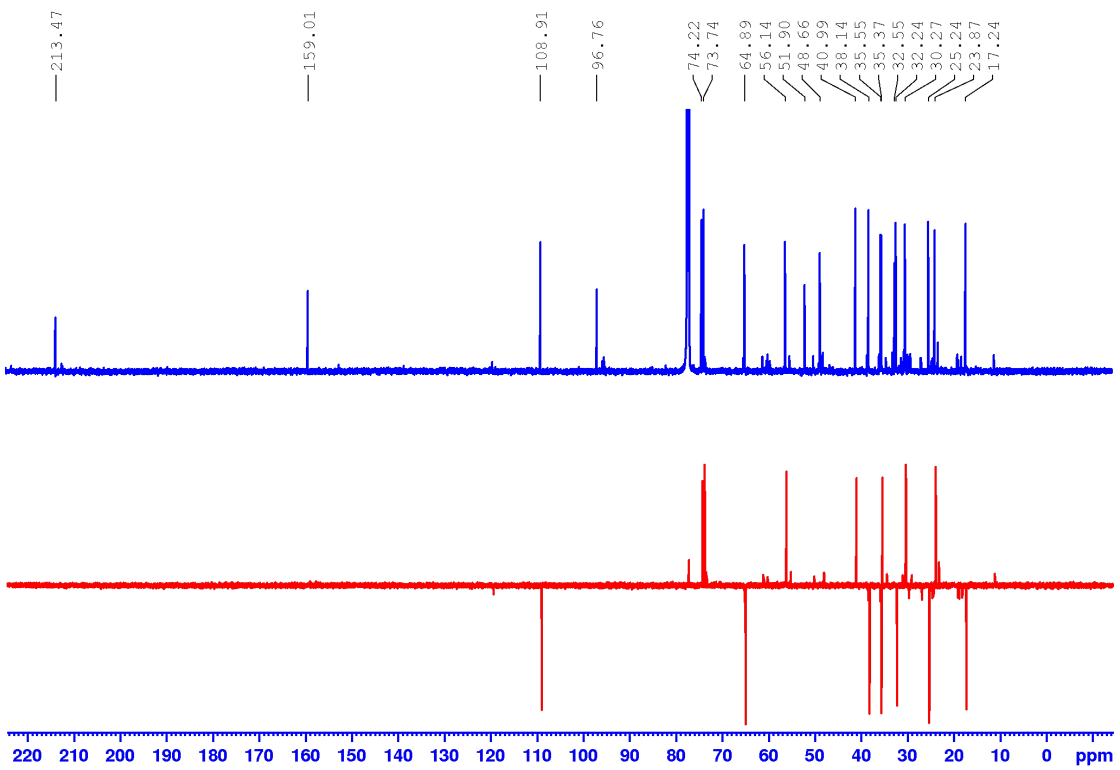

Fig. S13. ^{13}C and DEPT135 NMR spectra for compound **2**

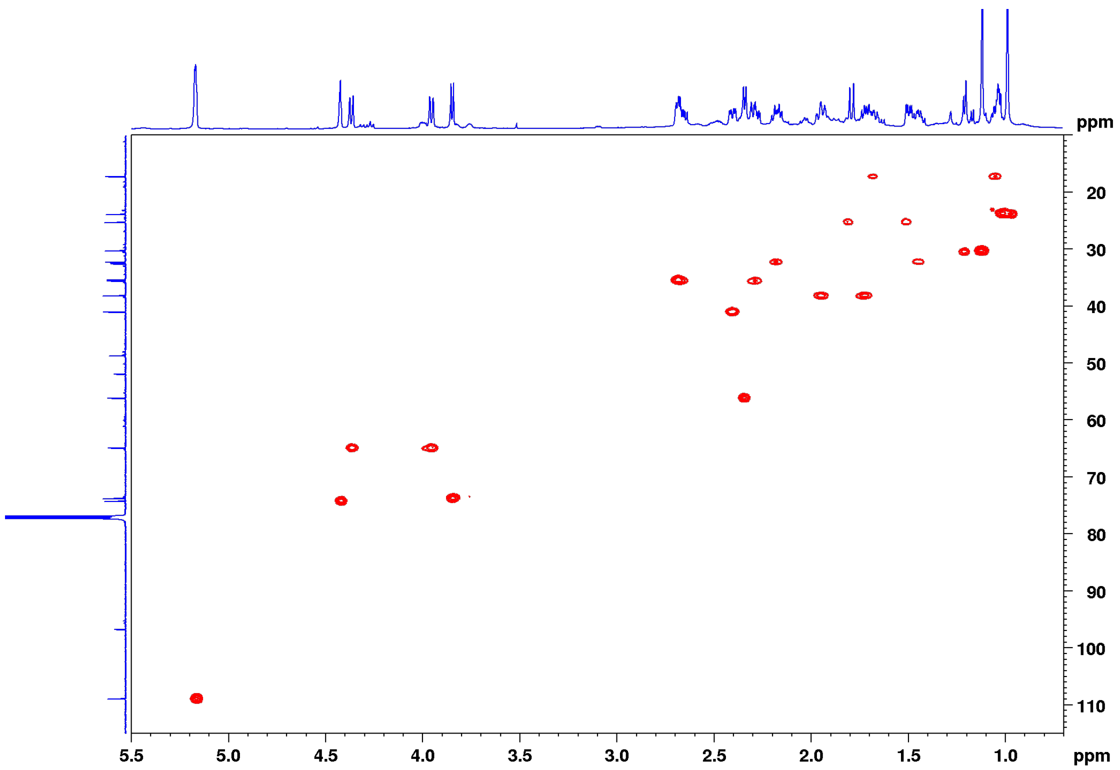

Fig. S14. HSQC NMR spectrum for compound **2**

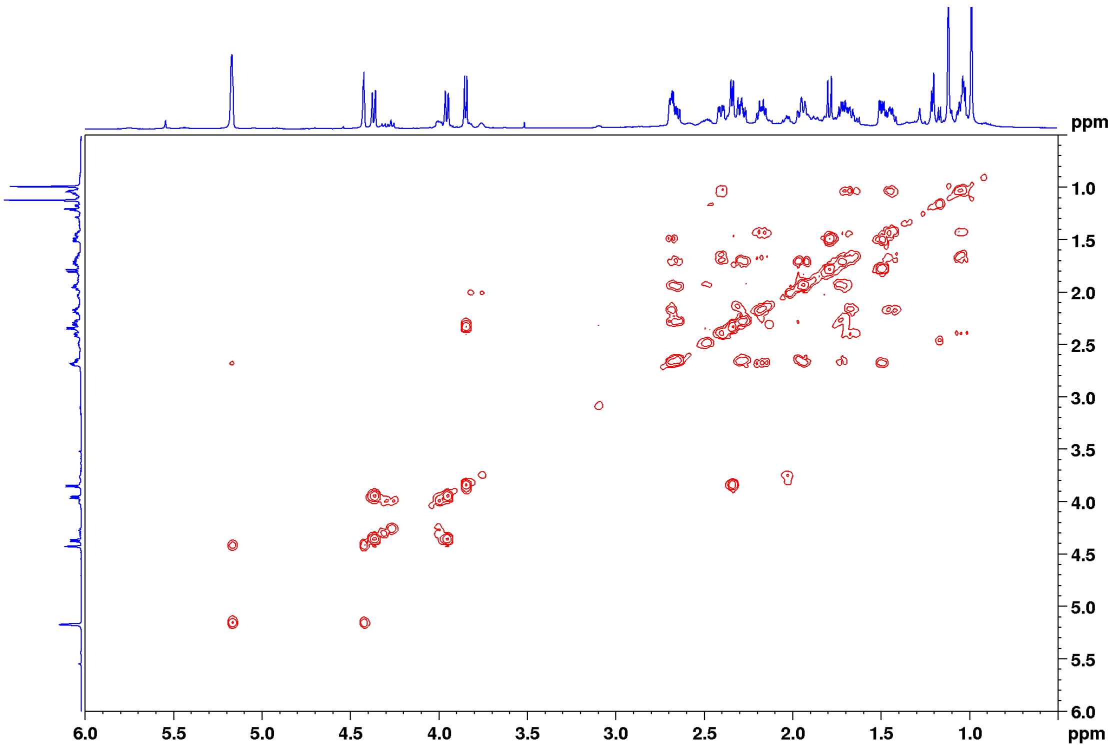

Fig. S15. ^{1}H–^{1}H COSY NMR spectrum for compound **2**

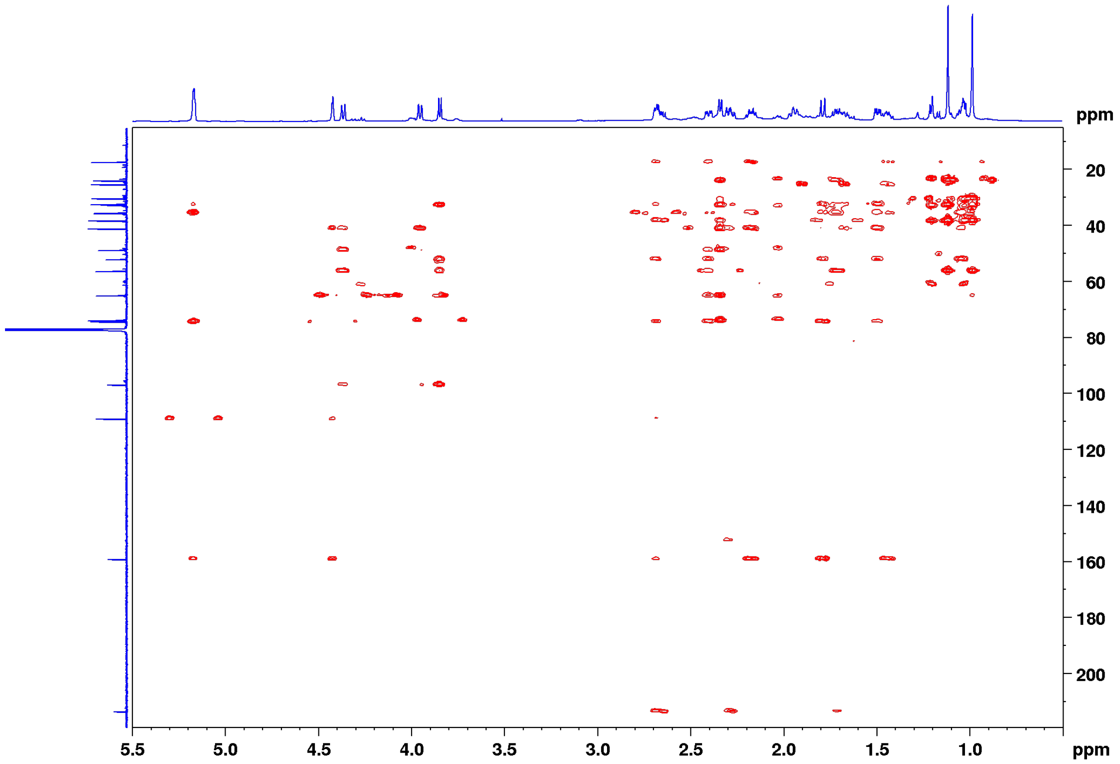

Fig. S16. HMBC NMR spectrum for compound **2**

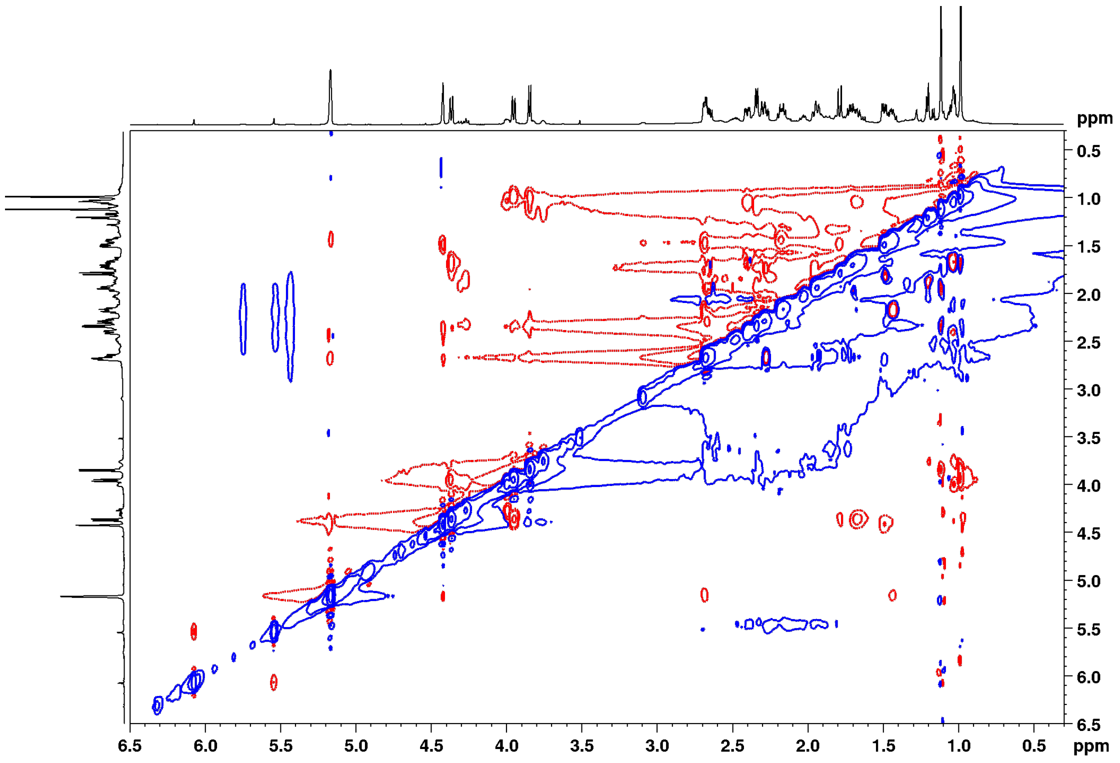

Fig. S17. NOESY NMR spectrum for compound **2**

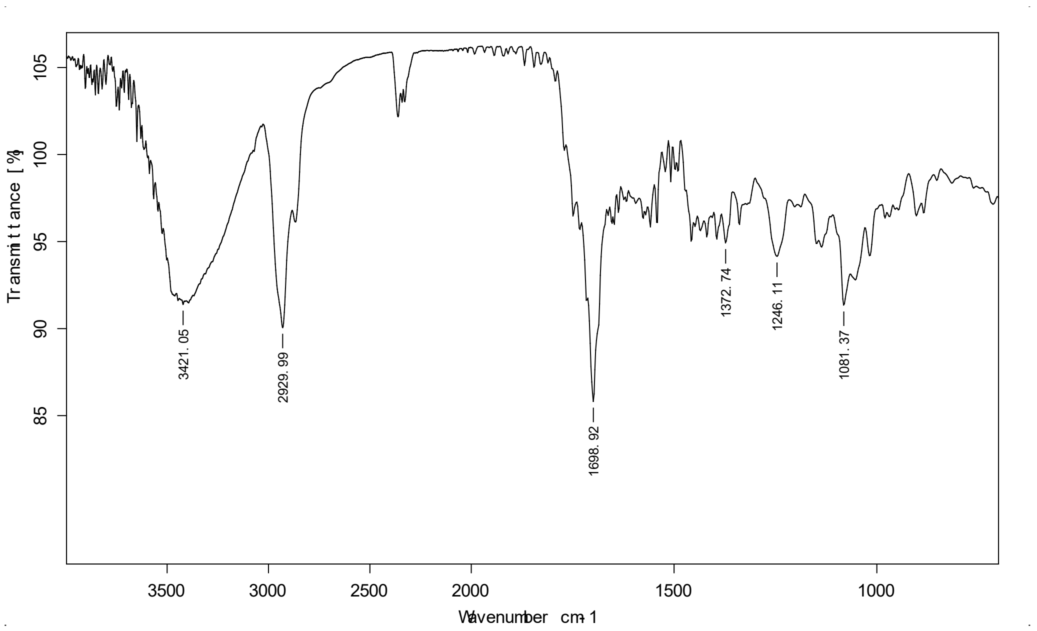

Fig. S18. IR spectrum of compound **2** (KBr)

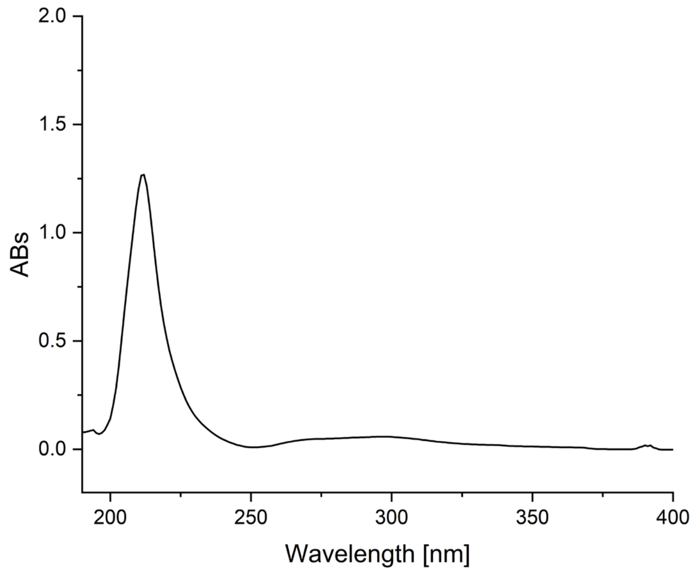

Fig. S19. UV spectrum of compound **2** in MeOH

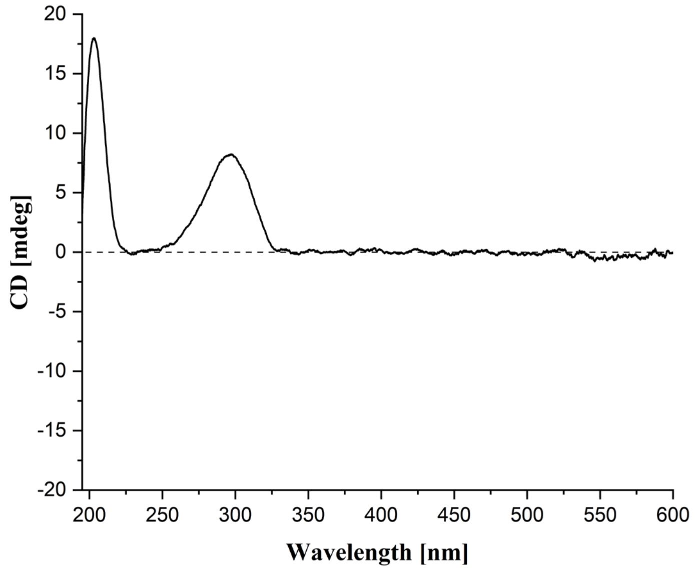

Fig. S20. CD spectrum of compound **2** in MeOH

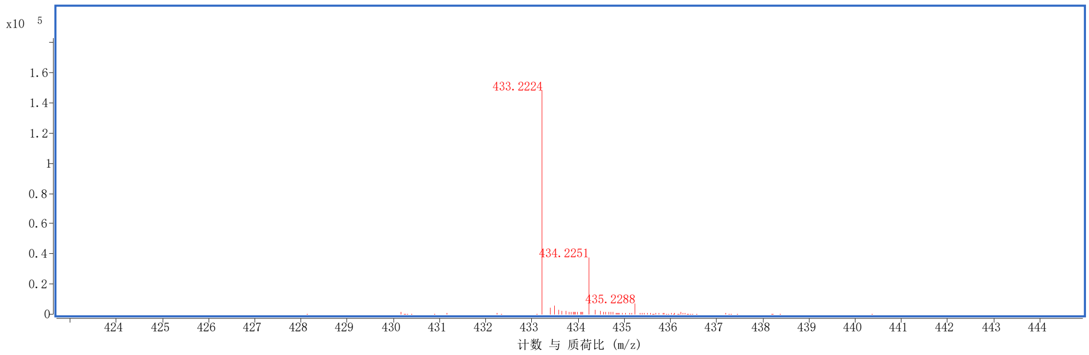

Formula (M)	Score (MFG)	Mass	Mass (MFG)	*m/z* (Calc)	Diff (ppm)	*m/z*
$C_{24}H_{32}O_7$	99.75	432.2151	432.2148	433.2221	-0.74	433.2224

Fig. S21. HR-ESI-MS spectrum of compound **3**

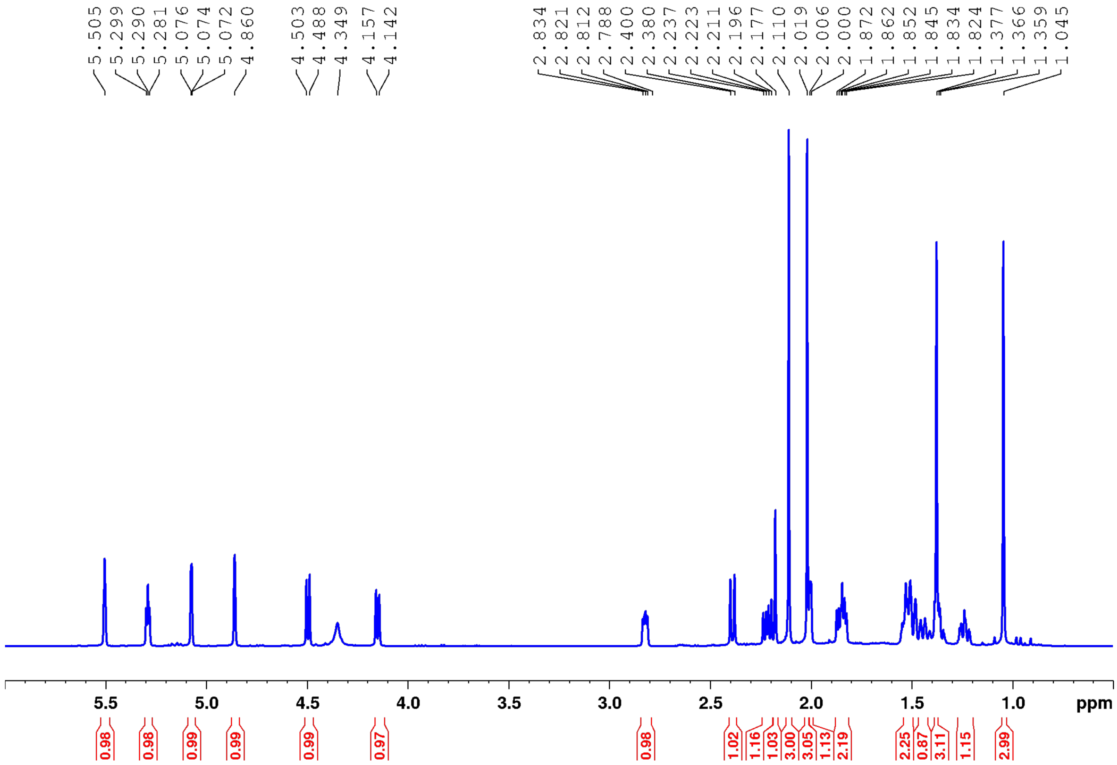

Fig. S22. ^{1}H NMR spectrum for compound **3**

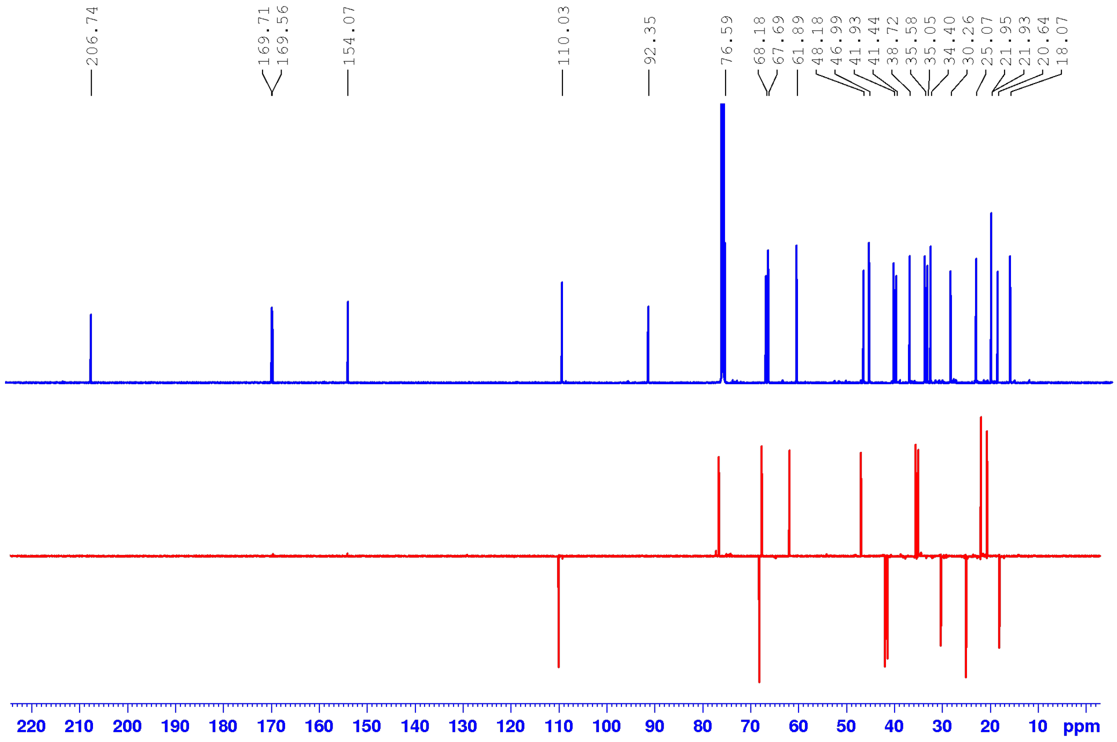

Fig. S23. ^{13}C and DEPT135 NMR spectra for compound **3**

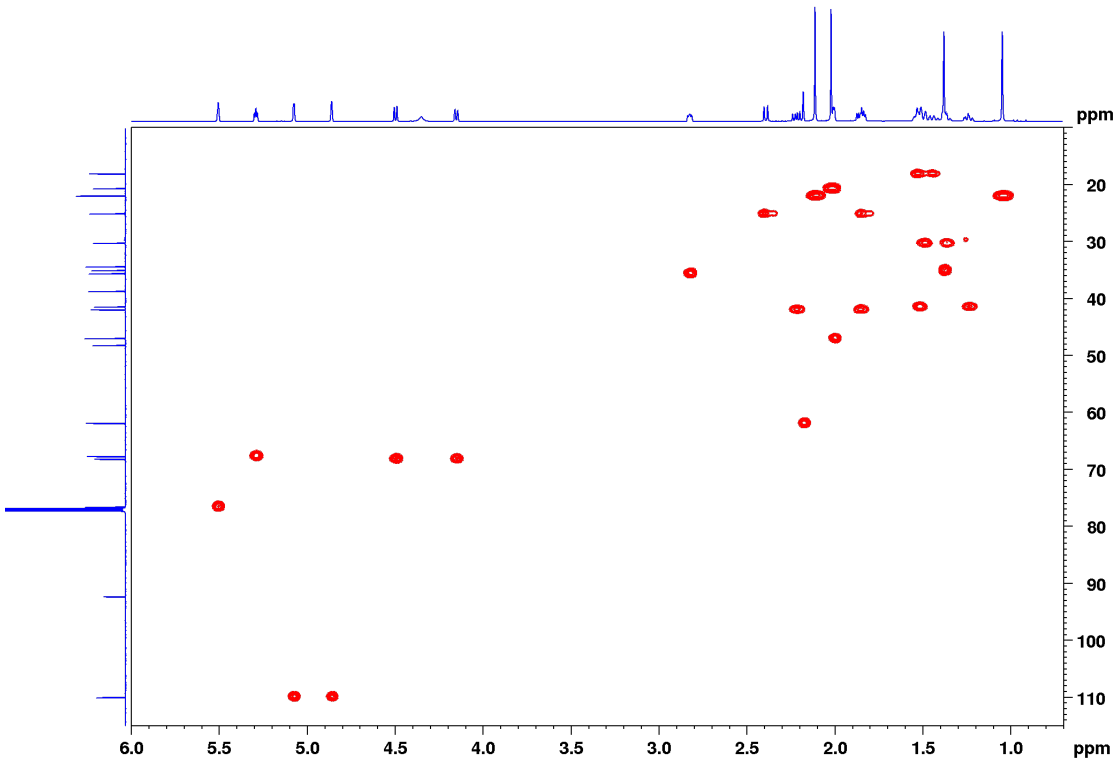

Fig. S24. HSQC NMR spectrum for compound **3**

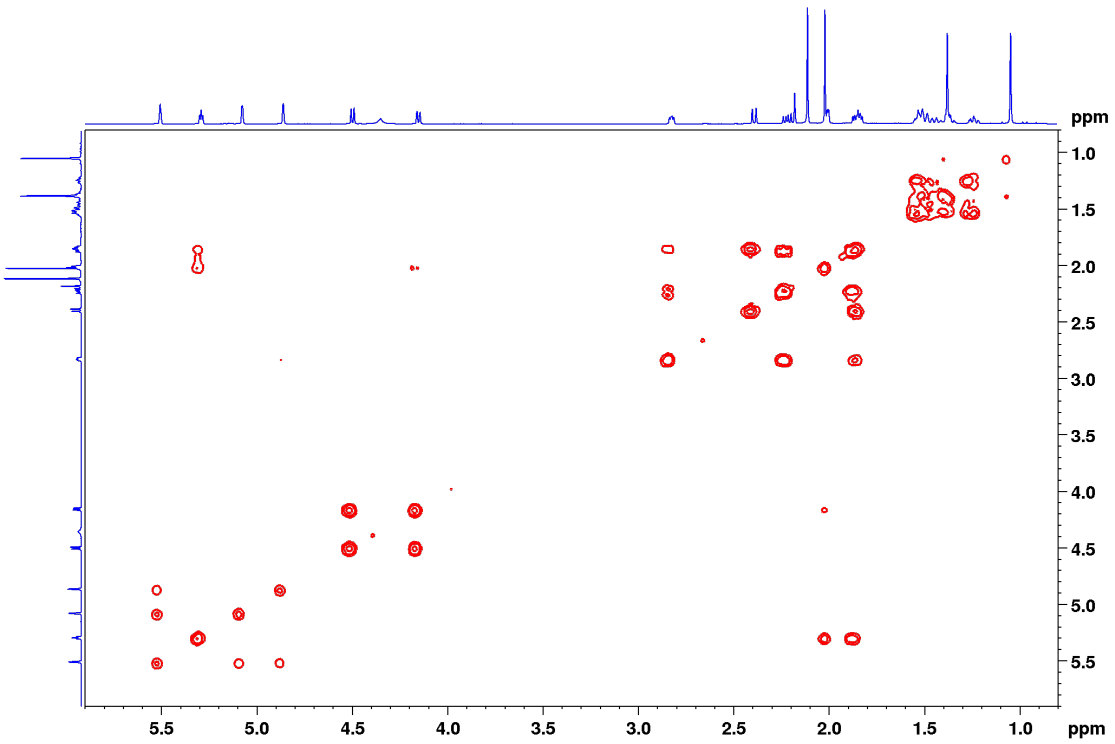

Fig. S25. ^{1}H–^{1}H COSY NMR spectrum for compound **3**

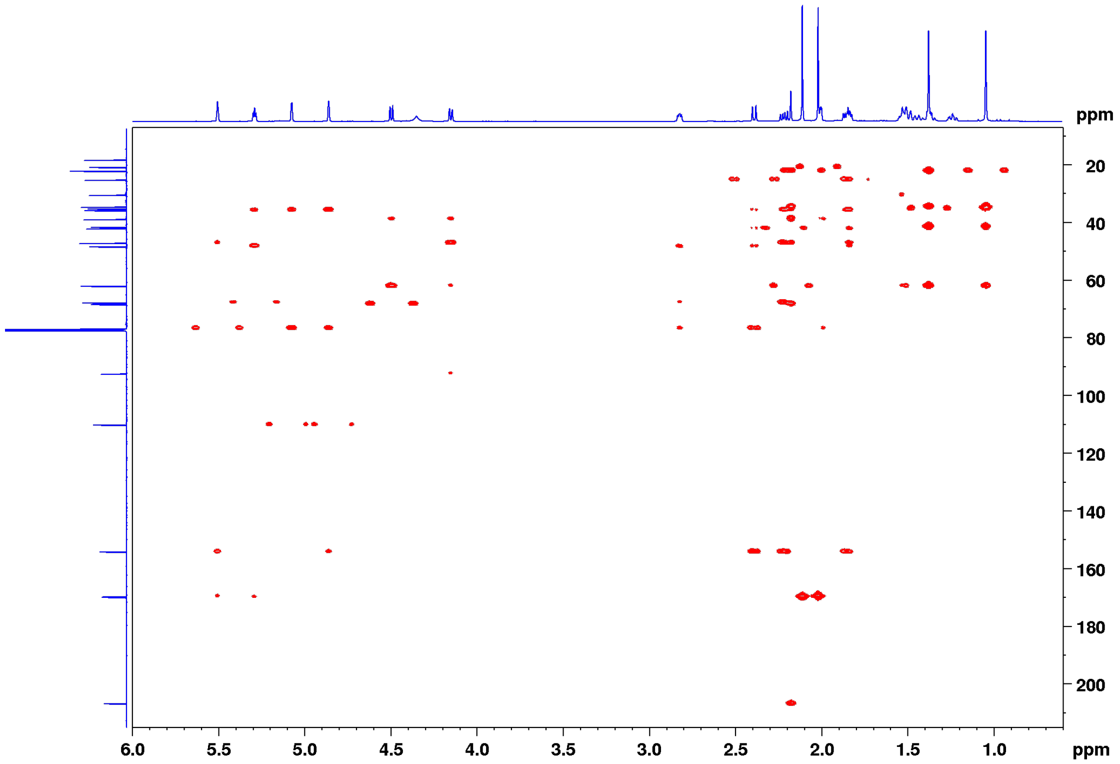

Fig. S26. HMBC NMR spectrum for compound **3**

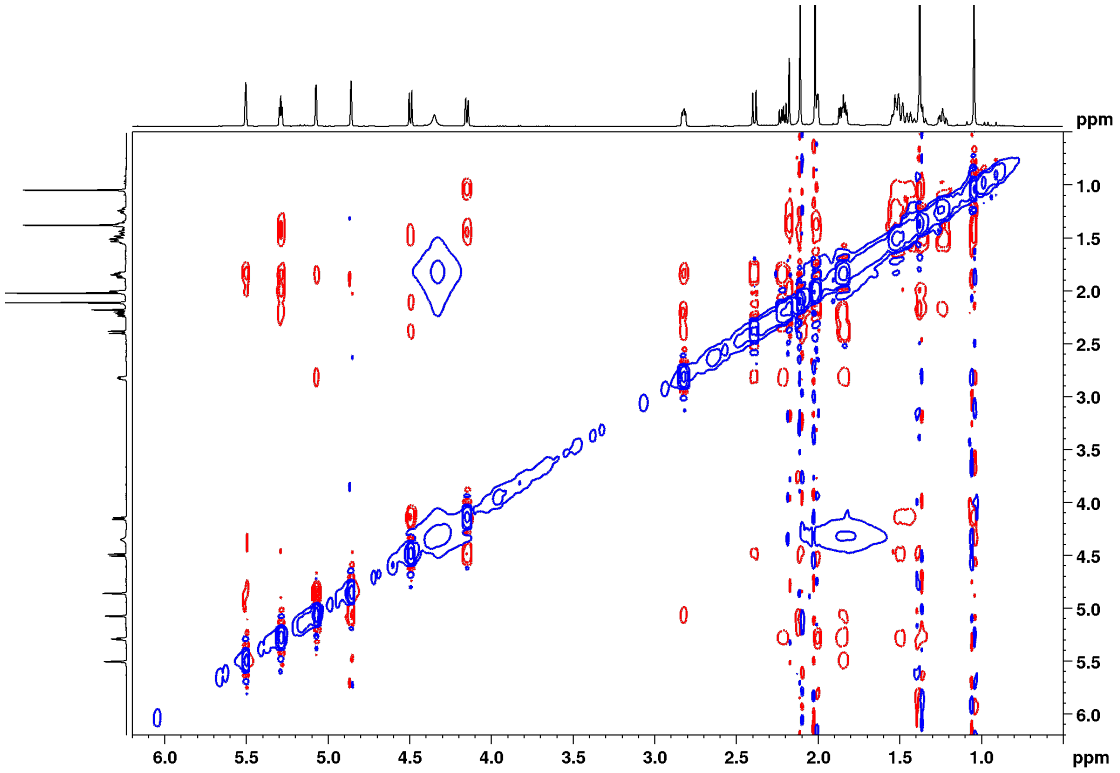

Fig. S27. NOESY NMR spectrum for compound **3**

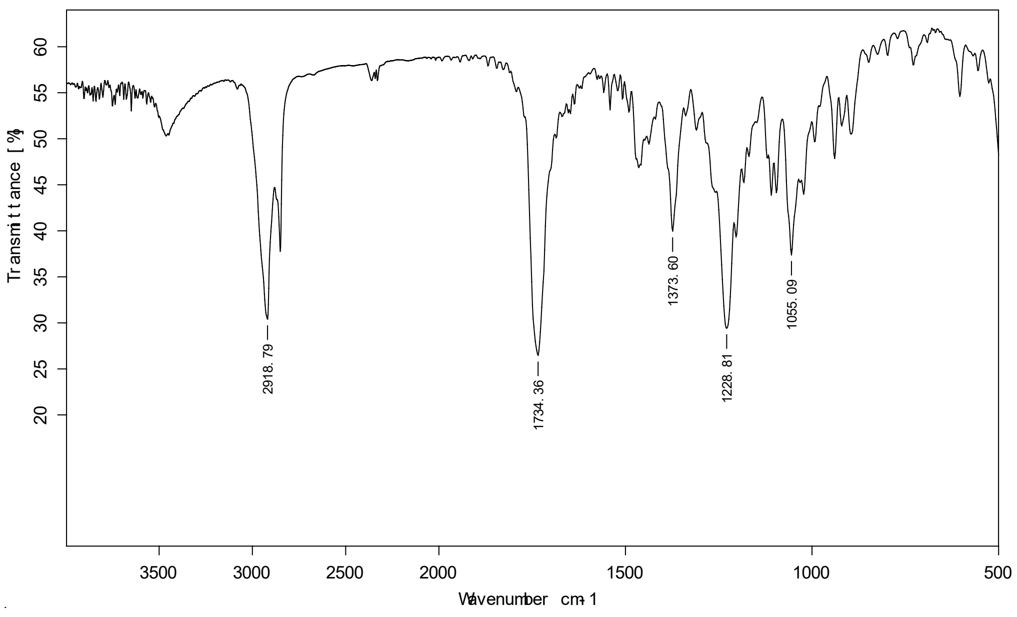

Fig. S28. IR spectrum of compound **3** (KBr)

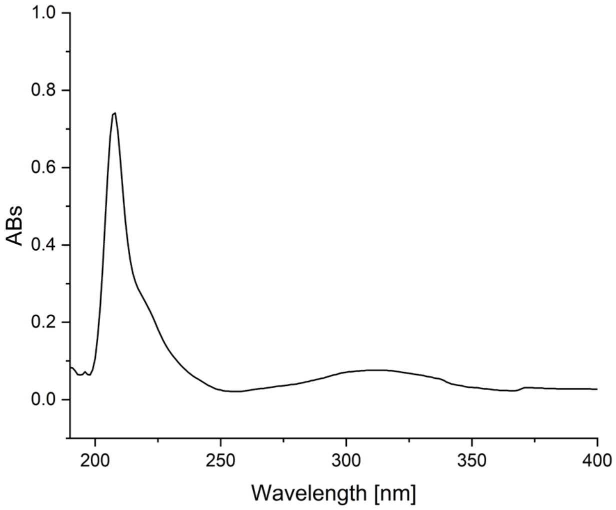

Fig. S29. UV spectrum of compound **3** in MeOH

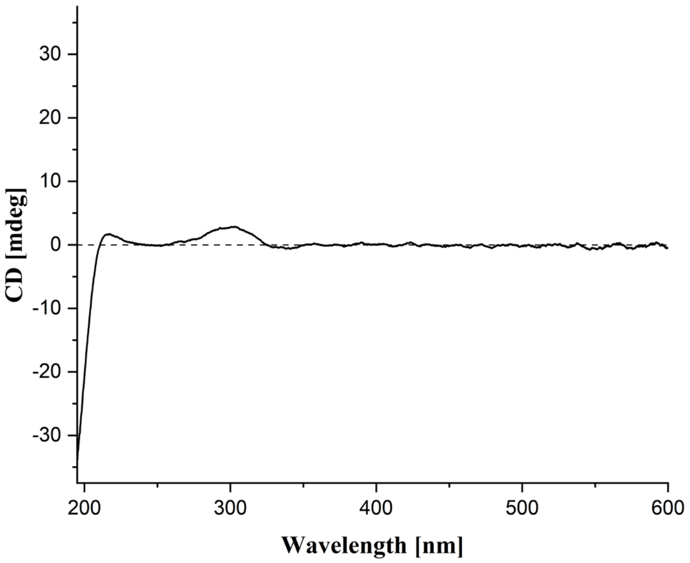

Fig. S30. CD spectrum of compound **3** in MeOH

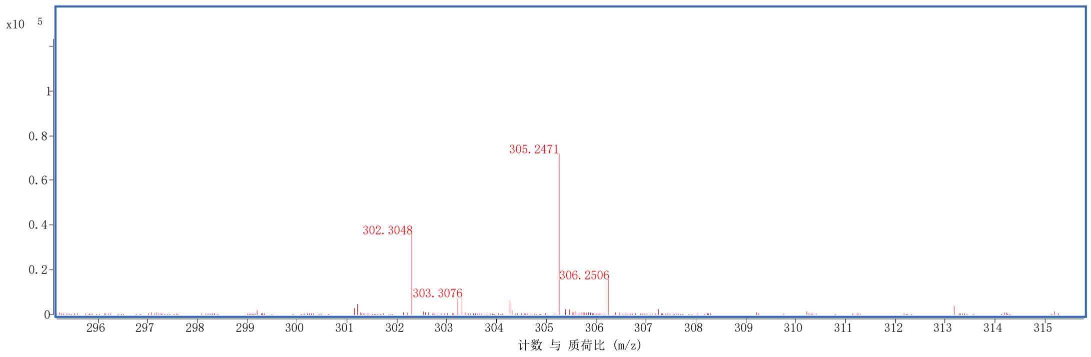

Formula (M)	Score (MFG)	Mass	Mass (MFG)	*m/z* (Calc)	Diff (ppm)	*m/z*
$C_{20}H_{32}O_2$	99.43	304.2398	304.2402	305.2475	1.34	305.2471

Fig. S31. HR-ESI-MS spectrum of compound **4**

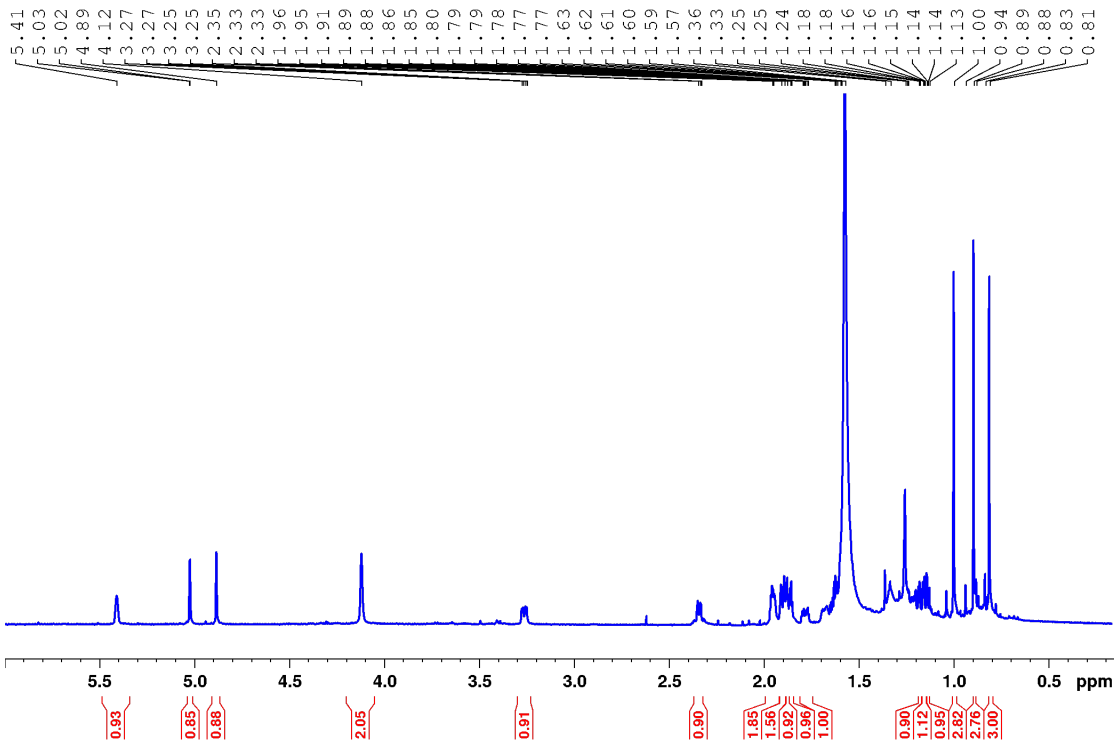

Fig. S32. ^{1}H NMR spectrum for compound **4**

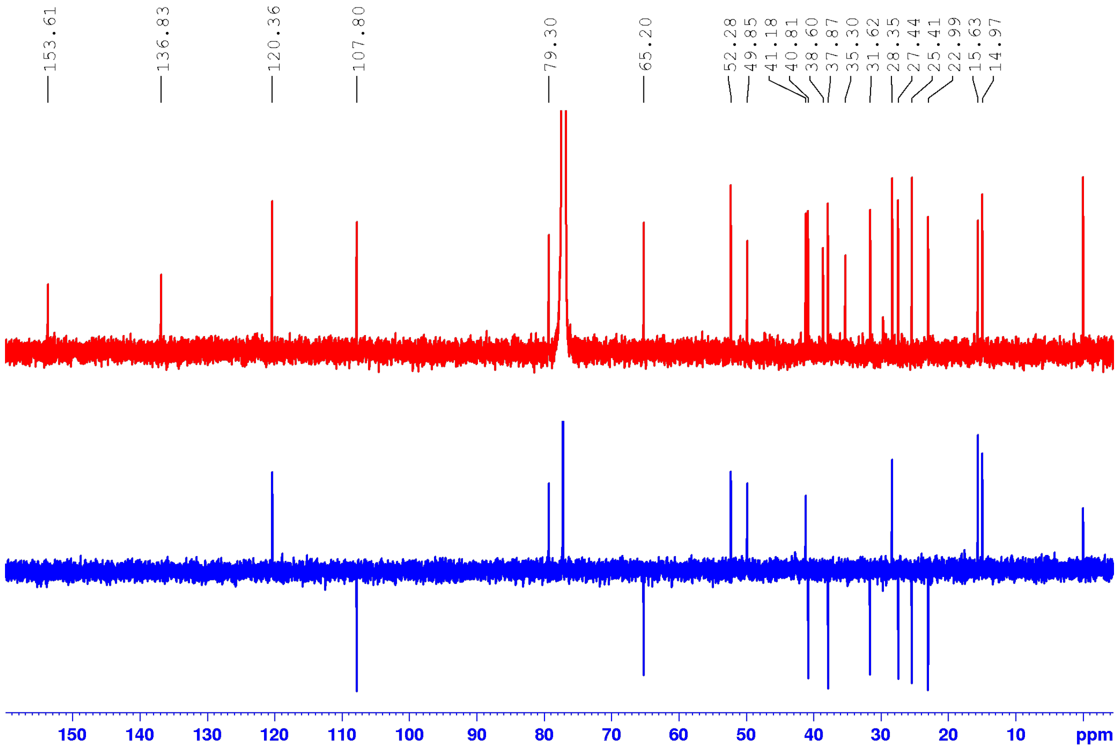

Fig. S33. ^{13}C and DEPT135 NMR spectra for compound **4**

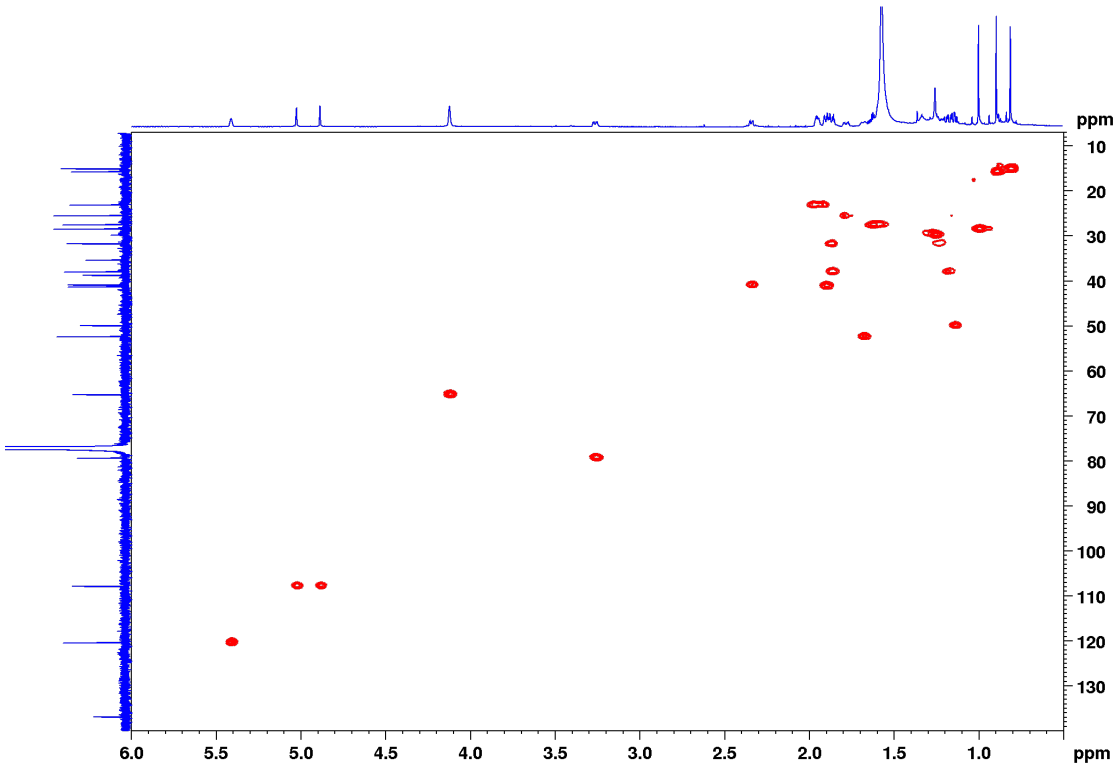

Fig. S34. HSQC NMR spectrum for compound **4**

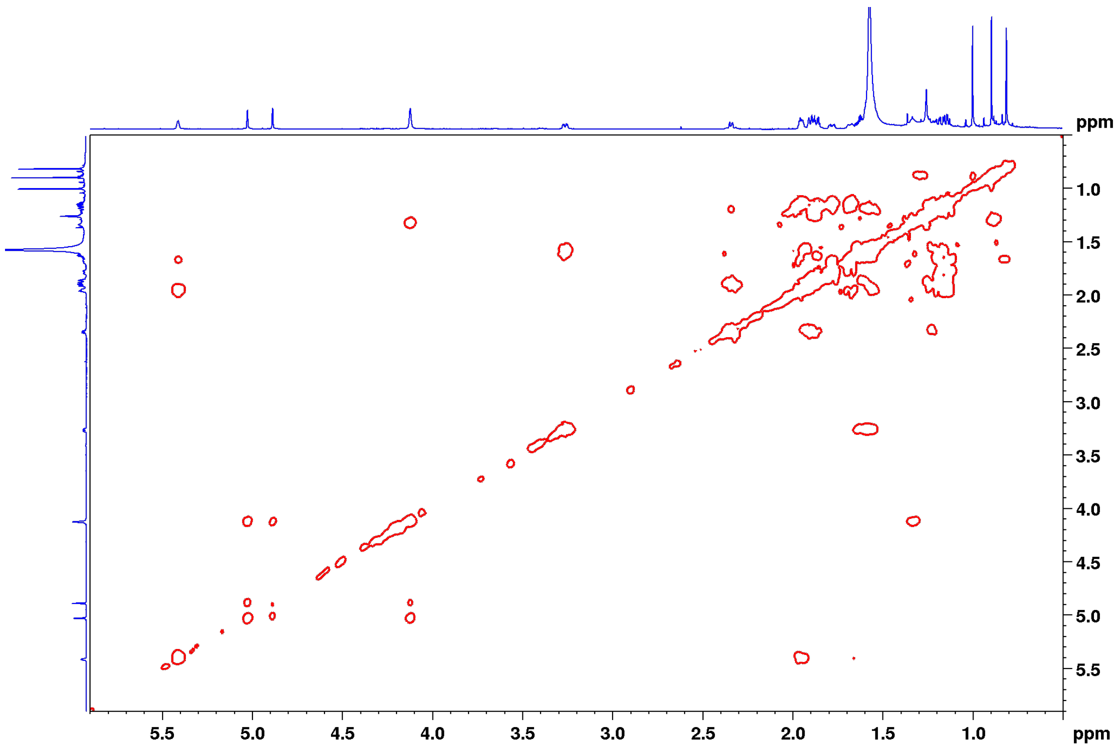

Fig. S35. ^1H–^1H COSY NMR spectrum for compound **4**

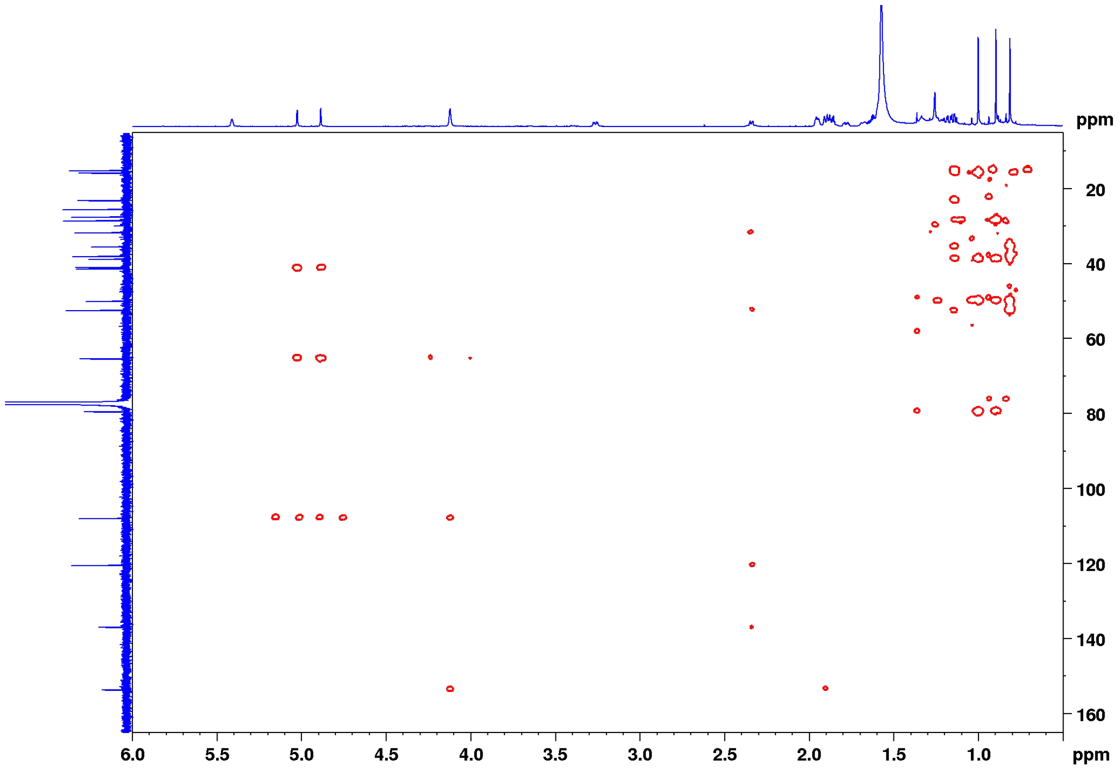

Fig. S36. HMBC NMR spectrum for compound **4**

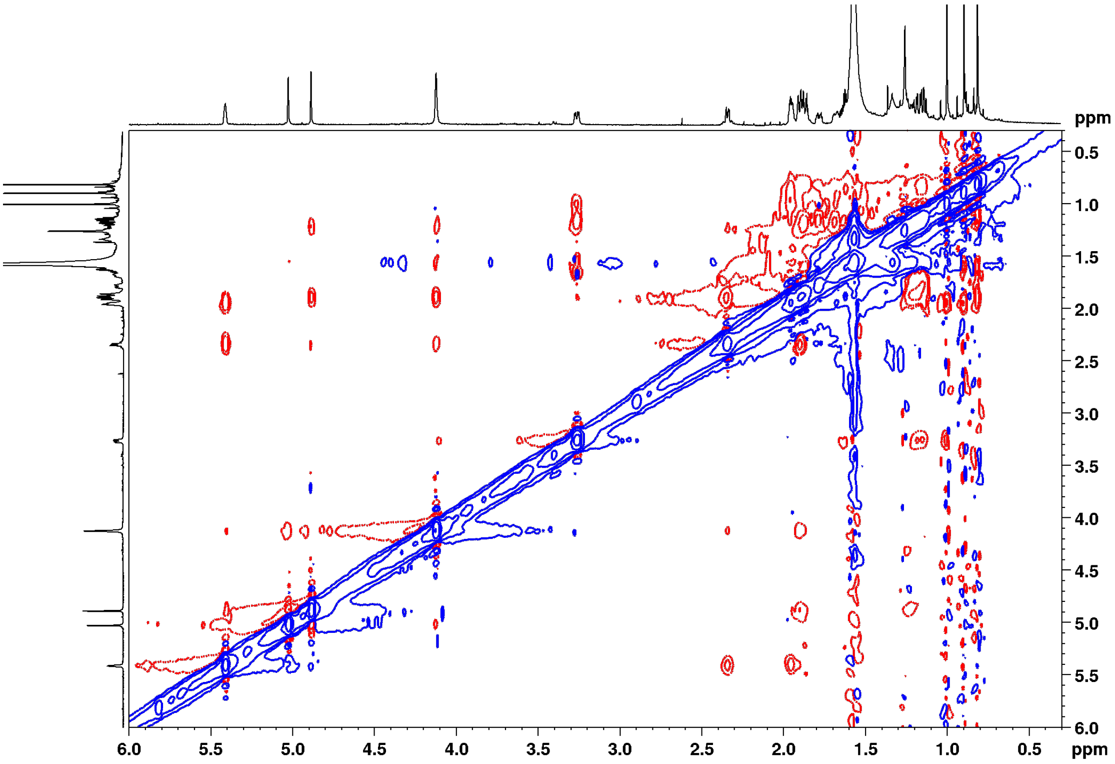

Fig. S37. NOESY NMR spectrum for compound **4**

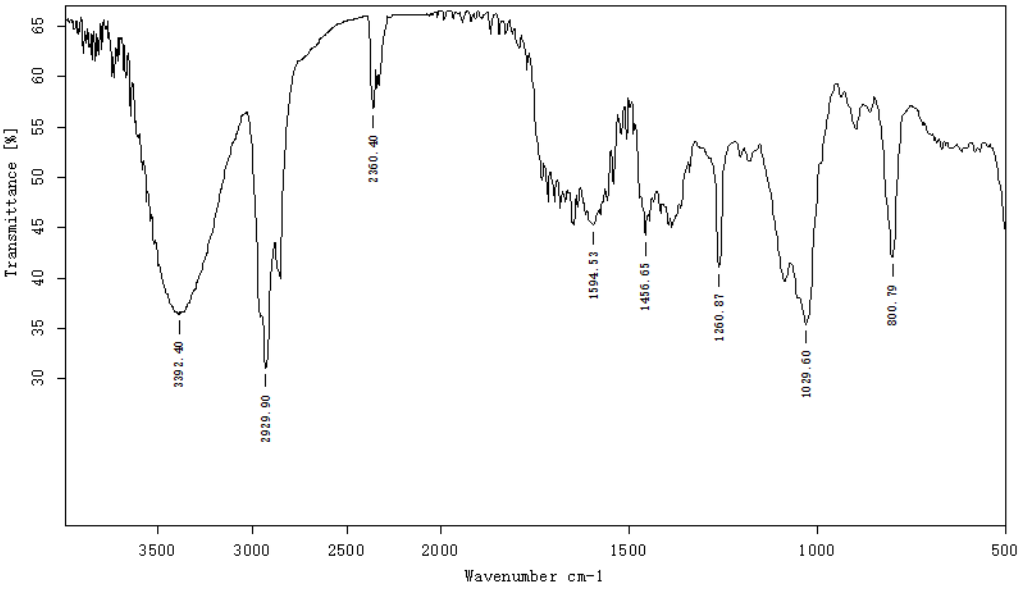

Fig. S38. IR spectrum of compound **4** (KBr)

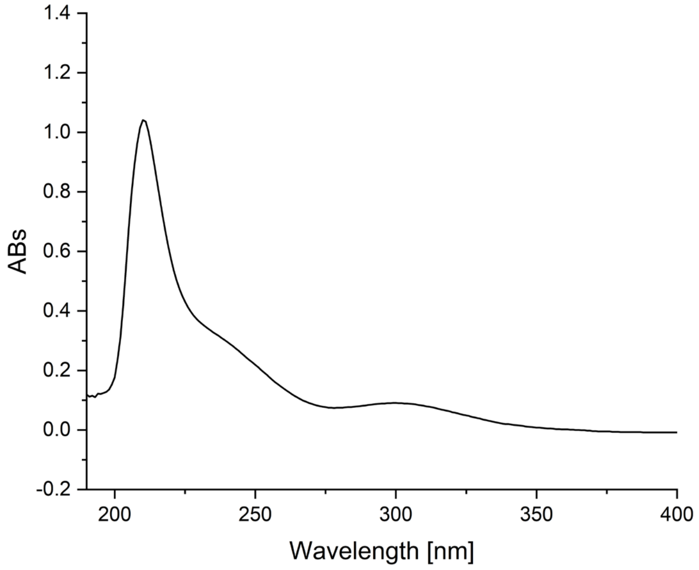

Fig. S39. UV spectrum of compound **4** in MeOH

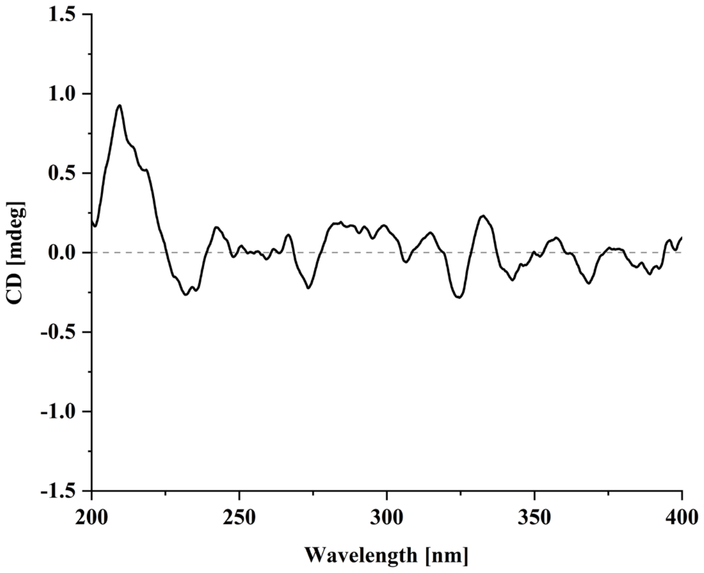

Fig. S40. CD spectrum of compound **4** in MeOH

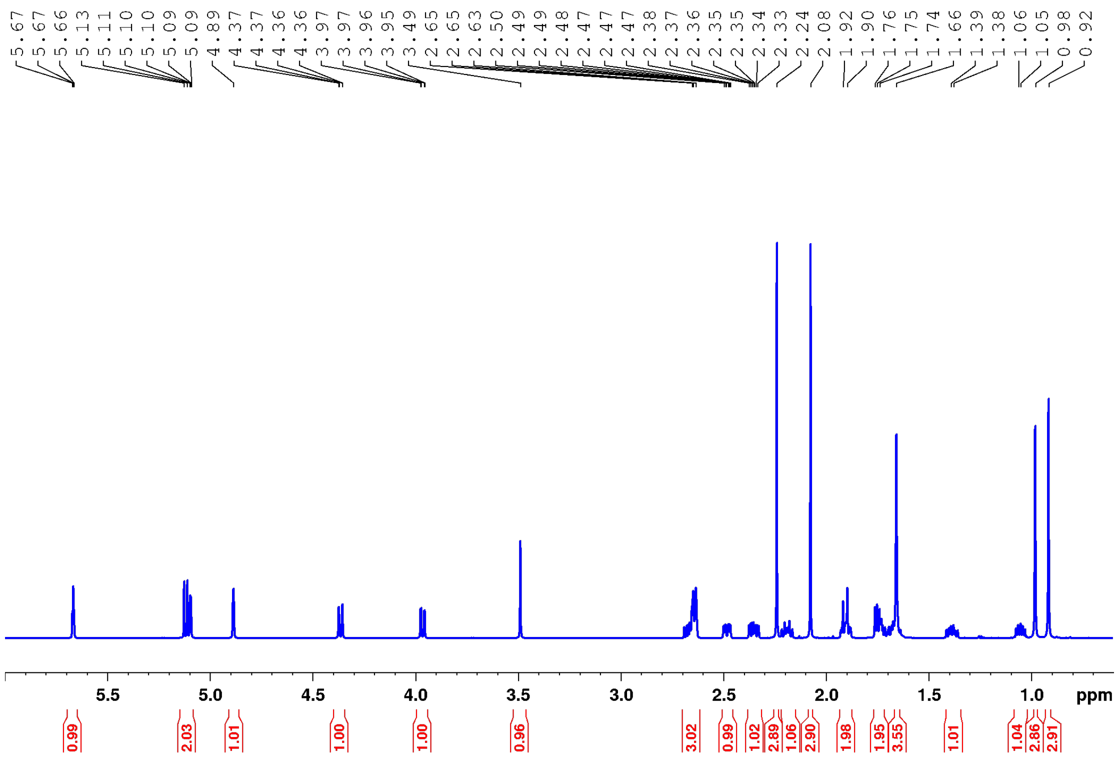

Fig. S41. ^{1}H NMR spectrum for compound **5**

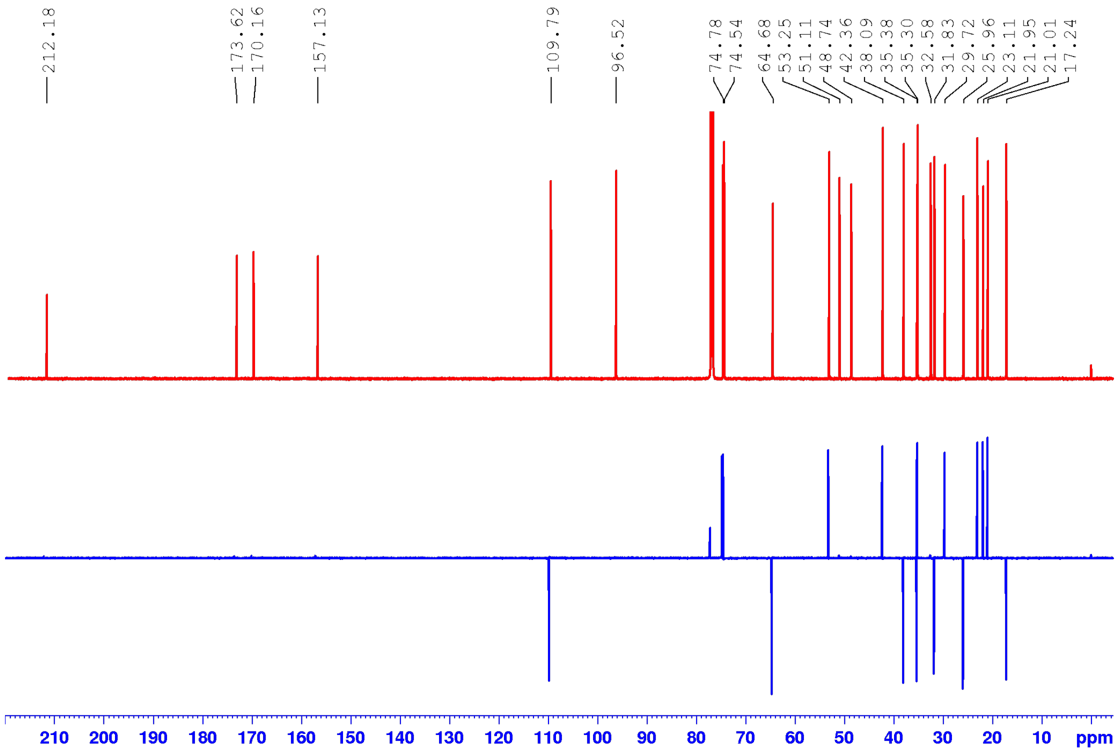

Fig. S42. ^{13}C and DEPT135 NMR spectra for compound **5**

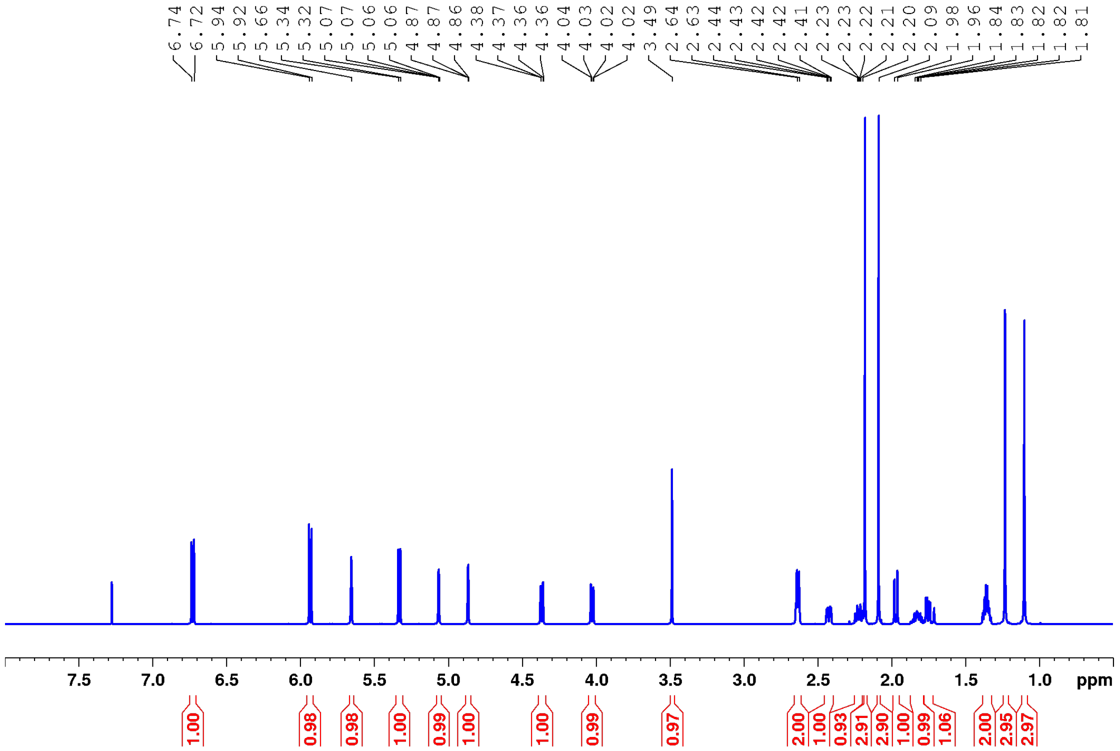

Fig. S43. ^{1}H NMR spectrum for compound **6**

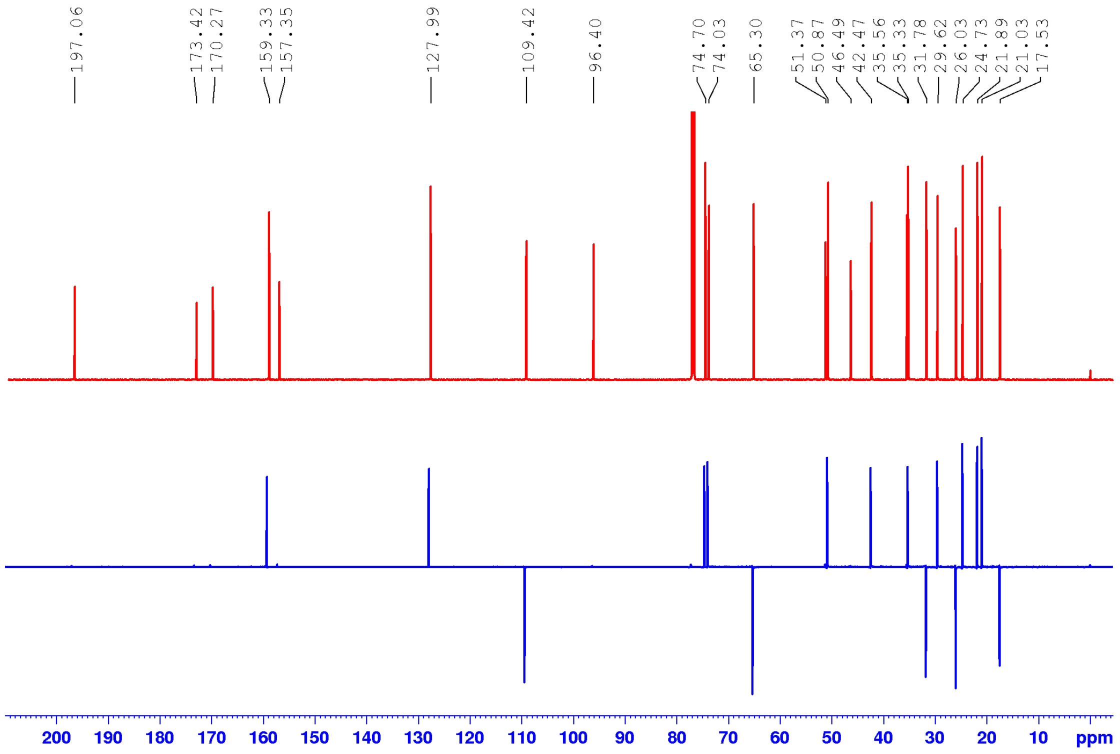

Fig. S44. ^{13}C and DEPT135 NMR spectra for compound **6**

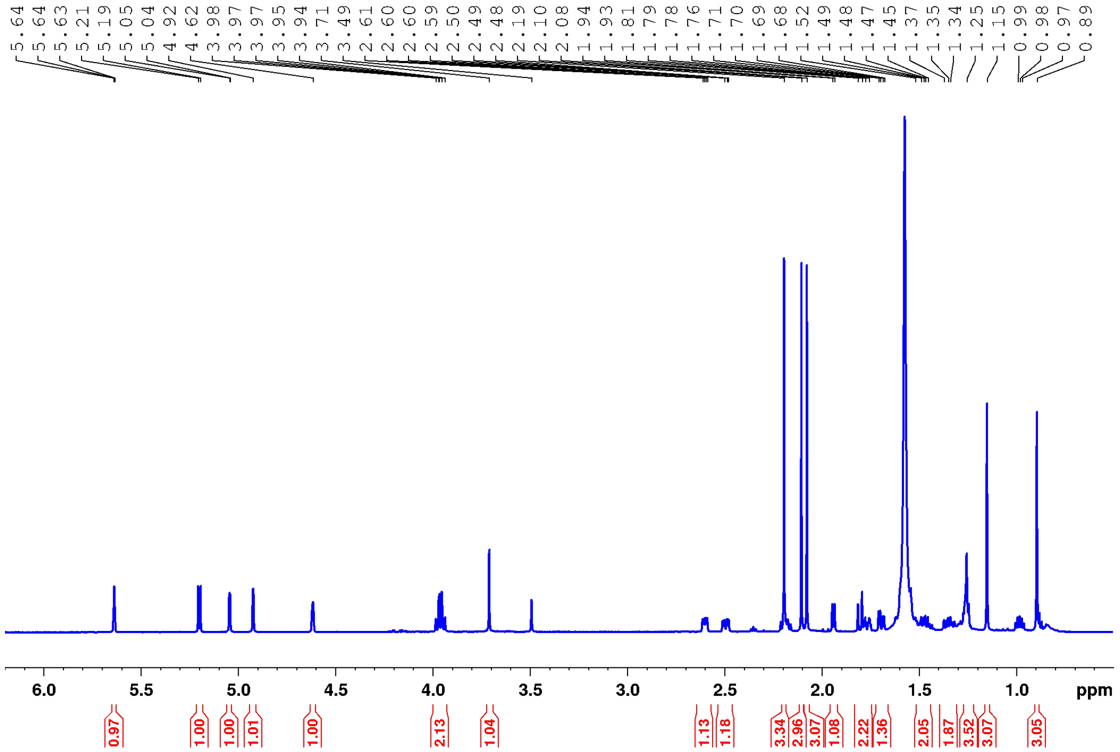

Fig. S45. ^{1}H NMR spectrum for compound **7**

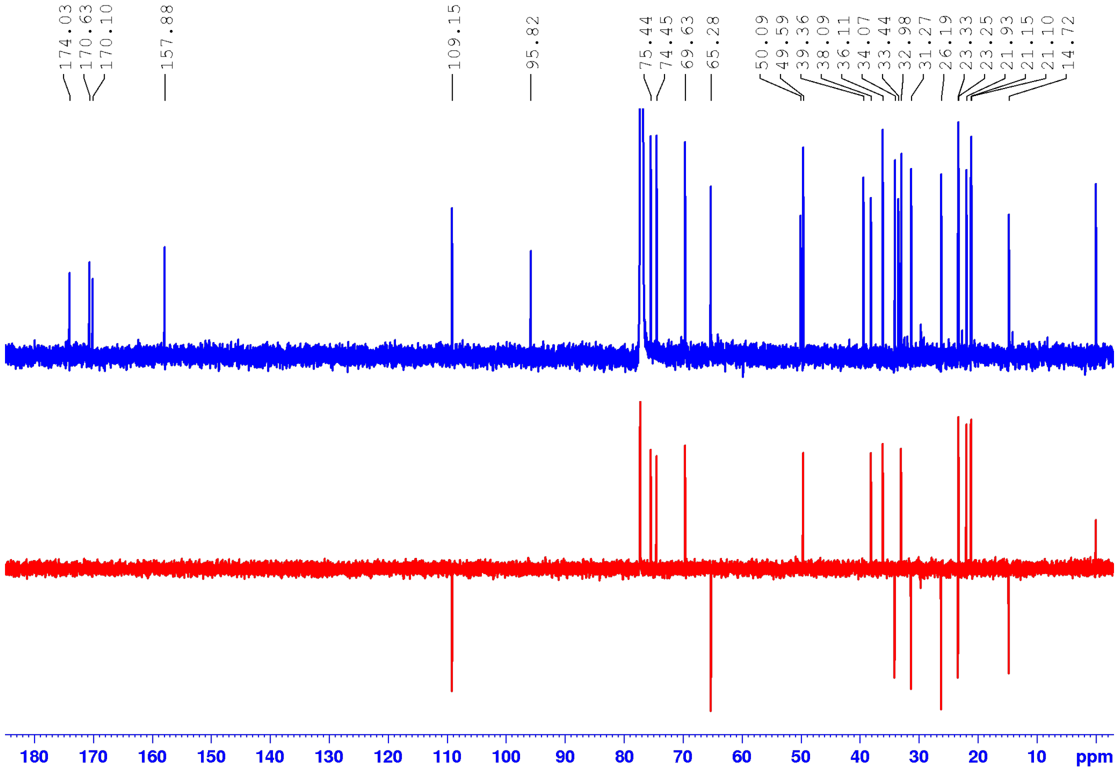

Fig. S46. ^{13}C and DEPT135 NMR spectra for compound **7**

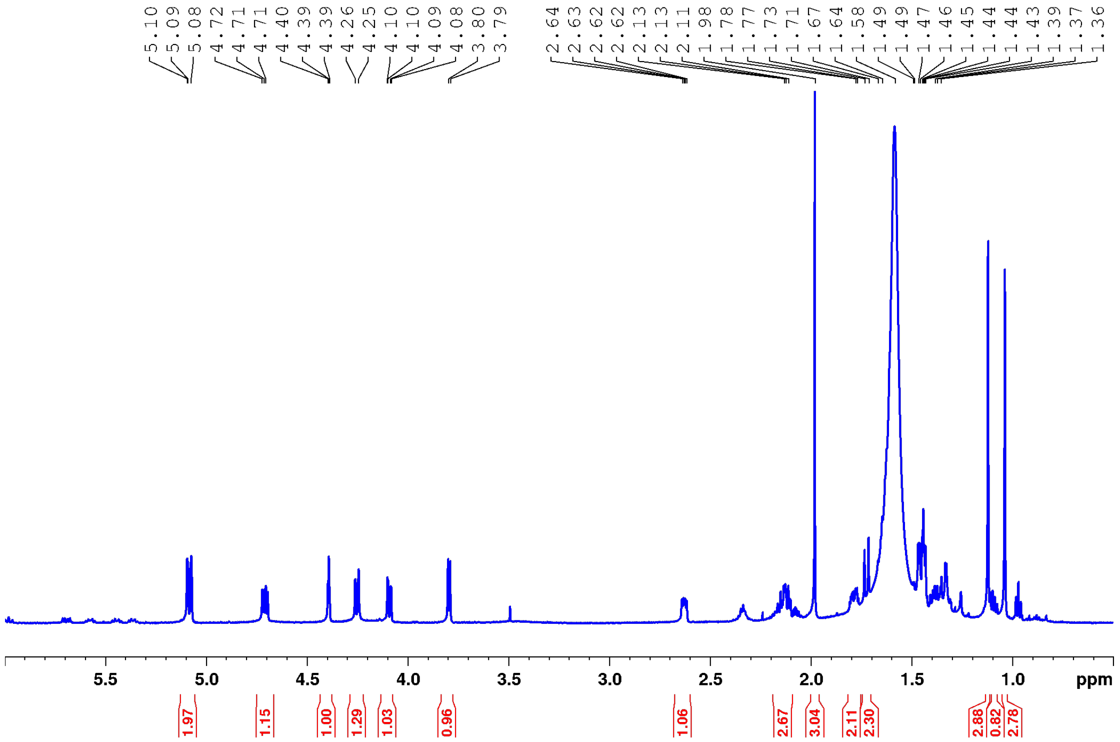

Fig. S47. ^{1}H NMR spectrum for compound **8**

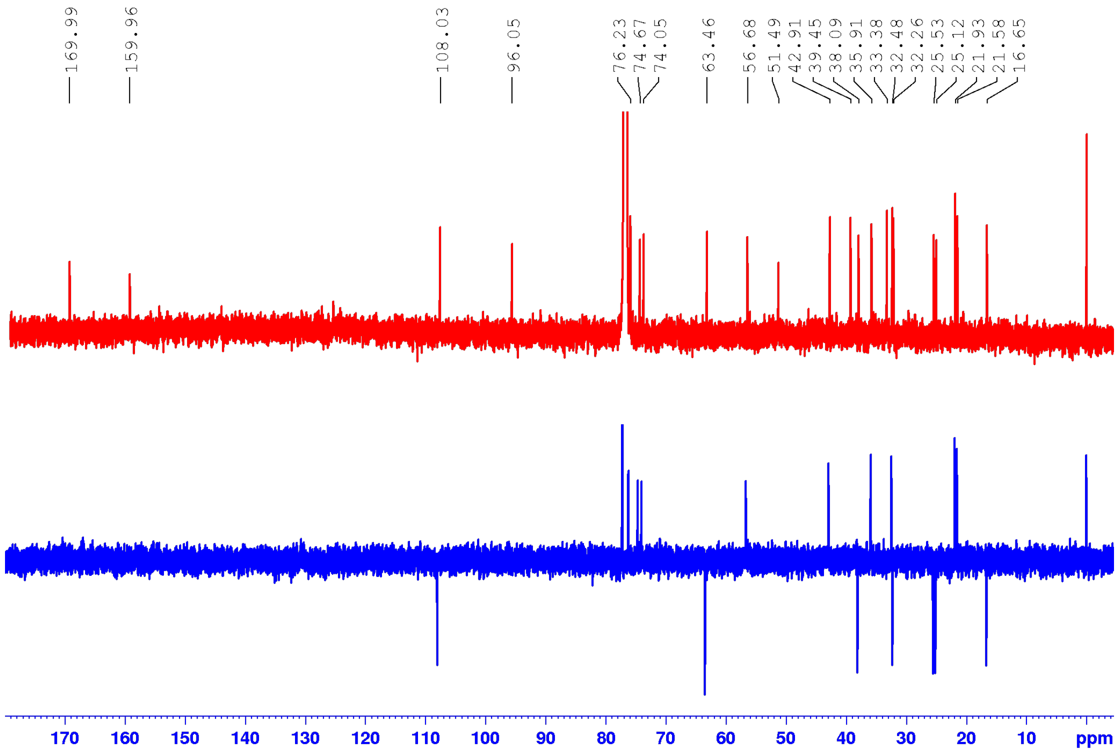

Fig. S48. ^{13}C and DEPT135 NMR spectra for compound **8**

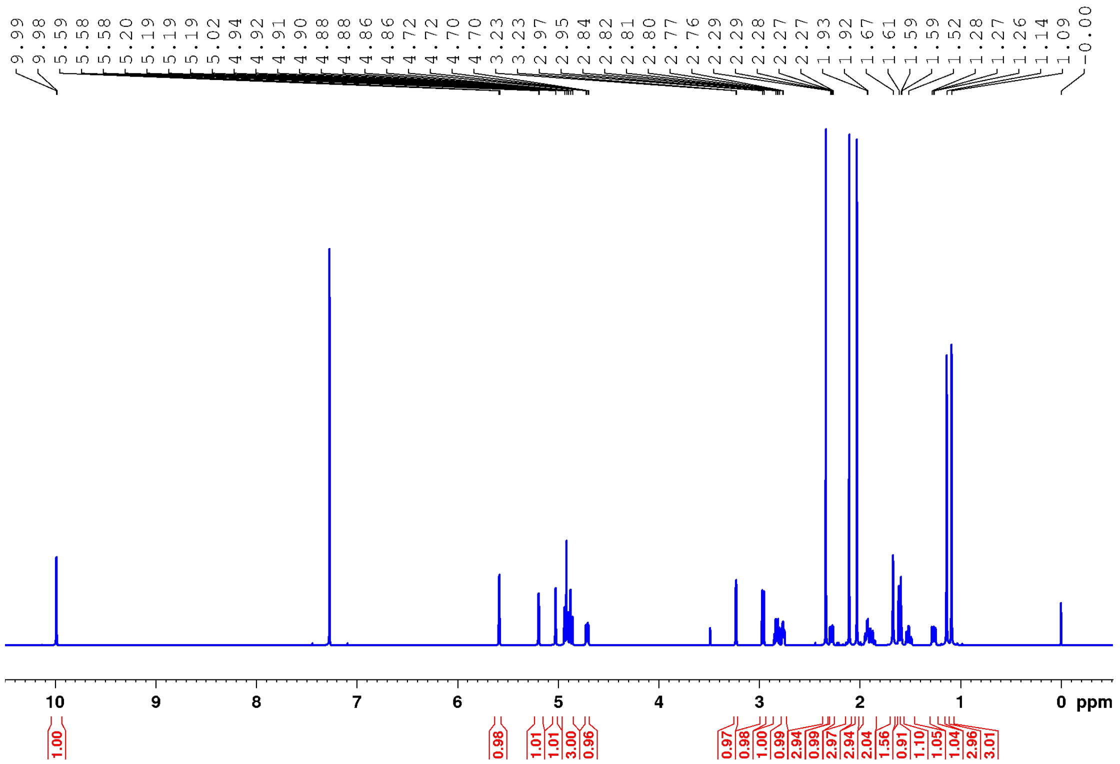

Fig. S49. ^{1}H NMR spectrum for compound **9**

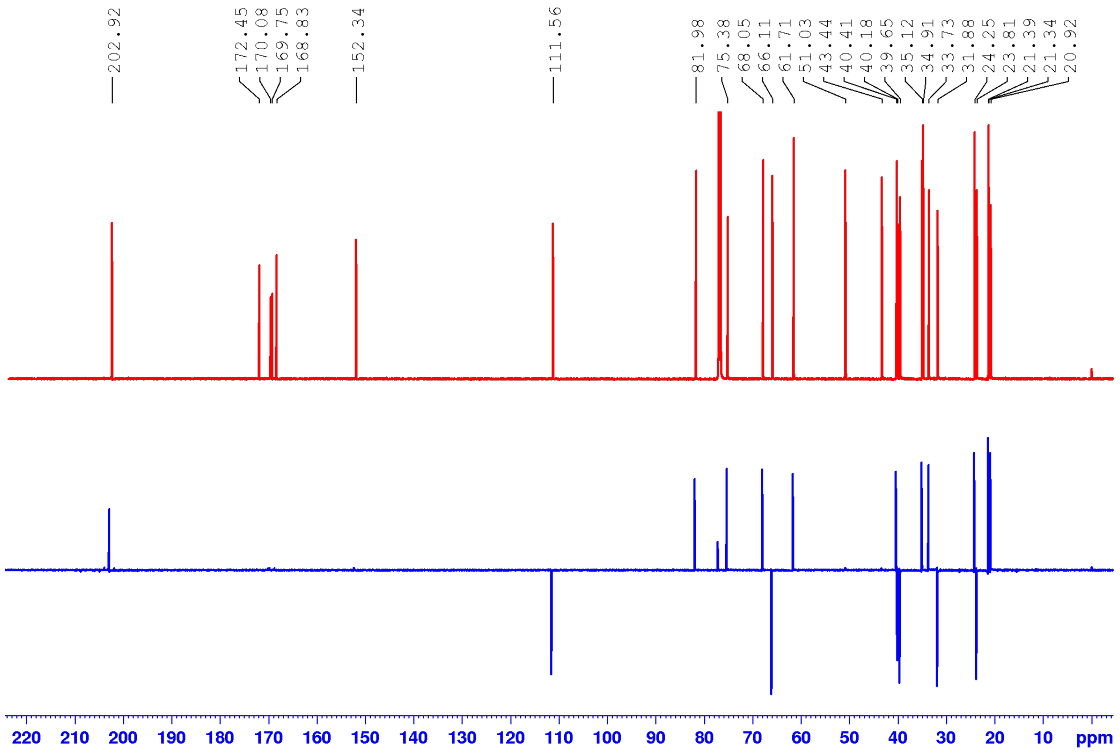

Fig. S50. ^{13}C and DEPT135 NMR spectra for compound **9**

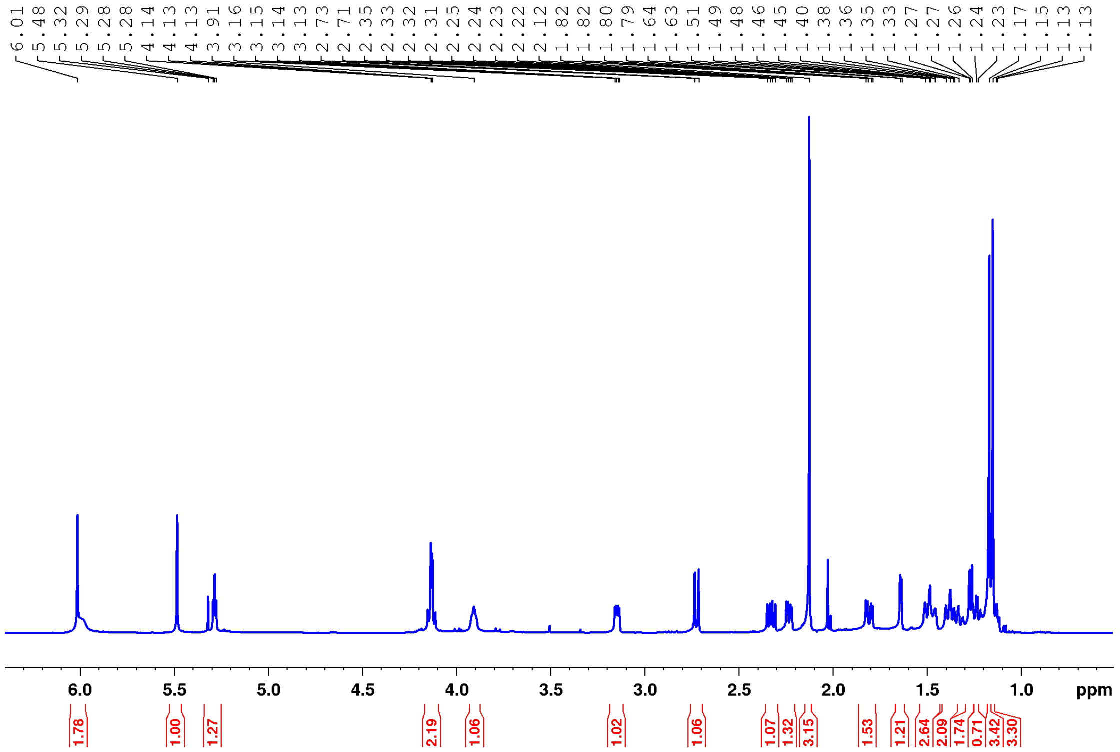

Fig. S51. ^{1}H NMR spectrum for compound **10**

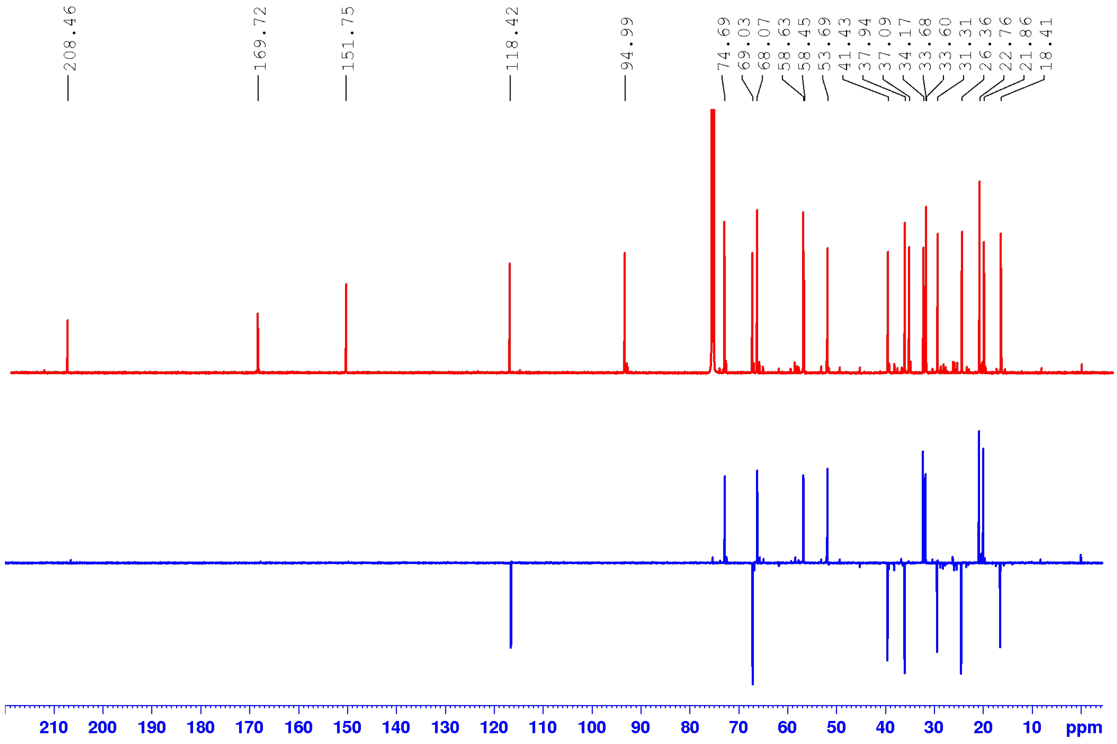

Fig. S52. ^{13}C and DEPT135 NMR spectra for compound **10**

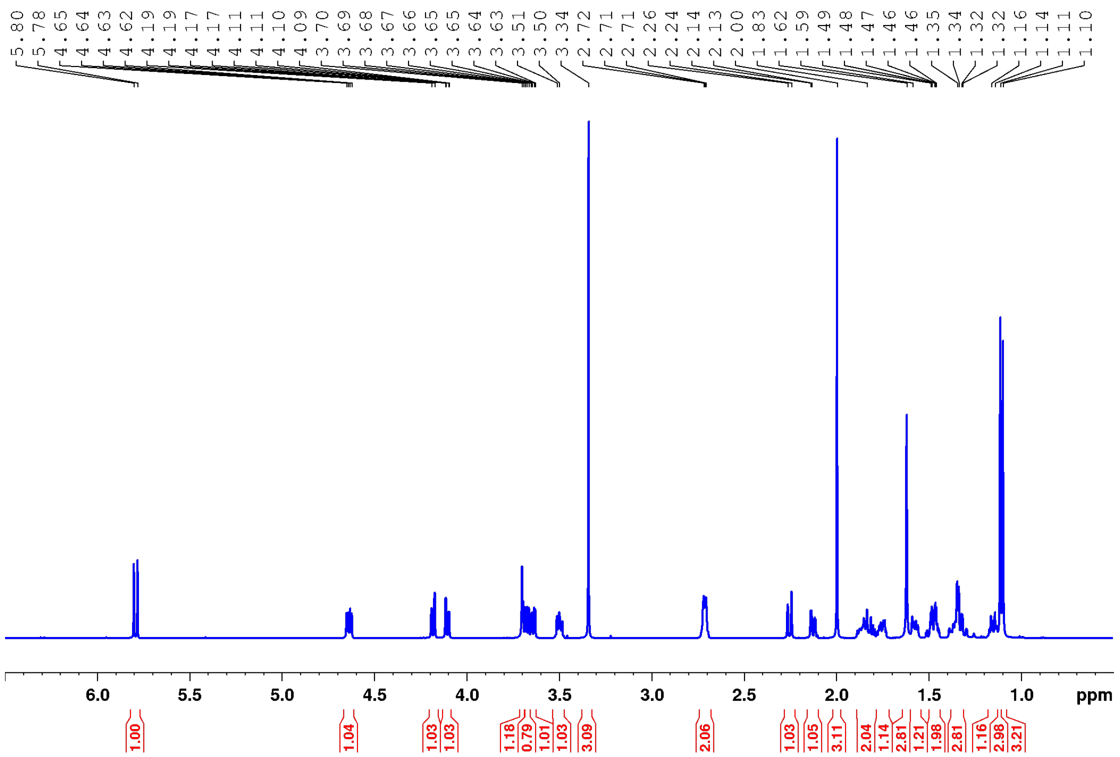

Fig. S53. ^{1}H NMR spectrum for compound **11**

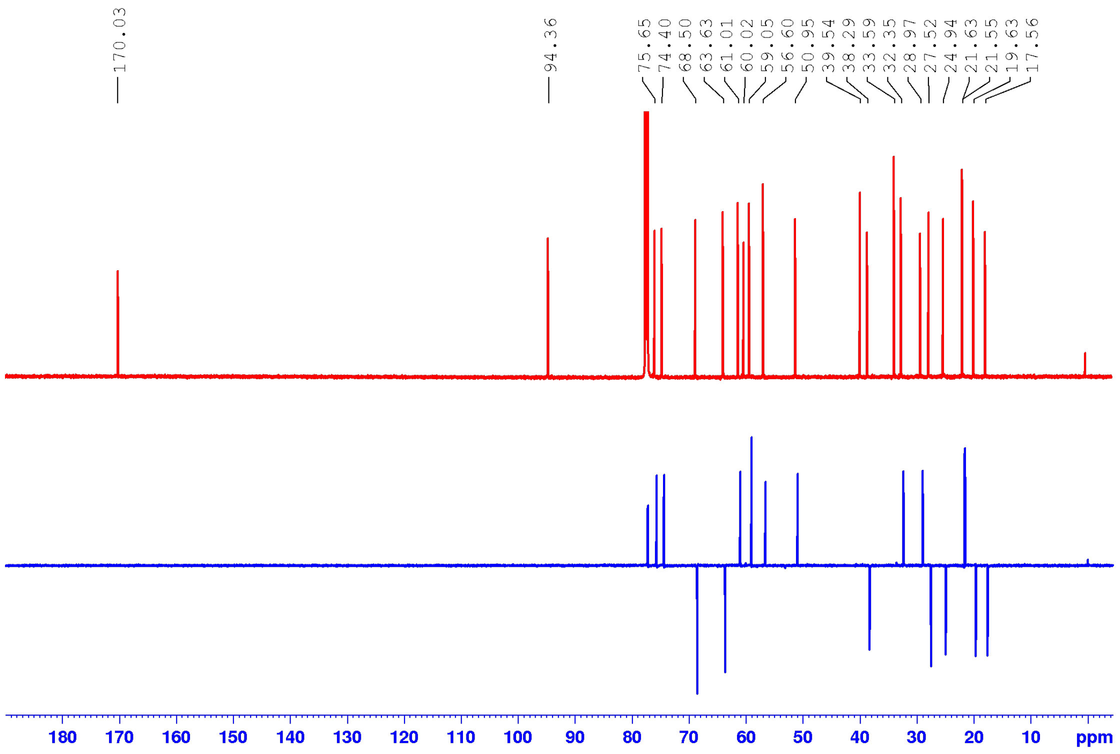

Fig. S54. ^{13}C and DEPT135 NMR spectra for compound **11**

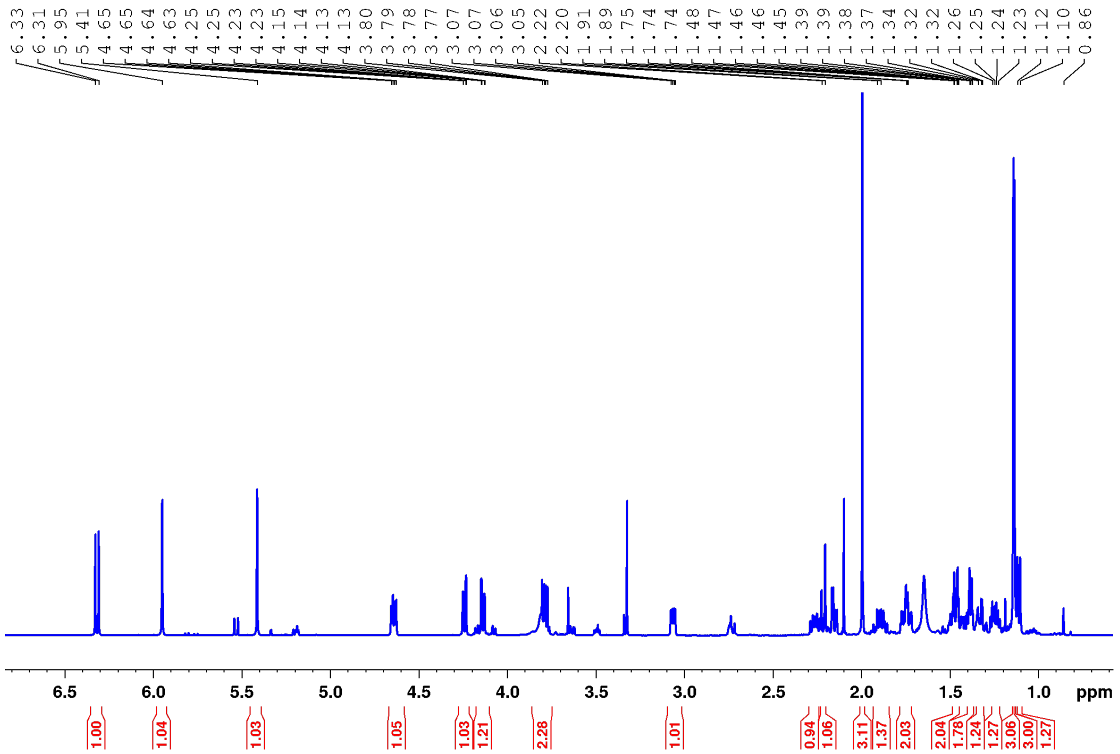

Fig. S55. ^{1}H NMR spectrum for compound **12**

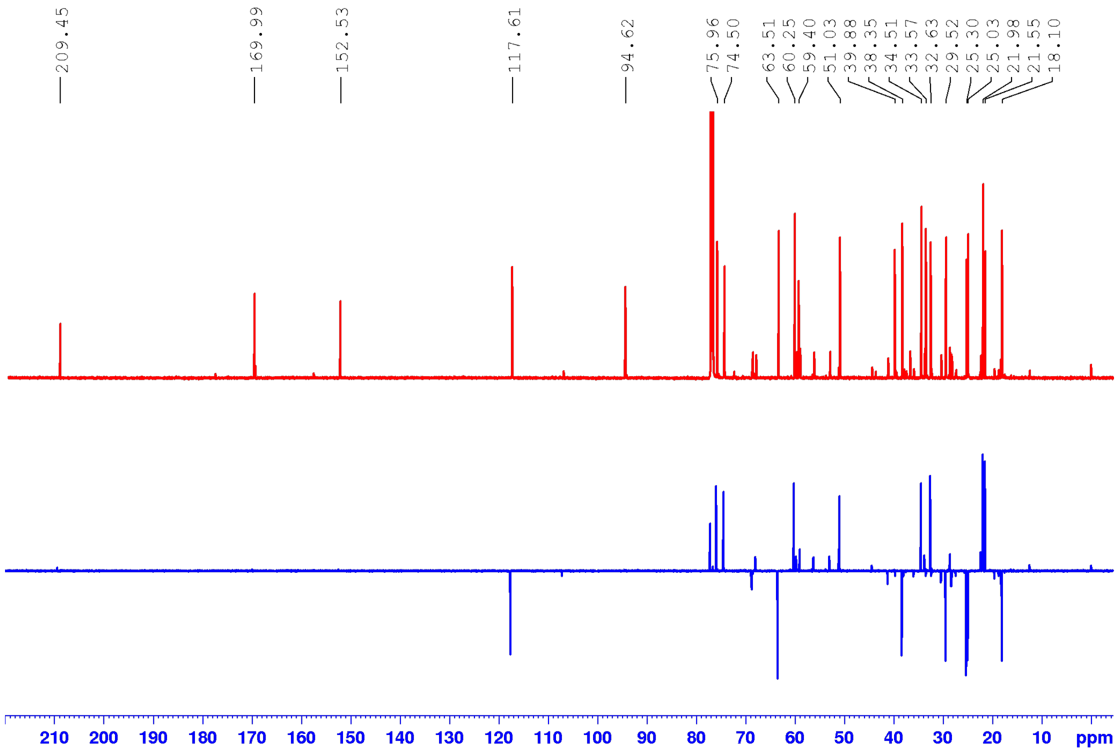

Fig. S56. ^{13}C and DEPT135 NMR spectra for compound **12**

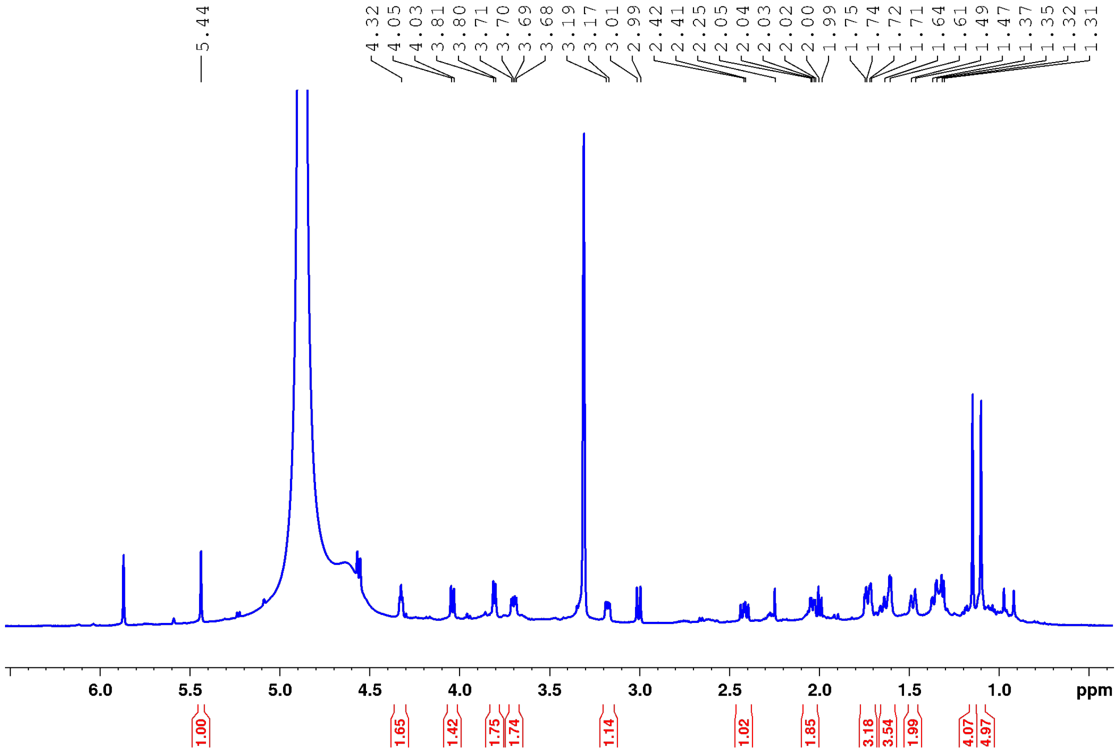

Fig. S57. ^{1}H NMR spectrum for compound **13**

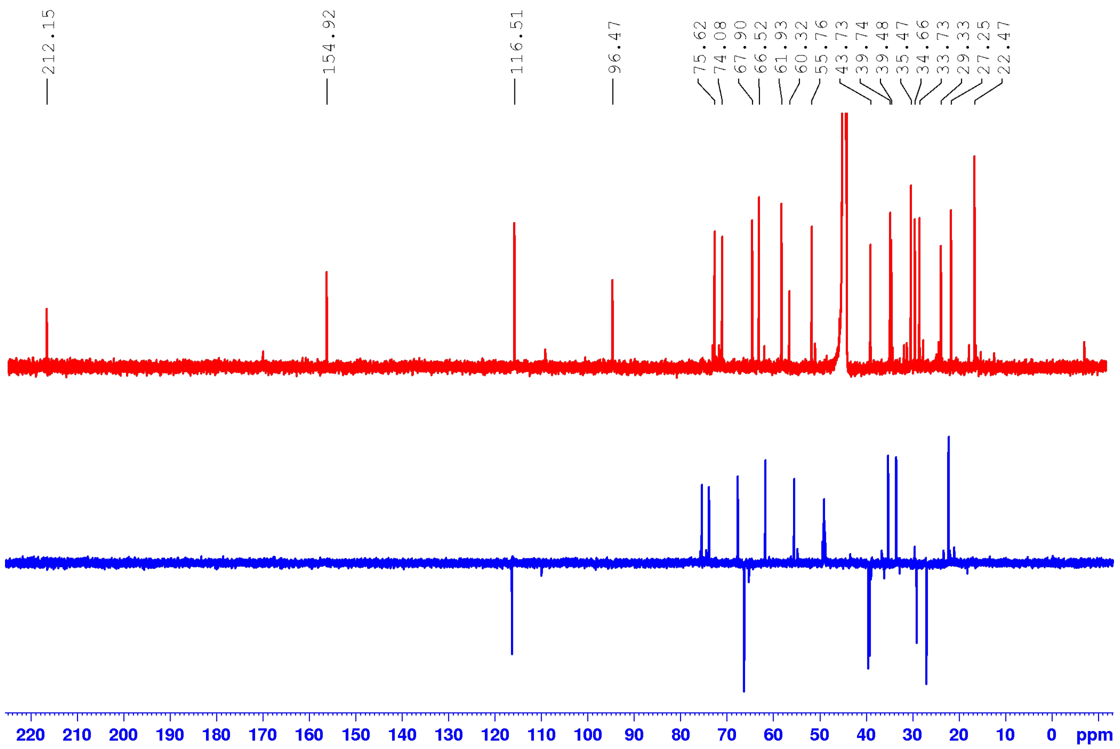

Fig. S58. ^{13}C and DEPT135 NMR spectra for compound **13**

Table S1. Key conformers of compound **1**.

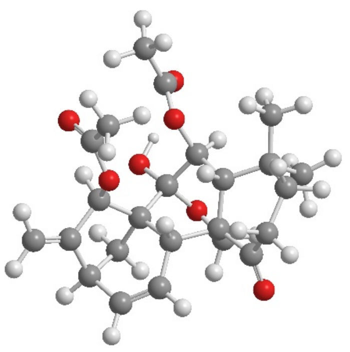	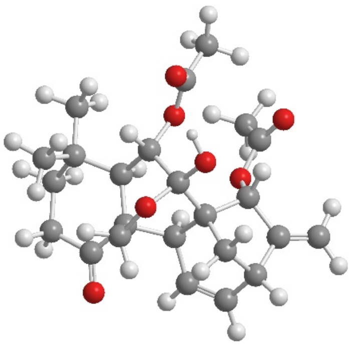	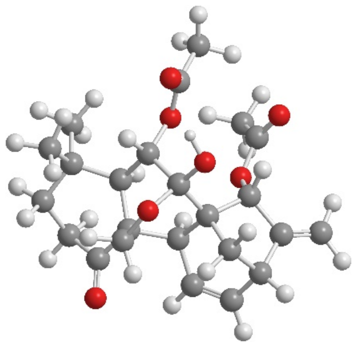
Conformer **1-1** 48.87%	Conformer **1-2** 42.18%	Conformer **1-3** 8.95%

Table S2. Conformers and Bolzmann distributions of the optimized **1**.

species	*E'=E+ZPE*	*E*	*H*	*G*	*ΔG*	*ΔE(kcal/mol)*	*p%*
**1-1**	-1458.68987	-1458.661528	-1458.660584	-1458.746148	0	0	48.87%
**1-2**	-1458.689605	-1458.661347	-1458.660403	-1458.746009	0.000139	0.08722382	42.18%
**1-3**	-1458.688247	-1458.660046	-1458.659102	-1458.744546	0.001602	1.005270219	8.95%

E, *E'*, *H, G*: total energy, total energy with zero point energy (ZPE), enthalpy, and Gibbs free energy

Table S3. Optimized Z-matrixes of isomer **1** in the gas phase (Å) at B3LYP/6-31G(d,p) level.

**1-1**					**1-2**			
atom	X	Y	Z		atom	X	Y	Z
C	2.911268	-1.37772	2.033586		C	-3.73295	-1.40097	-1.40847
C	3.51014	0.040026	1.811255		C	-3.64387	0.103526	-1.67438
C	3.022372	0.764307	0.517436		C	-3.03717	0.899001	-0.49672
C	1.562057	0.288653	0.249312		C	-1.59929	0.328011	-0.28848
C	1.422672	-1.24127	-0.06152		C	-1.46936	-1.2165	-0.02574
C	2.437396	-2.03857	0.753092		C	-2.51215	-2.06202	-0.77162
C	0.756198	1.066146	-0.80444		C	-0.74421	1.07378	0.756724
C	-0.17892	0.097646	-1.56789		C	0.134576	0.078595	1.541357
C	-1.01076	-0.89348	-0.71478		C	0.960987	-0.91968	0.698896
C	-0.05742	-1.60855	0.320258		C	0.025785	-1.5677	-0.39102
C	-1.70176	-1.91494	-1.65622		C	1.574824	-1.99223	1.639479
C	-2.56326	-2.69942	-0.65454		C	2.496561	-2.74181	0.668814
C	-1.60025	-3.54885	0.161168		C	1.59519	-3.50292	-0.28946
C	-0.40458	-3.06679	0.515316		C	0.424433	-2.99673	-0.6914
O	2.826585	-3.13689	0.394709		O	-2.43736	-3.27907	-0.76602
C	3.05227	2.284895	0.769808		C	-2.94466	2.381822	-0.91332
C	1.576598	-1.5567	-1.56378		C	-1.64757	-1.54533	1.480846
O	0.754474	-0.68046	-2.34705		O	-0.85172	-0.67502	2.285327
C	3.993183	0.451805	-0.64195		C	-3.95151	0.812784	0.742978
O	-1.03015	0.751512	-2.43966		O	0.969938	0.700482	2.450844

C	-2.27018	-0.34007	0.006157		C	2.267725	-0.3696	0.054875
C	-3.17463	-1.5654	0.167833		C	3.19845	-1.58203	-0.02835
C	-4.29869	-1.59705	0.879397		C	4.410518	-1.57628	-0.57826
O	0.050867	2.15403	-0.14445		O	0.020948	2.115833	0.087636
O	0.019359	3.33007	-2.08351		O	0.069677	3.324587	2.006447
C	-0.22198	3.252073	-0.89268		C	0.323048	3.21703	0.820073
C	-0.85952	4.340354	-0.07364		C	1.008399	4.269587	-0.00688
O	-1.96544	0.255425	1.289325		O	2.039043	0.201147	-1.25711
O	-3.20682	2.104537	0.842329		O	3.34863	2.002072	-0.81462
C	-2.47621	1.473082	1.576705		C	2.616944	1.381912	-1.55895
C	-1.99262	1.941355	2.927951		C	2.208229	1.829861	-2.94175
H	2.03019	-1.29548	2.685413		H	-3.95837	-1.96305	-2.32051
H	3.619222	-2.04704	2.526789		H	-4.56518	-1.61884	-0.72548
H	3.265109	0.654991	2.683967		H	-3.02809	0.281708	-2.56593
H	4.602652	-0.02868	1.782617		H	-4.64247	0.490885	-1.91025
H	1.027694	0.458992	1.192029		H	-1.0965	0.501965	-1.24762
H	1.39778	1.509728	-1.56918		H	-1.36582	1.563264	1.509017
H	-0.23534	-1.13867	1.294074		H	0.215548	-1.03047	-1.32649
H	-2.31086	-1.37201	-2.38419		H	2.136778	-1.49256	2.433531
H	-0.9981	-2.54558	-2.19807		H	0.828756	-2.63985	2.099292
H	-3.32954	-3.32315	-1.12213		H	3.203749	-3.41602	1.159342
H	-1.88808	-4.56484	0.419241		H	1.918673	-4.48022	-0.63909
H	0.30487	-3.68862	1.054346		H	-0.21801	-3.56442	-1.35334
H	2.835763	2.853022	-0.1407		H	-2.5832	3.013403	-0.09631
H	4.047053	2.588622	1.113948		H	-3.93485	2.753527	-1.19873
H	2.326061	2.580321	1.533978		H	-2.27415	2.519362	-1.76811
H	1.285576	-2.59939	-1.73463		H	-1.35716	-2.59169	1.636083
H	2.605452	-1.44394	-1.9097		H	-2.68467	-1.43759	1.811885
H	4.200439	-0.61734	-0.7409		H	-4.19153	-0.21252	1.03158
H	3.616282	0.814565	-1.60367		H	-3.50524	1.302546	1.613626
H	4.953037	0.946538	-0.45846		H	-4.90007	1.320083	0.5361
H	-0.59156	1.558156	-2.76144		H	0.561922	1.536987	2.734318
H	-2.74627	0.422253	-0.61425		H	2.695202	0.407332	0.691217
H	-4.65339	-0.72737	1.424825		H	4.825986	-0.68155	-1.03264
H	-4.90567	-2.49553	0.943347		H	5.031458	-2.4674	-0.59216
H	-0.91907	5.249551	-0.67071		H	2.018311	3.924613	-0.25143
H	-1.865	4.019998	0.217965		H	0.472289	4.434405	-0.94499
H	-0.28616	4.522048	0.839224		H	1.063947	5.194198	0.566582
H	-2.59999	2.782283	3.261917		H	2.839114	2.659748	-3.25904
H	-2.0295	1.12915	3.657266		H	2.279076	1.003019	-3.6522
H	-0.95002	2.262426	2.83641		H	1.163602	2.156156	-2.91377
		**1-3**						
atom	X	Y	Z					

C	-3.20428	-1.46344	-1.83247					
C	-4.00641	-0.42111	-1.03389					
C	-3.12779	0.717282	-0.43276					
C	-1.63442	0.227005	-0.27102					
C	-1.39331	-1.30074	-0.00199					
C	-2.32292	-2.18384	-0.83611					
C	-0.7999	1.026067	0.754207					
C	0.139224	0.085555	1.540042					
C	1.021324	-0.87007	0.709706					
C	0.117904	-1.56815	-0.36902					
C	1.691513	-1.90284	1.654936					
C	2.636265	-2.62102	0.681354					
C	1.757517	-3.43941	-0.25078					
C	0.563836	-2.98636	-0.64843					
O	-2.40133	-3.38445	-0.641					
C	-3.12315	1.908192	-1.41734					
C	-1.57404	-1.62528	1.493248					
O	-0.80158	-0.72124	2.292325					
C	-3.79218	1.182979	0.879748					
O	0.938316	0.760923	2.443602					
C	2.296264	-0.2637	0.054122					
C	3.273975	-1.43801	-0.04048					
C	4.471043	-1.3889	-0.62001					
O	-0.11234	2.114972	0.072587					
O	-0.10828	3.325185	1.992203					
C	0.132837	3.233162	0.80228					
C	0.740635	4.32528	-0.03416					
O	2.032496	0.297774	-1.2546					
O	3.20617	2.186635	-0.79641					
C	2.524208	1.520009	-1.54729					
C	2.088551	1.944614	-2.92913					
H	-2.59467	-0.97217	-2.60254					
H	-3.85207	-2.19541	-2.32016					
H	-4.78681	0.017917	-1.66532					
H	-4.53251	-0.94871	-0.22847					
H	-1.16215	0.419281	-1.24041					
H	-1.42994	1.479048	1.518309					
H	0.277637	-1.03164	-1.31134					
H	2.238229	-1.36902	2.437316					
H	0.979723	-2.57709	2.130316					
H	3.381327	-3.25402	1.170448					
H	2.112006	-4.41411	-0.57661					
H	-0.06684	-3.59722	-1.28519					

H	-2.44452	2.699012	-1.08481					
H	-4.12657	2.33839	-1.50668					
H	-2.80261	1.597297	-2.4186					
H	-1.26397	-2.66022	1.676625					
H	-2.62125	-1.53405	1.802876					
H	-3.70217	0.442454	1.680297					
H	-3.37803	2.126938	1.246743					
H	-4.86052	1.351781	0.705956					
H	0.482827	1.573497	2.724314					
H	2.696652	0.529942	0.687695					
H	4.83821	-0.483	-1.09356					
H	5.127753	-2.25383	-0.64141					
H	1.76999	4.046277	-0.28215					
H	0.1905	4.449998	-0.97036					
H	0.738447	5.254287	0.534888					
H	2.655419	2.822467	-3.238					
H	2.226113	1.130466	-3.64449					
H	1.02184	2.189347	-2.90394					

Table S4. Key conformers of compound **2**

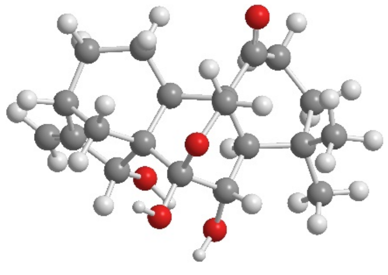	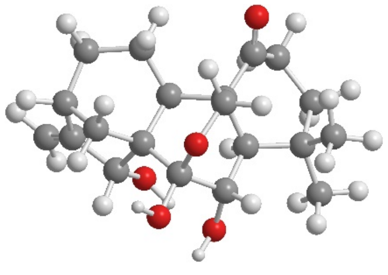
Conformer **2-1** 50.29%	Conformer **2-2** 49.71%

Table S5. Conformers and Bolzmann distributions of the optimized **2**

species	*E'=E+ZPE*	*E*	*H*	*G*	*ΔG*	*ΔE(kcal/mol)*	*p%*
**2-1**	-1154.645495	-1154.623592	-1154.622648	-1154.692742	0	0	50.29%
**2-2**	-1154.6455	-1154.623593	-1154.622649	-1154.692753	1.1E-05	0.006902605	49.71%

E, *E'*, *H, G*: total energy, total energy with zero point energy (ZPE), enthalpy, and Gibbs free energy

Table S6. Optimized Z-matrixes of isomer **2** in the gas phase (Å) at B3LYP/6-31G(d,p) level.

**2-1**					**2-2**			
atom	X	Y	Z		atom	X	Y	Z
C	2.268362	1.95886	-1.43457		C	2.268639	1.958642	-1.43476
C	3.223738	0.736932	-1.56393		C	3.22408	0.736604	-1.56377
C	2.955354	-0.41854	-0.54974		C	2.955455	-0.41873	-0.54956
C	1.419122	-0.45397	-0.29414		C	1.419205	-0.45406	-0.29421

C	0.832053	0.837668	0.373499		C	0.832104	0.837673	0.373272
C	1.622484	2.06639	-0.06764		C	1.622613	2.066331	-0.06795
C	0.855069	-1.65999	0.474081		C	0.85496	-1.66005	0.473898
C	-0.39353	-1.20753	1.258319		C	-0.39349	-1.20746	1.258371
C	-1.40226	-0.37126	0.437192		C	-1.40225	-0.37107	0.437392
C	-0.66496	0.920291	-0.0997		C	-0.66487	0.920254	-0.09985
C	-2.65511	0.022125	1.257545		C	-2.65492	0.022695	1.257796
C	-3.50102	0.715736	0.170712		C	-3.50097	0.716091	0.170937
C	-2.91264	2.13711	-0.08555		C	-2.91237	2.137314	-0.08599
C	-1.41077	2.215883	0.271332		C	-1.41051	2.216056	0.270823
O	1.715269	3.059879	0.633721		O	1.715482	3.059853	0.633351
C	3.413891	-1.74401	-1.18912		C	3.413934	-1.74433	-1.18878
C	3.794864	-0.18356	0.724938		C	3.794813	-0.18373	0.725227
O	0.554795	-2.72588	-0.436		O	0.554488	-2.72574	-0.43634
O	-0.94993	-2.37481	1.806689		O	-0.94954	-2.3748	1.80671
C	-2.10565	-1.10788	-0.7491		C	-2.10589	-1.10783	-0.74877
C	-3.29696	-0.20312	-1.02987		C	-3.29735	-0.20318	-1.02937
O	-1.34329	-1.32796	-1.91736		O	-1.34372	-1.32782	-1.91712
C	-3.99288	-0.20593	-2.16489		C	-3.99375	-0.20654	-2.1641
C	0.831681	0.734805	1.913471		C	0.831707	0.73504	1.913243
O	0.0645	-0.40544	2.344798		O	0.064917	-0.40539	2.344796
H	1.450926	1.84942	-2.16008		H	1.451385	1.849075	-2.16046
H	2.776802	2.900286	-1.65299		H	2.777188	2.899957	-1.65336
H	3.141244	0.345487	-2.5833		H	3.141802	0.34512	-2.58313
H	4.259583	1.072565	-1.44679		H	4.259861	1.072345	-1.44642
H	0.962874	-0.51859	-1.28834		H	0.963134	-0.51865	-1.28849
H	1.558657	-2.02444	1.234277		H	1.558517	-2.02474	1.234007
H	-0.65641	0.846954	-1.19284		H	-0.65642	0.846655	-1.19298
H	-3.16209	-0.87958	1.622051		H	-3.16224	-0.87875	1.622618
H	-2.43712	0.653076	2.121472		H	-2.43676	0.653777	2.121582
H	-4.55974	0.798924	0.435654		H	-4.55958	0.799584	0.436159
H	-3.45739	2.873925	0.51632		H	-3.45707	2.874491	0.515477
H	-3.07338	2.404273	-1.13496		H	-3.07303	2.403954	-1.13555
H	-0.95174	3.070995	-0.23421		H	-0.95143	3.070939	-0.23508
H	-1.30621	2.418418	1.341681		H	-1.30585	2.41901	1.341082
H	3.334984	-2.58088	-0.48892		H	3.334738	-2.58113	-0.48853
H	4.461183	-1.66815	-1.50279		H	4.461296	-1.66866	-1.50225
H	2.811636	-1.9945	-2.06774		H	2.81181	-1.99478	-2.0675
H	3.668817	0.821329	1.13822		H	3.669017	0.821336	1.138164
H	3.558931	-0.90705	1.512452		H	3.55844	-0.90688	1.51291
H	4.858033	-0.29811	0.487283		H	4.857987	-0.29871	0.487817
H	0.127734	-3.41568	0.095301		H	0.127266	-3.41553	0.094837
H	-1.51342	-2.11013	2.546995		H	-1.51754	-2.11018	2.543585

H	-2.4698	-2.07053	-0.35186		H	-2.46982	-2.0705	-0.35139
H	-0.67931	-2.00132	-1.67864		H	-0.67962	-2.0011	-1.67851
H	-3.72753	-0.87305	-2.97865		H	-3.7286	-0.87397	-2.97766
H	-4.83439	0.464063	-2.32163		H	-4.83537	0.463309	-2.32081
H	0.39596	1.636567	2.349289		H	0.395715	1.636785	2.348847
H	1.845268	0.639364	2.310855		H	1.845293	0.640039	2.310746

Table S7. Key conformers of compound **3.**

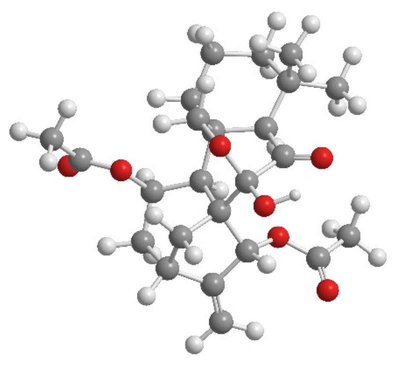

Conformer 3-1 100%

Table S8. Conformers and Bolzmann distributions of the optimized **3.**

species	*E'=E+ZPE*	*E*	*H*	*G*	*ΔG*	*ΔE(kcal/mol)*	*p%*
**3-1**	-1459.901646	-1459.872636	-1459.871692	-1459.960154	0	0	100%

E, *E'*, *H, G*: total energy, total energy with zero point energy (ZPE), enthalpy, and Gibbs free energy

Table S9. Optimized Z-matrixes of isomer **3** in the gas phase (Å) at B3LYP/6-31G(d,p) level.

**3-1**			
atom	X	Y	Z
C	2.268362	1.95886	-1.43457
C	3.223738	0.736932	-1.56393
C	2.955354	-0.41854	-0.54974
C	1.419122	-0.45397	-0.29414
C	0.832053	0.837668	0.373499
C	1.622484	2.06639	-0.06764
C	0.855069	-1.65999	0.474081
C	-0.39353	-1.20753	1.258319
C	-1.40226	-0.37126	0.437192
C	-0.66496	0.920291	-0.0997
C	-2.65511	0.022125	1.257545
C	-3.50102	0.715736	0.170712
C	-2.91264	2.13711	-0.08555
C	-1.41077	2.215883	0.271332
O	1.715269	3.059879	0.633721

C	3.413891	-1.74401	-1.18912
C	3.794864	-0.18356	0.724938
O	0.554795	-2.72588	-0.436
O	-0.94993	-2.37481	1.806689
C	-2.10565	-1.10788	-0.7491
C	-3.29696	-0.20312	-1.02987
O	-1.34329	-1.32796	-1.91736
C	-3.99288	-0.20593	-2.16489
C	0.831681	0.734805	1.913471
O	0.0645	-0.40544	2.344798
H	1.450926	1.84942	-2.16008
H	2.776802	2.900286	-1.65299
H	3.141244	0.345487	-2.5833
H	4.259583	1.072565	-1.44679
H	0.962874	-0.51859	-1.28834
H	1.558657	-2.02444	1.234277
H	-0.65641	0.846954	-1.19284
H	-3.16209	-0.87958	1.622051
H	-2.43712	0.653076	2.121472
H	-4.55974	0.798924	0.435654
H	-3.45739	2.873925	0.51632
H	-3.07338	2.404273	-1.13496
H	-0.95174	3.070995	-0.23421
H	-1.30621	2.418418	1.341681
H	3.334984	-2.58088	-0.48892
H	4.461183	-1.66815	-1.50279
H	2.811636	-1.9945	-2.06774
H	3.668817	0.821329	1.13822
H	3.558931	-0.90705	1.512452
H	4.858033	-0.29811	0.487283
H	0.127734	-3.41568	0.095301
H	-1.51342	-2.11013	2.546995
H	-2.4698	-2.07053	-0.35186
H	-0.67931	-2.00132	-1.67864
H	-3.72753	-0.87305	-2.97865
H	-4.83439	0.464063	-2.32163
H	0.39596	1.636567	2.349289
H	1.845268	0.639364	2.310855

Table S10. Key conformers of compound **4.**

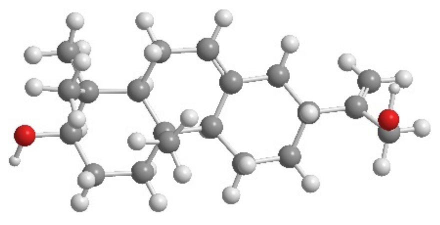	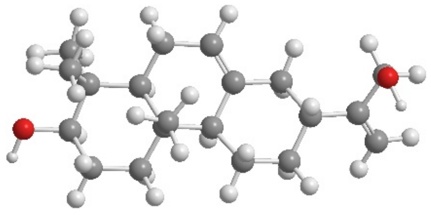
Conformer **4-1** 31.34%	Conformer **4-2** 28.18%
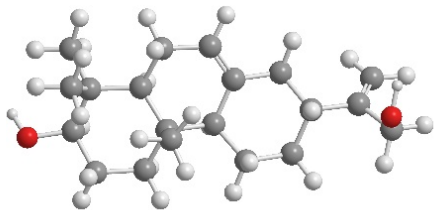	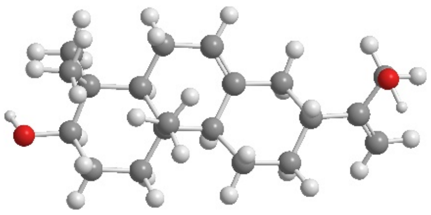
Conformer **4-33** 20.51%	Conformer **4-4** 19.97%

Table S11. Conformers and Bolzmann distributions of the optimized **4.**

species	*E'=E+ZPE*	*E*	*H*	*G*	*ΔG*	*ΔE(kcal/mol)*	*p%*
**4-1**	-931.364933	-931.342655	-931.34171	-931.414655	0	0	31.34%
**4-2**	-931.36495	-931.342687	-931.341742	-931.414555	0.0001	0.06275095	28.18%
**4-3**	-931.36453	-931.342259	-931.341315	-931.414255	0.0004	0.2510038	20.51%
**4-4**	-931.364568	-931.342315	-931.341371	-931.41423	0.000425	0.266691538	19.97%

E, *E'*, *H, G*: total energy, total energy with zero point energy (ZPE), enthalpy, and Gibbs free energy

Table S12. Optimized Z-matrixes of isomer **4** in the gas phase (Å) at B3LYP/6-31G(d,p) level.

**4-1**					**4-2**			
atom	X	Y	Z		atom	X	Y	Z
C	-3.31264	-1.89508	-0.23562		C	-3.3848	-1.82676	0.237326
C	-4.0042	-0.61801	-0.71224		C	-4.02037	-0.6886	-0.56109
C	-3.49747	0.65422	0.014673		C	-3.45889	0.706175	-0.18262
C	-1.92746	0.693869	-0.10468		C	-1.88804	0.648563	-0.28868
C	-1.12403	-0.61571	0.211476		C	-1.14428	-0.56625	0.368239
C	-1.80538	-1.81666	-0.49043		C	-1.87503	-1.87833	-0.00928
C	-1.2855	1.885542	0.628745		C	-1.19661	1.96214	0.118156
C	0.195678	1.953556	0.374383		C	0.286909	1.898928	-0.12197
C	0.915781	0.942979	-0.12769		C	0.962961	0.763194	-0.33382
C	0.309272	-0.42732	-0.40598		C	0.299939	-0.60589	-0.25287
C	2.3541	1.143375	-0.55714		C	2.413316	0.788797	-0.77046
C	3.296508	0.011385	-0.11409		C	3.305386	-0.2478	-0.04134

C	2.70756	-1.33194	-0.59969		C	2.653311	-1.6298	-0.17483
C	1.304916	-1.54888	-0.01912		C	1.245342	-1.62333	0.433629
C	-4.08212	1.87613	-0.73455		C	-3.98917	1.715644	-1.2297
C	-4.05088	0.699327	1.456854		C	-4.01068	1.146965	1.192157
C	-1.01428	-0.88953	1.729993		C	-1.05353	-0.44012	1.907363
C	4.743338	0.18735	-0.54218		C	4.745998	-0.16418	-0.51537
O	-5.42187	-0.69077	-0.54343		O	-5.43967	-0.65546	-0.39379
C	5.762054	-0.58135	0.276337		C	5.560903	0.976378	0.060711
C	5.154378	0.970431	-1.54588		C	5.314311	-1.02514	-1.36714
O	5.750283	-0.2043	1.654443		O	5.663422	0.907359	1.484325
H	-3.73252	-2.75517	-0.77626		H	-3.84039	-2.77916	-0.06889
H	-3.53199	-2.06215	0.8246		H	-3.61331	-1.70534	1.301964
H	-3.76862	-0.49205	-1.78498		H	-3.78079	-0.8547	-1.62738
H	-1.74595	0.871903	-1.17639		H	-1.69695	0.534017	-1.36727
H	-1.64485	-1.7291	-1.57472		H	-1.70829	-2.08074	-1.07719
H	-1.32714	-2.75381	-0.1844		H	-1.43796	-2.72308	0.534723
H	-1.48731	1.838579	1.708042		H	-1.40242	2.207781	1.169348
H	-1.73923	2.824219	0.289697		H	-1.60672	2.797937	-0.46067
H	0.684729	2.909502	0.561421		H	0.818023	2.84905	-0.17876
H	0.190089	-0.49682	-1.50147		H	0.185512	-0.9581	-1.29312
H	2.370467	1.189701	-1.65769		H	2.463649	0.557301	-1.84637
H	2.720564	2.112877	-0.20169		H	2.810729	1.802595	-0.65382
H	3.303797	-0.01704	0.984766		H	3.315424	0.01871	1.025011
H	2.674553	-1.32955	-1.69831		H	2.593313	-1.91416	-1.23492
H	3.353728	-2.1675	-0.30714		H	3.270745	-2.38908	0.319566
H	0.9153	-2.51822	-0.34937		H	0.812813	-2.62809	0.373031
H	1.399276	-1.60193	1.071053		H	1.338072	-1.38383	1.498802
H	-3.66973	1.957873	-1.74704		H	-3.57001	1.517096	-2.22313
H	-5.16661	1.772501	-0.81873		H	-5.07685	1.637227	-1.29911
H	-3.87434	2.813863	-0.21089		H	-3.74408	2.747591	-0.96202
H	-5.13894	0.787096	1.424164		H	-5.09283	1.277004	1.123881
H	-3.82136	-0.19444	2.039879		H	-3.82675	0.422343	1.987476
H	-3.65977	1.563309	2.001267		H	-3.57592	2.101213	1.50213
H	-0.58424	-1.87782	1.91602		H	-2.02295	-0.23937	2.362715
H	-0.37378	-0.1547	2.225541		H	-0.67852	-1.36657	2.351802
H	-1.98462	-0.86826	2.226494		H	-0.37538	0.364441	2.205085
H	-5.7342	-1.45969	-1.03724		H	-5.78529	-1.51303	-0.67263
H	5.535493	-1.6535	0.277283		H	6.554226	1.001101	-0.4112
H	6.762863	-0.45916	-0.16343		H	5.080767	1.940931	-0.13911
H	6.208278	1.0452	-1.80179		H	6.350547	-0.90894	-1.67465
H	4.472552	1.557029	-2.15274		H	4.779159	-1.86658	-1.79506
H	5.928402	0.745694	1.686865		H	6.070491	0.0558	1.695011
**4-3**					**4-4**			

atom	X	Y	Z		atom	X	Y	Z
C	-3.31422	-1.90064	-0.23303		C	-3.3869	-1.83097	0.242869
C	-4.00249	-0.63056	-0.71633		C	-4.01849	-0.70249	-0.56242
C	-3.4972	0.64924	0.016762		C	-3.45809	0.701354	-0.18096
C	-1.92698	0.694847	-0.09772		C	-1.88689	0.650783	-0.28203
C	-1.12403	-0.61662	0.2157		C	-1.14425	-0.56596	0.374161
C	-1.80679	-1.81864	-0.48293		C	-1.8766	-1.87847	0.001315
C	-1.28516	1.881558	0.645251		C	-1.19632	1.962487	0.134705
C	0.196322	1.950601	0.391159		C	0.287555	1.900207	-0.10511
C	0.915264	0.943212	-0.11899		C	0.962488	0.765266	-0.32488
C	0.307746	-0.42518	-0.40451		C	0.298554	-0.60378	-0.25032
C	2.352949	1.146571	-0.54911		C	2.412161	0.793219	-0.7636
C	3.295741	0.011042	-0.11606		C	3.305025	-0.24897	-0.04361
C	2.70543	-1.32852	-0.61015		C	2.65127	-1.62945	-0.18446
C	1.304126	-1.54951	-0.02777		C	1.244887	-1.6259	0.427736
C	-4.07207	1.876271	-0.73179		C	-3.97743	1.715269	-1.22947
C	-4.05562	0.692707	1.455082		C	-4.01637	1.14115	1.189646
C	-1.00855	-0.89248	1.733588		C	-1.04836	-0.44069	1.913213
C	4.741756	0.189681	-0.54579		C	4.744482	-0.16342	-0.52097
O	-5.40905	-0.83706	-0.57452		O	-5.4325	-0.80488	-0.38755
C	5.762054	-0.5804	0.269357		C	5.560553	0.975965	0.055904
C	5.150634	0.975665	-1.54809		C	5.310938	-1.02191	-1.37644
O	5.753318	-0.20481	1.647901		O	5.663323	0.906323	1.479413
H	-3.74371	-2.75237	-0.77145		H	-3.85151	-2.77482	-0.06221
H	-3.5355	-2.06301	0.827746		H	-3.61805	-1.70298	1.306382
H	-3.75684	-0.50885	-1.78684		H	-3.76774	-0.87414	-1.62486
H	-1.74134	0.877876	-1.16815		H	-1.69006	0.540574	-1.36033
H	-1.64268	-1.73716	-1.56717		H	-1.70581	-2.08847	-1.06449
H	-1.32935	-2.75423	-0.1709		H	-1.44099	-2.71944	0.552296
H	-1.48895	1.825516	1.723752		H	-1.40487	2.200502	1.187157
H	-1.73519	2.825034	0.314255		H	-1.6027	2.804005	-0.43862
H	0.685589	2.905219	0.584216		H	0.818883	2.850494	-0.15644
H	0.185319	-0.48808	-1.50001		H	0.180984	-0.95029	-1.29212
H	2.367245	1.201052	-1.64928		H	2.460072	0.569217	-1.8412
H	2.720231	2.113367	-0.18717		H	2.810202	1.806013	-0.64064
H	3.305228	-0.02502	0.982543		H	3.318147	0.01077	1.02437
H	2.669958	-1.31821	-1.70862		H	2.588265	-1.90675	-1.24622
H	3.352146	-2.16624	-0.32515		H	3.269248	-2.39247	0.303362
H	0.913212	-2.51612	-0.364		H	0.811248	-2.62971	0.361823
H	1.401435	-1.61037	1.061717		H	1.34065	-1.39309	1.494107
H	-3.71515	1.919754	-1.76737		H	-3.61346	1.476996	-2.2356
H	-5.16788	1.844991	-0.75194		H	-5.07358	1.721974	-1.25827
H	-3.80359	2.817559	-0.24472		H	-3.67299	2.739234	-0.99639

H	-5.14742	0.717527	1.419557		H	-5.10538	1.212591	1.134061
H	-3.78124	-0.178	2.052067		H	-3.78977	0.440153	1.994162
H	-3.71404	1.585216	1.986934		H	-3.62864	2.121689	1.479871
H	-0.56988	-1.87772	1.914452		H	-0.66454	-1.3655	2.353106
H	-0.37333	-0.15441	2.231043		H	-0.37554	0.368141	2.211321
H	-1.9778	-0.8837	2.232405		H	-2.01761	-0.25114	2.373601
H	-5.86567	-0.1272	-1.04302		H	-5.85724	-0.21602	-1.02375
H	5.534957	-1.65243	0.269573		H	6.553759	1.00002	-0.41628
H	6.76195	-0.45804	-0.17241		H	5.08143	1.94116	-0.14335
H	6.204037	1.051751	-1.80563		H	6.346534	-0.90482	-1.68576
H	4.467427	1.563379	-2.15235		H	4.77498	-1.86222	-1.80554
H	5.933119	0.744838	1.681071		H	6.070217	0.054608	1.689809
